# Impact of Structural Features on the Antioxidant Activity of Organofluorine Diaryl Hydrazones

**DOI:** 10.3390/molecules31010078

**Published:** 2025-12-24

**Authors:** Zsuzsanna K. Zsengellér, Maxim Mastyugin, Adrianna R. Fusco, Bernadett R. Vlocskó, Coryn Ferguson, Diana Pintye, Hamad Nasim, Saira Salahuddin, Brett C. Young, Béla Török, Marianna Török

**Affiliations:** 1 Department of Medicine, Beth Israel Lahey Health, Boston, MA 02115, USA; zzsengel@bidmc.harvard.edu (Z.K.Z.); cfergus2@bidmc.harvard.edu (C.F.);; 2 Department of Chemistry, University of Massachusetts Boston, Boston, MA 02125, USA; bernadett.vlocsko001@umb.edu; 3 Department of OB/GYN, Beth Israel Lahey Health, Boston, MA 02115, USA; ssalahud@bidmc.harvard.edu; 4 Department of OB/GYN, Mt Auburn Hospital, Cambridge, MA 02138, USA

**Keywords:** preeclampsia, oxidative stress, organofluorine, hydrazones, antioxidants

## Abstract

Preeclampsia (PE) affects 2–8% of pregnancies, yet it lacks curative treatment options. Oxidative stress caused by the release of reactive oxygen and nitrogen species (ROS/RNS) in the placenta is common in abnormal placental development. It can cause downstream signaling and the formation of anti-angiogenic factors, e.g., soluble fms-like tyrosine kinase 1 (sFLT-1), leading to symptoms of PE, such as hypertension, proteinuria, and, in severe cases, eclampsia. Mitochondria-targeted antioxidants were developed to reduce oxidative stress and alleviate PE symptoms. Ten organofluorine diaryl hydrazones were designed as potential antioxidants, synthesized, and tested for their activity using the 2,2-diphenyl-1-picrylhydrazyl (DPPH), 2,2′-azino-bis(3-ethylbenzothiazoline-6-sulfonic acid) (ABTS), and oxygen radical absorbance capacity (ORAC) assays. Compounds **2**, **3**, **5**, and **6** showed excellent antioxidant capacity in all three assays and were tested in an in vitro human trophoblast cell culture system mimicking PE in which the cells were exposed to oxidative stress inducing the release of sFLT-1. The anti-angiogenic factor sFLT-1 was greatly reduced in cells treated with antioxidants. Compounds **5** and **6** were more effective in preventing sFLT-1 release than **2** and **3**. Density functional theory calculations of the electronic structures of compounds **2**, **5,** and **6** were conducted at the M06-2X/6-311G+(d,p) level to further understand the reactivity profile of these molecules. The electron density of delocalized bonds (EDDB(r)) was calculated to analyze the effect of delocalization on radical stabilization.

## 1. Introduction

Preeclampsia (PE) is a hypertensive disorder impacting 2–8% of pregnancies worldwide [1,2,3,4]. It is characterized by new-onset hypertension following 20 weeks’ gestation accompanied by proteinuria and/or features of end organ damage such as raised liver enzymes, thrombocytopenia, and elevated serum creatinine [5]. When PE is accompanied by the development of grand mal seizures, it is termed eclampsia. PE and eclampsia are one of the leading causes of maternal morbidity and mortality and are estimated to account for 50,000 maternal deaths each year worldwide. Long-term maternal complications of PE include chronic hypertension, diabetes mellitus, chronic renal failure, and coronary artery disease, while fetal complications include premature birth, intrauterine fetal growth restriction, neonatal respiratory distress syndrome, and fetal death [6]. The pathogenesis of PE begins with abnormal placentation, which occurs due to defective spiral artery remodeling [7]. This is mediated by invading cytotrophoblasts which induce fibrinoid necrosis in the vessel walls, leading to a loss of muscular and elastic tissue [8,9].

Defective remodeling results in placental ischemia, leading to repetitive reperfusion injury due to a rise in reactive oxygen species (ROS). This creates an environment of oxidative stress which leads to intensified lipid peroxidation causing increased platelet adhesion and vasoconstriction. Elevated levels of ROS also stimulate the production of several anti-angiogenic factors such as soluble fms-like tyrosine kinase-1 (sFLT-1) [1,7,10,11]. sFLT-1 is an antagonist of vascular endothelial growth factor (VEGF) and placental growth factor (PIGF), which blocks their activity on endogenous receptors. This leads to maternal endothelial dysfunction and clinical features of preeclampsia [8,9,12,13,14].

As of today, the only definitive cure for preeclampsia is the delivery of fetus. The pathology of preeclampsia is linked to oxidative stress, therefore small-molecule antioxidants could potentially be used as novel therapeutics. Mitochondria-targeting therapies have been proposed to reduce oxidative stress and mitigate preeclampsia symptoms. The mitochondrion is a key site for ROS production, primarily in the electron transport chain during oxidative phosphorylation, making it a prime targeting site for these treatments [15,16,17]. Additionally, the mitochondrial enzyme aconitase (part of the Krebs cycle) contains a ferrous ion and is sensitive to oxidative stress, which can lead to mitochondrial damage and an increased production of ROS [17,18].

Small-molecule antioxidants are stable molecules that commonly have a high degree of conjugation, which can scavenge radicals by donating either a hydrogen atom or an electron [19]. Certain compounds such as vitamin C (ascorbic acid) and vitamin E are well-known for their radical scavenging abilities but have not been found to be beneficial in decreasing the rates of preeclampsia in at-risk women, potentially due to their low mitochondrial uptake [20,21,22,23,24,25,26]. A compound that has shown promising antioxidant ability to alleviate PE symptoms was resveratrol, a polyphenolic compound that is found in plants and certain fruits [27]. It has been proven to have antioxidant, anti-inflammatory, neuroprotective, cardioprotective, immunomodulatory, antiplatelet, and anticancer properties [27,28]. However, as with many exogenous small-molecule antioxidants, due to its poor bioavailability, rapid elimination from the body, and fetal adverse effect in the form of increased pancreatic mass, it is not considered a suitable therapeutic option for PE.

Diaryl hydrazones were designed as novel therapeutics for preeclampsia based on their excellent radical scavenging characteristics and remarkably low toxicity [29]. Most of the antioxidants included fluorine because they help increase metabolic stability and absorption of the compounds [30]. The diaryl hydrazones are designed with different substituents, such as hydroxyl groups, which can stabilize a free radical. In our study, the radical scavenging abilities of the synthesized hydrazones were tested using multiple biochemical and cell-based assays that rely on different radical scavenging mechanisms [31,32,33,34].

## 2. Results

### 2.1. Biochemical Analysis of the Synthesized Diaryl Hydrazones

The radical scavenging ability of the ten synthesized diaryl hydrazones of interest were evaluated in the 2,2-diphenyl-1-picrylhydrazyl (DPPH), 2,2′-azino-bis(3-ethylbenzothiazoline-6-sulfonic acid) (ABTS), and oxygen radical absorbance capacity (ORAC) assays to determine which compounds had the best antioxidant capacities in comparison to the reference antioxidants, Trolox and ascorbic acid. Based on our earlier works [26,29,35], the emphasis was placed on hydrazones with fluorine-containing substituents, such as aryl-F and CF_3_. The structures of the synthesized diaryl hydrazones are depicted in Table 1.

Hydrazones are generally valued for their stability and ease of synthesis. The most common hydrazone synthesis method involves the condensation of hydrazine with an aldehyde or ketone under mild heating or reflux conditions, usually in the presence of an acid catalyst. Our green protocol builds on the traditional method by using hydrazines and the corresponding ketones or aldehydes as building blocks but using microwave irradiation instead of conventional heating for activation. This protocol enables rapid (10–15 min) hydrazone formation without needing any further additives or a catalyst, using only ethanol and water as the benign medium. The detailed procedure is included in the Section 4.

**Figure 1:**
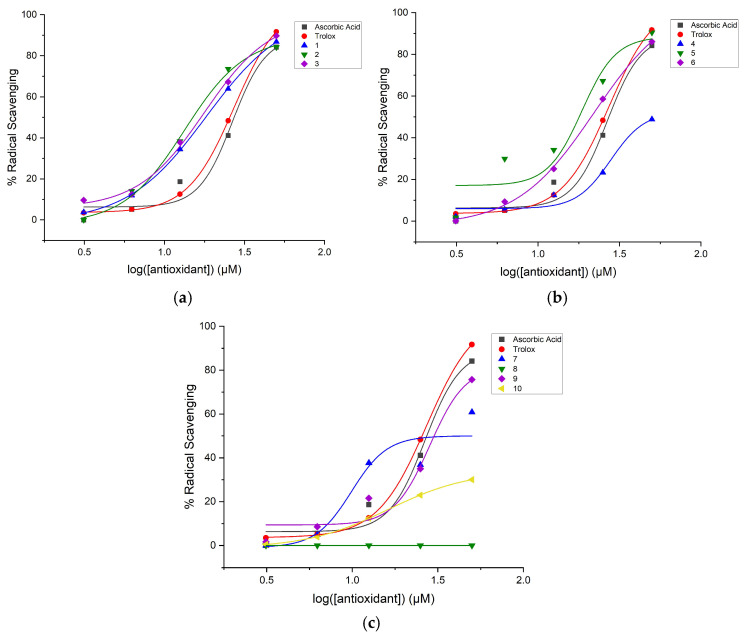
Dose–response curve with nonlinear regression fitting using data from the DPPH assay of the hydrazones with the reference antioxidants (Trolox and ascorbic acid): (**a**) Compounds **1**–**3**, (**b**) Compounds **4**–**6**, and (**c**) Compounds **7**–**10**. The data are shown as the mean of radical scavenging, where the number of independent repeats is three.

**Figure 2:**
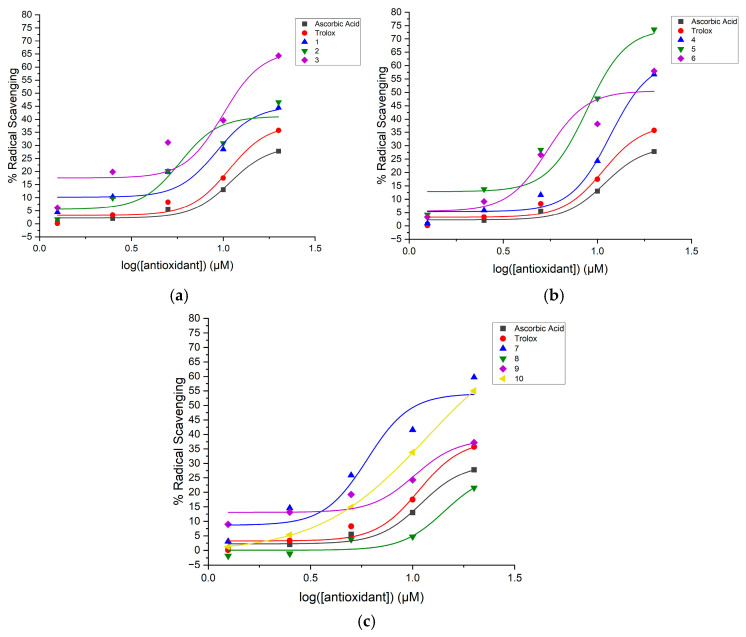
Dose–response curve with nonlinear regression fitting using data from the ABTS assay of the hydrazones with the reference antioxidants (Trolox and ascorbic acid): (**a**) Compounds **1**–**3**, (**b**) Compounds **4**–**6**, and (**c**) Compounds **7**–**10**. The data are shown as the mean of radical scavenging, where the number of independent repeats is three.

**Figure 3:**
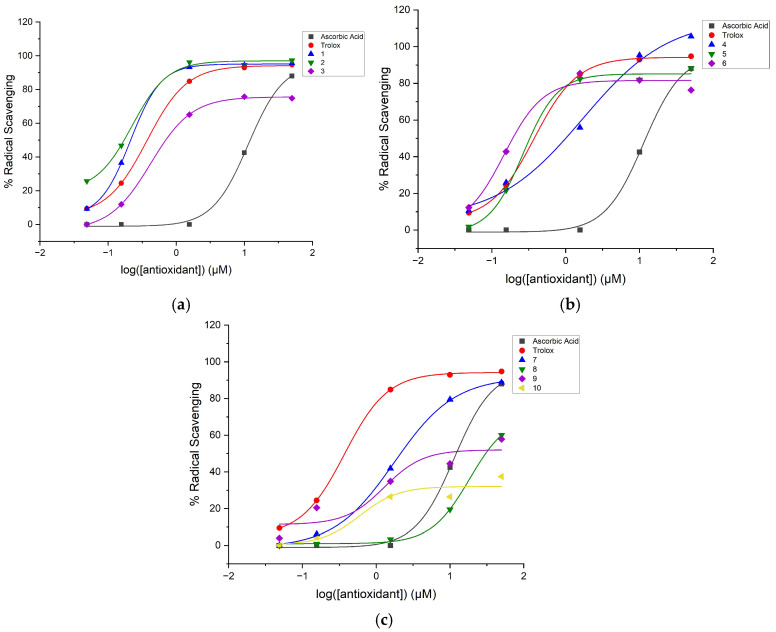
Dose–response curve with nonlinear regression fitting using data from the ORAC assay of the hydrazones with reference antioxidants (Trolox and ascorbic acid): (**a**) Compounds **1**–**3**, (**b**) Compounds **4**–**6**, and (**c**) Compounds **7**–**10**. The data are shown as the mean of radical scavenging, where the number of independent repeats is three.

The radical scavenging abilities of the compounds were evaluated in the DPPH, ABTS, and ORAC assays by finding their half-maximal effective concentration (EC_50_) values. EC_50_ is the compound concentration where the radical scavenging activity is the average between its minimum and maximum radical scavenging activities and was calculated using nonlinear regression fit with a four-parameter Hill equation, Equation (1) [36,37].

(1) $$y=A_{1}+\frac{A_{2}-A_{1}}{1+10^{\left[\left(log{\;x}_{0}-\log x\right)p\right]}}$$

where *y* is the percent radical scavenging; *A*_1_, the bottom asymptote, is the minimum percent radical scavenging (baseline); *A*_2_, the top asymptote, is the maximum percent radical scavenging; *x*_0_ is the inflection point representing the logEC_50_ value; *x* is the antioxidant concentration; and *p* is the Hill slope. Figure 1, Figure 2 and Figure 3 show the dose–response curves with the nonlinear regression fitting found through Equation (1) for the diaryl hydrazones and the reference antioxidants in the DPPH, ABTS, and ORAC assays, respectively.

The relative EC_50_ values of the compounds were calculated using Equation (1) with the parameters given by the nonlinear regression fitting of the dose–response curves in Figure 1, Figure 2 and Figure 3. If the compounds have lower relative EC_50_ values, they are considered to exhibit higher radical scavenging activity. Table 2 presents the calculated EC_50_ values of the organofluorine hydrazones along with the reference antioxidants in the DPPH, ABTS, and ORAC assays. The maximum radical scavenging values (E_max_ in percentiles) determined under our assay conditions are also listed to provide more context for the efficacy of the compounds.

Table 2 highlights that the hydrazones show equal or better radical scavenging activity than that of the reference antioxidants in all assays. Compounds **2**, **3**, **5**, and **6** are the best-performing hydrazones by having the lowest overall relative EC_50_ values in the assays. These data show the potential these hydrazones have for being powerful antioxidants even at low concentrations. Compounds **4**, **8**, **9**, and **10** are the worst-performing hydrazones with **8** having no radical scavenging activity in the DPPH assay.

### 2.2. sFLT-1 Expression in Extravillous Trophoblast Cells Treated with **2**, **3**, **5**, and **6**

**Figure 4:**
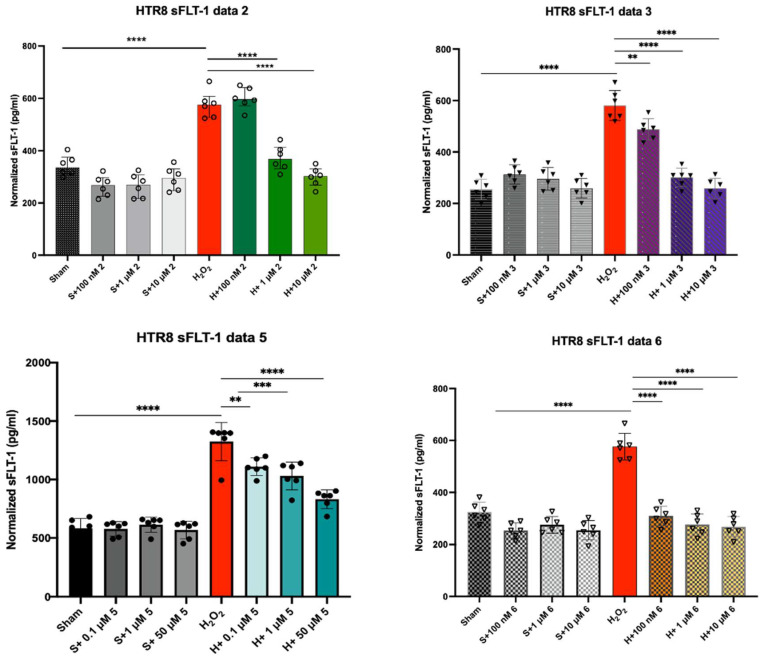
Antioxidant pre-treatment reduced sFLT-1 protein expression in H_2_O_2_-exposed human trophoblast cells. sFLT-1 ELISA (average of three experiments) in HTR8 cells pre-treated with compounds **2**, **3**, **5**, and **6**, respectively, for 30 min, and then exposed to H_2_O_2_ or sham. Mann–Whitney U test, median [IQR]. Mean ± SEM. ****: *p* < 0.0001; ***: *p* < 0.001; and **: *p* < 0.01.

Since compounds **2**, **3**, **5**, and **6** exhibited the best radical scavenging abilities in the ABTS, DPPH, and ORAC assays, the ability of these compounds to downregulate the expression of sFLT-1 was evaluated using sFLT-1 ELISA. The sFLT-1 ELISA assay was used to quantify the amount of sFLT-1 production in HTR8/SVneo human trophoblast cells that were pre-treated with **2**, **3**, **5**, and **6** and exposed to H_2_O_2_ to induce oxidative stress. If the compounds were effective treatments for oxidative stress in preeclampsia, the production of sFLT-1 was expected to be lower in cells pre-treated with the compounds compared to cells exposed to H_2_O_2_ without any intervention. The sFLT-1 assay was used with healthy cells, cells treated with different concentrations of the antioxidants, cells treated with H_2_O_2_, and cells treated with both antioxidants and H_2_O_2_. Healthy cells were used as a control group to quantify normal sFLT-1 production. Cells treated only with H_2_O_2_ (Figure 4, red column) were used to determine the sFLT-1 expression in unprotected cells. Figure 4 shows sFLT-1 production under the above-mentioned conditions.

Figure 4 indicates that antioxidant pre-treatment with compounds **2**, **3**, **5**, and **6** reduced sFLT-1 expression in trophoblast cells exposed to H_2_O_2_. Pre-treatment with the antioxidants at higher concentrations dramatically decreased sFLT-1 production resulting in sFLT-1 values that were either better or nearly equivalent to those of healthy cells. The sFLT-1 reduction profiles of **2**, **3**, and **5** are nearly identical, while **6** decreased the production of the protein even at low concentrations. Compound **6** reduced the sFLT-1 level to that of the normal cells even in a concentration as low as 100 nM, indicating a highly effective antioxidant (Figure 4). Pre-treatment with **5** did not return sFLT-1 production to the same values as healthy cells without induced oxidative stress, but the sFLT-1 production still decreased systematically as the concentration of **5** treatment increased. The sFLT-1 ELISA results showed that all four antioxidants downregulated the expression of sFLT-1 in a cellular model, suggesting that these antioxidants could treat symptoms of oxidative stress and carry potential to decrease oxidative stress in vivo.

### 2.3. Mitochondrial-Derived ROS Production in H_2_O_2_-Exposed Trophoblast Cells Pre-Treated with Selected Antioxidants

**Figure 5:**
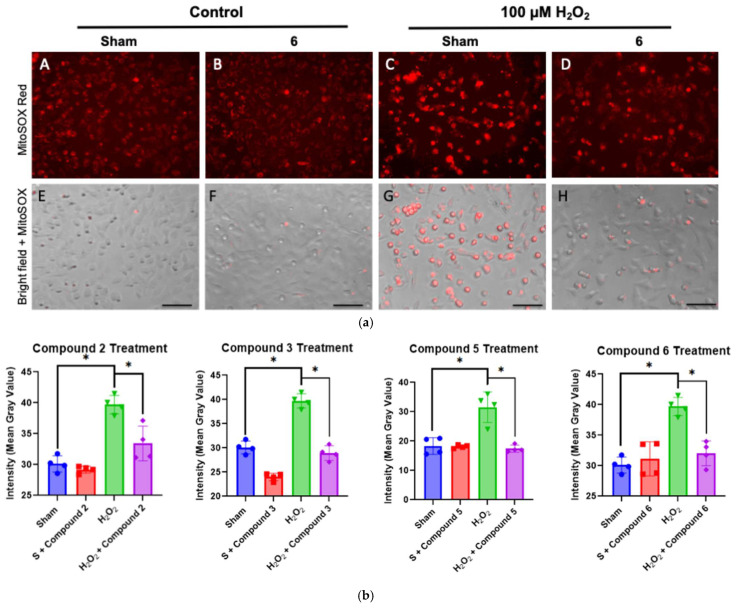
Antioxidant pre-treatment (with compounds **2**,**3**,**5**,**6,** respectively) reduced mitochondrial-derived ROS production in H_2_O_2_-exposed trophoblast cells (HTR8). (**a**) Fluorescence microscopy analysis of MitoSOX Red (25 nM) ((**A**–**D**), superoxide production) in HTR8 cells treated with 50 μM **6** for 30 min, and then exposed to 18 h H_2_O_2_. (Bars: 50 µm). (**E**–**H**) MitoSOX Red images overlayed on bright field images of the trophoblast cell culture. (**b**) Quantitation of MitoSOX Red fluorescence in trophoblast cells treated with compounds **2**, **3**, **5**, and **6**: OD per area (pixels^2^) was calculated in four high-power fields per sample (*n* = 4 per group). * *p* < 0.05 by Mann–Whitney non-parametric test (bars: 100 µm).

The ability of the compounds to prevent oxidative stress was further evaluated by analyzing the release of mitochondrial superoxide in trophoblast cells treated with compounds **2**, **3**, **5**, and **6** before exposure to H_2_O_2_. The mitochondrial superoxide production in the cells was measured using the MitoSOX Red assay. The assay was used with healthy cells, cells pre-treated with antioxidants, cells exposed to H_2_O_2_, and cells treated with an antioxidant before exposure to H_2_O_2_. The healthy cells and cells treated with H_2_O_2_ were used as control groups to determine if antioxidant treatment could reduce oxidative stress. Ideally, cells pre-treated with compounds should have a fluorescence pattern similar to that of healthy cells. If the cells treated with the antioxidant exhibit less bright red fluorescence than cells experiencing oxidative stress without intervention, this suggests that the antioxidant is reducing oxidative stress. Figure 5a shows the red fluorescence patterns of healthy cells, cells treated with **6**, cells treated with H_2_O_2_, and cells treated with **6** and H_2_O_2_, and Figure 5b depicts the quantitative comparison regarding the effect of the four compounds.

Figure 5a shows MitoSOX Red fluorescence microscopy analyses (A–D) and images of the MitoSOX Red fluorescence overlaid on bright field images of trophoblast cells (E–H). The bright field images help assess cellular health. Spread-out cells indicate healthy cells, while rounded cells or those with concavities suggest cell death or impending death. From the images in Figure 5a, adding antioxidants did not damage the cells or significantly increased mitochondrial superoxide production. When H_2_O_2_ was added, the red fluorescence increased, indicating extensive mitochondrial superoxide production. Figure 5b quantifies the red fluorescence signal, showing the highest intensity per cell area when oxidative stress was induced by H_2_O_2_ treatment (Figure 5b, green column). Pre-treatment with antioxidants reduced the red fluorescence, as seen both in the images and in the graphs. The average fluorescence intensity per cell area was nearly the same as in cells not experiencing oxidative stress (Figure 5b, purple column). The brightfield image, H, shows that the cells were well-grown and healthy. The fluorescence data support that all four compounds can reduce mitochondrial superoxide production to levels similar to healthy cells, thereby decreasing oxidative stress, a key factor in the development of preeclampsia.

### 2.4. Theoretical Investigations

**Figure 6:**
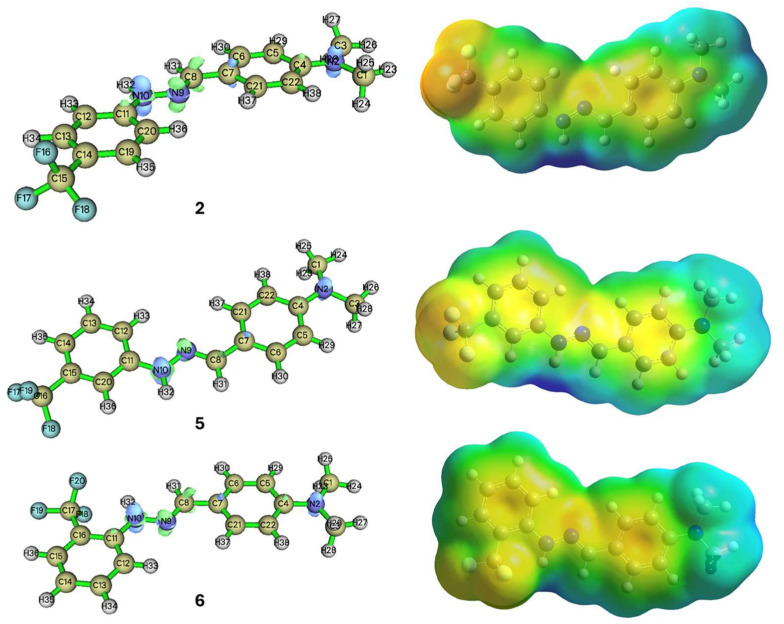
Fukui local reactivity functions and EPS of compounds **2**, **5**, and **6**. In all three derivatives, -NH within the hydrazone group shows the highest susceptibility to electrophilic (free radical) attack.

Compounds **2**, **5**, and **6** showed a promising activity profile in the biochemical and cell biology assays and therefore they were subjected to further analysis to gain insight into how the position of the -CF_3_ group affects the scavenging activity of the compounds. To analyze local reactivity patterns, Fukui functions and the electron potential surface (EPS) were calculated (Equation (2)) and are presented in Figure 6.

(2) $$Dual\;descriptor:\;∆f\left(r\right)=f^{+}\left(r\right)+f^{-}\left(r\right)=\rho_{N+1}\left(r\right)-2\rho_{N}\left(r\right)-\rho_{N-1}\left(r\right)$$

where f^+^ is a Fukui function describing susceptibility towards nucleophilic attack, and f^−^ towards the electrophilic attack.

Although the differences between the three derivatives are subtle, compounds **5** (me-ta-CF_3_) and **6** (ortho-CF_3_) show a slightly more nucleophilic character compared to compound **2** (para-CF_3_), suggesting higher potency. This is aligned well with the experimental results for compound **6** recorded in ABTS and ORAC assays. As a negative reference, we have also calculated the dual descriptor values for compounds **8** and **10**, which showed low activity in the scavenging assays. Based on the Fukui analysis, they also showed lower susceptibility towards electrophiles (Table 3) on the secondary nitrogen.

The primary requirement for antioxidants to effectively scavenge free radicals in the body is that they form a more stable radical as part of the antioxidant protection that can be eliminated by excretion. To design such compounds, one key property is the presence of extended spin delocalization. By delocalizing the unpaired electron, the spin density spreads over multiple atomic centers and therefore reduces radical reactivity. To get a sense of the electron distribution within the compounds, we looked at the Mulliken partial atomic charges estimated from electronic structure calculations [38]. Based on the data summarized in Table 3, in all five cases, the electron density decreases as the radical forms. Compound **6** shows the smallest change in charge as the radical forms. The difference in spin density on the radical N of the five derivatives is highly similar.

To study delocalization in compounds **2**, **5**, and **6** as a function of the position of the -CF_3_ group, the electron density of delocalized bonds (EDDB(r)) descriptor was calculated with its dissected π and σ components [39]. In addition, we included compounds **8** and **10** as negative controls, as these compounds were the worst-performing antioxidants in the radical scavenging assays. We hypothesized that the scavenging activity could be correlated with the level of delocalization post scavenging that would stabilize the formed radical. The results are summarized in Table 4.

The data in Table 4 show a relatively similar electron distribution in the compounds studied except compound **8** that shows a notable difference.

## 3. Discussion

The above data indicate clear directions regarding the radical scavenging effect of the hydrazones. First, most compounds exhibited better or equal antioxidant efficacy than the reference compounds ascorbic acid and Trolox in the radical scavenging assays (Table 2). As outliers, compounds **8** and **9** appeared to be similar or less efficient than the controls, along with compounds **1**, **4**, **7**, and **10**, which were found to show comparable scavenging activities to those of the controls, though our comparison mostly relies on the activities of Trolox, which is a considerably better antioxidant than ascorbic acid. These investigations established four compounds, **2**, **3**, **5**, and **6**, as the leading antioxidants in this set of organofluorine hydrazones. Based on these observations, it can be suggested that the high efficacy is tied to a certain arrangement of substituents in the structure. In general, the synthesis can be carried out by a condensation reaction of a substituted benzaldehyde (aldehyde end) and a phenyl hydrazine (hydrazine end). The data suggest that the compounds exhibit excellent radical scavenging activity when they possess an electron donating group at their aldehyde end (EDG, such as -N(CH_3_)_2_, and 2-OH, 4-OCH_3_, Table 1), and a strong electron withdrawing group (EWD, CF_3_, Table 1) at their hydrazine end. Compounds **2**, **3**, **5**, and **6** belong to this group. Compounds with two EWG groups at both ends show only moderately better results than the controls. Compound **4** is an anomaly and shows similar performance to the control compounds. Based on the radical scavenging assays, the first group was selected for cell-based assays. The results of the sFLT-1 protein expression experiments in H_2_O_2_-exposed human trophoblast cells revealed that the pre-treatment with the selected compounds (**2**, **3**, **5**, and **6**), respectively, reduced the overall sFLT-1 expression and maintained cell viability. The quantitative data (Figure 4) suggest that the compounds are about equally effective in this regard. This is in accordance with their high level of structural similarity. As Figure 5 shows, the MitoSOX Red immunofluorescence assay confirmed this observation: the pre-treatment with compounds **2**, **3**, **5**, and **6**, respectively, reduced mitochondrial-derived ROS production in H_2_O_2_-exposed trophoblast cells (HTR8). The quantitative comparisons (Figure 5b) showed excellent protection from ROS in a similar extent to the sFLT-1 protein expression data. With all experimental data pointing in the same direction, we can conclude that the nearly ideal structure for antioxidants with the hydrazone core structure has been identified, with the compound having an EDG substituent on the aldehyde end and an EWG substituent on the hydrazine end. It appears that the N,N-dimethylamino group as an EDG and the CF_3_ group as an EWG are providing the best improvements. DFT calculations offered further support to these observations. The electronic structure of the three compounds (**2**, **5**, **6**), all being regioisomers, in terms of the positioning (*ortho*, *meta,* or *para* isomers) of the CF_3_ group at the hydrazine end, looks nearly identical. The electron density maps show high similarity, indicating an electron-rich part at the aldehyde end (blue color at the right of the molecules in Figure 6) due to the presence of the -N(CH_3_)_2_ group, and an electron-deficient part at the hydrazine end (yellow-red color at the left in Figure 6) due to the CF_3_ group. The reactivity descriptors (Table 3) indicate a clear distinction between effective (**2**, **5**, **6**) compounds and largely ineffective negative controls (**8** and **10**). The effective compounds exhibit a lower dual descriptor value indicating a stronger nucleophilic character for these compounds. The negative controls provided an about 20–25% higher value, showing lower nucleophilicity for these compounds. The numerical electron density values (Table 3) or the density of delocalized bonds (EDDB(r)) descriptors (Table 4) clearly support the high level of electronic similarity, the values only differ in a few, often only 1%. Based on the EDDB values, no significant trend, that would distinguish based on experimental efficacy, appeared. The same level of delocalization is present in all four derivatives, compounds **2**, **5**, **6**, and **10**, to stabilize the formed hydrazone N-radical. Regarding compound **2**, there is a slightly higher σ contribution to the system. In compound **8**, where the substitution pattern is the most different among the ten derivatives, the highest delocalized electron population on the defined fragment was observed. (EDDB values for all derivatives can be found in the Supplementary Materials.

Both the experimental and theoretical investigations indicate that these compounds are nearly identical in their efficacy at these levels of the investigation. While the further selection of the most effective candidate is to be determined by future investigations, such as animal experiments, having three identically effective compounds could prove advantageous in further trials.

## 4. Materials and Methods

### 4.1. Materials

Substituted hydrazone derivatives were synthesized according to the procedure described below. The carbonyl compounds and substituted hydrazines were all commercially available products and were used without further purification.

Organic solvents (ethanol and dimethyl sulfoxide (DMSO)), salts for buffer solutions (Na_2_HPO_4_, NaH_2_PO_4_, and NaCl), potassium persulfate for 2,2′-bis-(3-ethylbenzothiazoline-6-sulphonic acid) (ABTS) radical generation, reference antioxidants (Trolox and ascorbic acid), radical compounds (DPPH, ABTS, and 2,2′-azobis(2-amidinopropane) dihydrochloride (AAPH)), and MitoSOX Red were purchased from Sigma-Aldrich (St. Louis, MO, USA) Oakwood Chemicals (Estill, SC, USA), or ThermoFisher Scientific (Waltham, MA, USA). The phosphate-buffered saline (PBS), RPMI-1640 cell culture medium, fetal bovine serum (FBS), trypsin, and penicillin–streptomycin were purchased from Gibco (Waltham, MA, USA), or Invitrogen (Waltham, MA, USA). The human trophoblast HTR8/SVneo cell line was from the American Type Culture Collection (ATCC) (Manassas, VA, USA). The ELISA VEGF receptor 1 (VEGFR1) Quantikine kit and the Cell Counting Kit-8 assay were purchased from R&D Systems (Minneapolis, MN, USA) and Dojindo Molecular Technologies, Inc. (Rockville, MD, USA).

### 4.2. Synthesis

A solution of the aldehyde (1 mmol) in 2 mL of ethanol was prepared, followed by the addition of the corresponding hydrazine or hydrazine salt (1 mmol) and 1 mL of water. The reaction mixture was subjected to microwave (MW) irradiation at 80 °C for 10 min at 250 W. Upon completion, the mixture was allowed to cool to room temperature and then placed in the freezer at −5 °C for 15 min to facilitate product precipitation. In some cases, additional water was added to promote crystallization. The resulting precipitate was collected by vacuum filtration and dried. In most cases, no further purification was necessary; if required, the crude product was purified by flash chromatography using a 35:65 ethyl acetate/hexane solvent system. The pure product was characterized by ^1^H, ^13^C, ^19^F NMR, and HRMS.

### 4.3. Biochemical Assays

The synthesized molecules and the reference antioxidants (ascorbic acid and Trolox) were dissolved in dimethyl sulfoxide (DMSO) to an initial concentration of 100 mM and then diluted to the desired stock concentrations through a serial dilution.

For the DPPH assay, the compound stock solutions (25 mM, 12.5 mM, 6.25 mM, 3.13 mM, and 1.56 mM) were diluted in 37 °C 50% ethanol by a factor of 50. DPPH was dissolved in 50 mL of 37 °C 50% ethanol to a concentration of 222 µM and stirred gently in the dark for 45 min to create the DPPH radical solution. On a clear 96-well plate, 20 µL of each compound and 180 µL of DPPH were added in triplicates. Sets of 20 µL of DMSO, that were previously diluted in 37 °C 50% ethanol by a factor of 50, and 180 µL of 37 °C 50% ethanol was used to determine the background absorbance. Sets of 20 µL of DMSO, that were previously diluted in 37 °C 50% ethanol by a factor of 50, and 180 µL of DPPH radical solution were used as the control group. The absorbance values were collected using a VersaMax UV-Vis plate reader (Molecular Devices, San Jose, CA, USA) set to 37 °C and a wavelength of 519 nm. The absorbance readings were recorded every 15 min for 60 min and processed using the SoftMax Pro 5 software.

For the ABTS assay, the compound stock solutions (100 mM, 50 mM, 25 mM, 1.25 mM, and 6.25 mM) were diluted in ethanol by a factor of 100. The ABTS radical solution was prepared by dissolving ABTS and K_2_S_2_O_8_ in deionized water to a concentration of 7 mM and 2.45 mM, respectively. The radical solution was incubated in the dark at room temperature for 16 to 24 h before the assay was started. The absorbance values were collected by a VersaMax UV-Vis plate reader (Molecular Devices, San Jose, CA, USA) that was set to 37 °C and a wavelength of 734 nm. The data were processed using the SoftMax Pro 5 software. The ABTS radical solution was diluted using a 37 °C 75 mM phosphate buffer with 50 mM NaCl at pH 7.4 until the absorbance was between 0.70 and 0.85 (the ratio was typically 5 µL of ABTS and 195 µL of 37 °C phosphate buffer). On a clear flat-bottomed 96-well plate, 4 µL of each compound and 196 µL of ABTS were added in triplicates. Sets with 4 µL of DMSO, that was previously diluted by a factor of 100 in ethanol, and 196 µL of 37 °C phosphate buffer were used to measure the background absorbance. Sets with 4 µL of DMSO, that was previously diluted in ethanol by a factor of 100, and 196 µL of ABTS were used as the control group. The absorbance readings were measured at 0, 6, and 12 min.

The absorbance values from the DPPH and ABTS assays were processed using Equation (3) to determine the radical scavenging ability of each compound. In this equation, *Abs_c_* is the absorbance of the control at the final timepoint and *Abs_t_* is the absorbance of the test compound at the final timepoint.

(3) $$Percent\;Radical\;Scavenging=\frac{\left({Abs}_{c}-{Abs}_{t}\right)}{{Abs}_{c}}\times 100$$

For the ORAC assay, the compound stock solutions (50 mM, 10 mM, 1.56 mM, 0.156 mM, and 0.0488 mM) were diluted with ethanol by a factor of 125. Fluorescein was diluted with the 75 mM phosphate buffer at a pH of 7.4 to a concentration of 0.0816 mM and incubated for two hours at 37 °C before the assay started. The AAPH radical solution was produced by dissolving AAPH (153 mM) in 8 mL of cold (4 °C) 75 mM phosphate buffer at a pH of 7.4. The AAPH radical solution was kept in the dark on ice. On a black 96-well plate, 25 µL of each compound and 150 µL of fluorescein were added in triplicates. Sets of 25 µL of DMSO, that was previously diluted in ethanol by a factor of 125, and 150 µL of fluorescein were used either as the control group (AAPH added later) or to determine the maximum fluorescence (cold 75 mM phosphate buffer at a pH of 7.4 added later). Sets of 25 µL of DMSO, that was previously diluted in ethanol by a factor of 125, and 150 µL of 37 °C 75 mM phosphate buffer at a pH of 7.4 were used to determine the background fluorescence. The plate was incubated at 37 °C for 15 min. A total of 25 µL of the AAPH radical solution was added to each of the wells, except for the wells used to measure the maximum fluorescence, where 25 µL of the cold 75 mM phosphate buffer at a pH of 7.4 was added instead. The fluorescence intensities were collected using a SpectraMax i3x plate reader (Molecular Devices, San Jose, CA, USA) set to 37 °C, an excitation wavelength of 485 nm, and an emission wavelength of 520 nm. The fluorescence intensities were measured every 2 min for 1 h.

The absorbance values from the ORAC assay were processed using Equation (4) to determine the radical scavenging ability of each compound.

(4) $$Net\;AUC=0.5+\sum_{0-29}\frac{f_{i}}{f_{0}}+\left(0.5\times \frac{f_{30}}{f_{0}}\right)$$

The parameters in Equation 4 include the following: the fluorescence intensity at readings 0 to 29 (*f_i_*), the initial fluorescence intensity at reading 0 (*f_0_*), and the fluorescence intensity at reading 30 (*f_30_*). The percent radical scavenging was determined using Equation (5), where net *AUC_t_* was the area under the curve for the test compound, net *AUC_c_* was the area under the curve for the control wells, and net *AUC_fmax_* was the area under the curve for the maximum fluorescence wells where no AAPH was added, which is also referred to as the positive control.

(5) $$Percent\;Radical\;Scavenging=\frac{\left(Net\;{AUC}_{t}-Net\;{AUC}_{c}\right)}{\left(Net\;{AUC}_{f_{max}}-Net\;{AUC}_{c}\right)}\times 100$$

OriginPro Version 2023b (10.05) was used for the nonlinear regression line fitting of the percent radical scavenging values utilizing Equation 1. For some compounds, Origin was unable to generate proper dose–response curves as either the generated curves had parameters that were unrealistic for the plotted data points (i.e., a top asymptote value of 854% ± 2501%) or the software gave an error message that the fitting failed. Origin recommended Hill slope values (4.15481 for DPPH; 4.15241 for ABTS; and 1.66083 for ORAC) in the “Parameters” tab when setting up the “DoseResp” function that can be kept constant. As a result, this feature was utilized for those compounds to enable Origin to produce better fitted dose–response curves.

### 4.4. Cell Culture Studies

The human trophoblast HTR8/SVneo cells (ATCC) were cultured in RPMI supplemented with 5% fetal bovine serum and 1% penicillin–streptomycin in a humidified incubator containing 5% CO_2_ at 37 °C. Cells in the logarithmic growth phase were used in subsequent experimentation. Compounds **2**, **3**, **5**, and **6** were dissolved in DMSO and stored at 4 °C. The final concentration of DMSO in the medium was kept at less than 0.1%, which has been shown to be nonlethal to the cells. Cells in the H_2_O_2_-treated group were treated with 100 μM H_2_O_2_ alone. Cells in the antioxidant + H_2_O_2_-treated groups were pre-treated with 0.1–50 μM of antioxidant for 30 min, respectively, and then treated with 100 μM H_2_O_2_ for 18 h. At the end of the experiments, the culture supernatants were collected and stored at −20 °C until they were assayed.

### 4.5. Cell Viability Assay

Cell viability was determined following treatment with antioxidant and H_2_O_2_, with a Cell Counting Kit-8 assay. HTR8 cells were cultured in 48-well culture plates for 24 h. The cells were treated with different concentrations of the antioxidant and H_2_O_2_ for 18 h. Then, a total of 10 μL CCK-8 reagent was added to each well and incubated for 1 h in a 5% CO_2_ incubator at 37 °C. Finally, the optical density values were acquired with a microplate reader at 450 nm.

### 4.6. Mitochondrial ROS Measurements

HTR8 cells were seeded in 48-well plates (Nalgene Nunc International) and incubated at 37 °C in a 10% CO_2_ humidified incubator overnight. Next day, cells were subjected to H_2_O_2_ treatment for 18 h, along with various concentrations of **2**, **3**, **5**, and **6**. After 18 h, the cells were loaded with MitoSOX Red fluorogenic dye at 25 nM final concentrations, respectively, for 15 min. The cells were washed three times with PBS and the specific fluorescence of the dyes was visualized and photographed using an inverted EVOS^®^ FL Imaging System (Advanced Microscopy Group). Morphometric measurements were performed using ImageJ software version 1.47 (National Institute of Health (NIH), Bethesda, MD, USA; https://imagej.net/ij/, accessed on 8 August 2024).

### 4.7. Enzyme-Linked Immunosorbent Assay (ELISA)

Soluble FLT-1 (sFLT-1) in the culture supernatants were measured by ELISA using the human VEGF receptor 1 (VEGFR1) Quantikine kit (R&D Systems) following the manufacturer’s instructions. This assay has an intra-assay coefficient of variation of 2.6–3.8% and an inter-assay coefficient of variation of 5.5–9.8%.

### 4.8. Computational Methods

The electronic structure of the studied hydrazones was predicted based on density functional theory (DFT). The initial conformation ensemble was simulated using the Global Optimizer Algorithm (GOAT) offered by the ORCA 6.0 suite [40,41,42,43]. The resulting geometries with lowest energy were subjected to geometry optimization and frequency calculations. All calculations were performed in the Gaussian 16.0 program suite at the M06-2X level and 6-311+G(d,p) basis set [44,45]. The Fukui functions were calculated by Multiwfn at the same level of theory [46].

To calculate the EDDB(r) descriptor, charge and bond order matrix calculations were conducted using NBO 7.0 software [47,48]. The EDDB analysis was performed using the code RunEDDB [49,50].

## 5. Conclusions

In conclusion, the results showed that the synthesized diaryl hydrazones, specifically **2**, **3**, **5**, and **6**, exhibited promising antioxidant capabilities, as their radical scavenging activities surpassed those of reference compounds, Trolox and ascorbic acid. Pre-treatment with **2**, **3**, **5**, and **6** reduced sFLT-1 production in H_2_O_2_-exposed trophoblast cells. All four antioxidants decreased sFLT-1 production at a systematic rate over a range of concentrations, which suggested that they could be used to treat various cases of oxidative stress. Further analysis showed that pre-treatment with the compounds decreased the production of mitochondrial ROS in H_2_O_2_-exposed trophoblast cells. This suggests that the key factor in the development of PE, oxidative stress, and downstream signaling pathways can be diminished by these antioxidants. Compound **6** appeared to restore the sFLT-1 level to that of healthy cells in the lowest concentration. The theoretical calculations confirmed that the electronic structure of the compounds are nearly identical, thus providing an explanation to their highly similar performance in a range of assays.

## Figures and Tables

**Table 1:** List of the synthesized diaryl hydrazones used in this study.

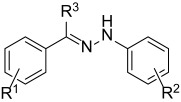			
Compound	R^1^	R^2^	R^3^
**1**	2,3,4,5,6-penta-F	4-CF_3_	H
**2**	4-N(CH_3_)_2_	4-CF_3_	H
**3**	2-OH, 4-OCH_3_	4-CF_3_	H
**4**	3,4-di-OCH_3_	3-CF_3_	H
**5**	4-N(CH_3_)_2_	3-CF_3_	H
**6**	4-N(CH_3_)_2_	2-CF_3_	H
**7**	4-NO_2_	3-CF_3_	H
**8**	4-CF_3_	2,4-NO_2_	Ph
**9**	4-(CH_2_=NNH-(3′-CF_3_C_6_H_4_)	3-CF_3_	H
**10**	2,3-(CH)_4_-5,6-(CH)_4_^a^	3-CF_3_	H

^a^ 9-anthracenyl group is attached to the C=N bond.

**Table 2:** EC_50_ summary of the hydrazones in the DPPH, ABTS, and ORAC assays.

#	EC_50_^a^ (µM) (DPPH)	E_max_ (%) (DPPH)	EC_50_^a^ (µM) (ABTS)	E_max_ (%) (ABTS)	EC_50_^a^ (µM) (ORAC)	E_max_ (%) (ORAC)
AA ^c^	26.6 ± 3.3 ^b^	89.5 ± 9.9	11.0 ± 1.1 ^b^	29.9 ± 2.7	11.4 ± 0.8 ^b^	95.8 ± 2.6
Trolox	27.2 ± 0.5	106 ± 2	10.7 ± 1.4 ^b^	38.0 ± 4.4	0.372 ± 0.021	94.2 ± 0.7
**1**	18.2 ± 0.9	102 ± 4	9.19 ± 2.30 ^b^	45.0 ± 7.7	0.213 ± 0.007	95.1 ± 0.6
**2**	13.3 ± 1.4	89.4 ± 6.9	5.80 ± 1.54 ^b^	41.2 ± 6.1	0.224 ± 0.026 ^b^	97.0 ± 1.3
**3**	17.7 ± 2.5	98.9 ± 10.7	9.98 ± 2.98 ^b^	66.3 ± 13.7	0.421 ± 0.037	75.7 ± 0.9
**4**	27.9 ± 3.6 ^b^	52.5 ± 5.8	11.7 ± 1.4 ^b^	62.2 ± 6.4	1.70 ± 0.91	115 ± 18
**5**	18.6 ± 6.0 ^b^	88.6 ± 15.3	8.79 ± 1.92 ^b^	74.0 ± 11.1	0.266 ± 0.065	85.2 ± 3.2
**6**	22.1 ± 2.7	106 ± 11	5.40 ± 1.27 ^b^	50.6 ± 7.0	0.147 ± 0.038 ^b^	81.6 ± 3.2
**7**	9.88 ± 3.18 ^b^	50.0 ± 8.5	5.99 ± 1.54 ^b^	54.1± 7.4	1.76 ± 0.13	91.7 ± 2.1
**8**	-	-	14.3 ± 3.7 ^b^	26.9 ± 7.3	18.4 ± 1.3 ^b^	71.3 ± 2.0
**9**	28.3 ± 4.8 ^b^	81.8 ± 12.0	10.2 ± 2.4 ^b^	38.4 ± 5.6	1.29 ± 0.92 ^b^	52.0 ± 7.4
**10**	16.2 ± 0.4	34.0 ± 0.6	11.6 ± 0.5	77.5 ± 2.9	0.603 ± 0.509 ^b^	32.1 ± 3.9

^a^ relative EC_50_ values, ^b^ Hill slope was kept constant, and ^c^ AA—ascorbic acid.

**Table 3:** Summary of reactivity descriptors.

	Compound 2		Compound 5		Compound 6		Compound 8		Compound 10	
	N10		N10		N10		N10		N6	
Dual descriptor ^a^	−0.091		−0.098		−0.096		−0.076		−0.068	
Mulliken charge	−0.22	0.13 ^b^	−0.24	0.10 ^b^	−0.05	0.07 ^b^	−0.23	0.02 ^b^	−0.33	0.19 ^b^
Spin density		0.52 ^b^		0.52 ^b^		0.54 ^b^		0.51 ^b^		0.47 ^b^

^a^ orbital-weighted; ^b^ radical.

**Table 4:** EDDB_H_ of the -Ph-N● fragment of compounds **2**, **5**, **6**, **8**, and **10** with dissected π and σ components.

	EDDB	EDDB_π_	EDDB_σ_
**2**	5.9366	4.5391	1.3975
**5**	5.9331	4.6330	1.3001
**6**	5.9309	4.6105	1.3204
**8**	5.9958	4.7978	1.1980
**10**	5.9357	4.6390	1.2967
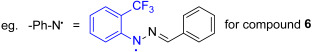			

## Data Availability

All data are included in the manuscript and the Supplementary Materials.

## Supplementary Materials

The following supporting information can be downloaded at https://www.mdpi.com/article/10.3390/molecules31010078/s1. Refs. [51,52] are cited in the Supplementary Materials.
