# Supplementary material for: Impact of Structural Features on the Antioxidant Activity of Organofluorine Diaryl Hydrazones
Source: Molecules. 2025 Dec 24;31(1):78. doi: 10.3390/molecules31010078 (PMC12786584; doi:10.3390/molecules31010078)
Supplement: Supplementary file 1 [file molecules-31-00078-s001.zip › molecules-3988346-supplementary.pdf]

SUPPORTING INFORMATION

to

Impact of Structural Features on the Antioxidant Effect of Organofluorine Diaryl Hydrazones

by Zsuzsanna K. Zsengellér [1], Maxim Mastyugin [1,2], Adrianna R. Fusco [1,2], Bernadett R. Vlocskó [2], Coryn Ferguson [1], Diana Pintye [1], Hamad Nasim [1], Saira Salahuddin [3], Brett C. Young [4], Béla Török [2] *, and Marianna Török [2,]*

1 Department of Chemistry, University of Massachusetts Boston, 100 Morrissey Blvd, Boston, MA, US

2 Department of Medicine, Beth Israel Deaconess Medical Center, Boston, Harvard Medical School Boston, MA 02215, USA

3 Department of OB/GYN, Beth Israel Lahey Health, Boston MA, USA

4 Department of OB/GYN, Mt Auburn Hospital, Boston MA, USA

** Correspondence: marianna.torok@umb.edu and bela.torok@umb.edu*

1. Chemistry:

1.1. General Information

The derivatives of aldehydes and hydrazine used, along with NMR reference standards and solvents ($CDCl_3$ and DMSO-d_6, 99.8%), were obtained from Millipore Sigma and Cambridge Isotope Laboratories and used without further purification. Additional reagents and materials were acquired from Fisher Scientific and Oakwood Chemical.

The mass spectrometric analysis and purity determination of the products were performed using an Agilent 6850 gas chromatograph coupled to a 5973 mass spectrometer, operated in electron impact (EI) ionization mode at 70 eV with a 30 m DB-5 column (J&W Scientific). Additional HRMS data were collected using an Agilent 7250 GC-QTOF mass spectrometer under identical EI conditions.

^{1}H, ^{13}C, and ^{19}F NMR spectra were recorded on a 400 MHz Agilent MMR400DD2 NMR spectrometer using DMSO-d_6 and $CDCl_3$ as solvents. Tetramethylsilane (TMS) or residual solvent peaks served as internal references, with chemical shifts (δ) reported in part per million (ppm). Signal multiplicities in ^{1}H NMR spectra are designated as s (singlet), d (doublet), t (triplet), q (quartet), and m (multiplet). All measurements were conducted at 25 +/- 1 °C. All products were known compounds, and their NMR spectra are in agreement with previously published results[1].

1.2. Spectral data of the compounds:

1-((perfluorophenyl)methylene)-2-(4-(trifluoromethyl)phenyl)hydrazine (1)

^{1}H NMR (399.822 MHz, $CDCl_3$) δ (ppm) = 8.08 (s, 1H), 7.74 (s, 1H), 7. 55-7.52 (d, 2H, J=8 Hz), 7.17-7.14 (d, 2H, J=12 Hz); ^{13}C NMR (DMSO, 100 MHz) δ (ppm) = 150.84, 143.46, 139.16, 139.02, 138.19, 136.58, 135.77, 134.31, 131.90, 127.32, 122.39, 121.98-121.88 (m), 111.90, 39.83; ^{19}F NMR (376 MHz, $CDCl_3$) δ (ppm): -61.39, -142.91-142.99, -154.41-154.53, -162.37-162.49; HRMS (EI): m/z: [M+] calcd. for $C_{14}H_6F_8N_2$: 354.0403; found: 354.0409.

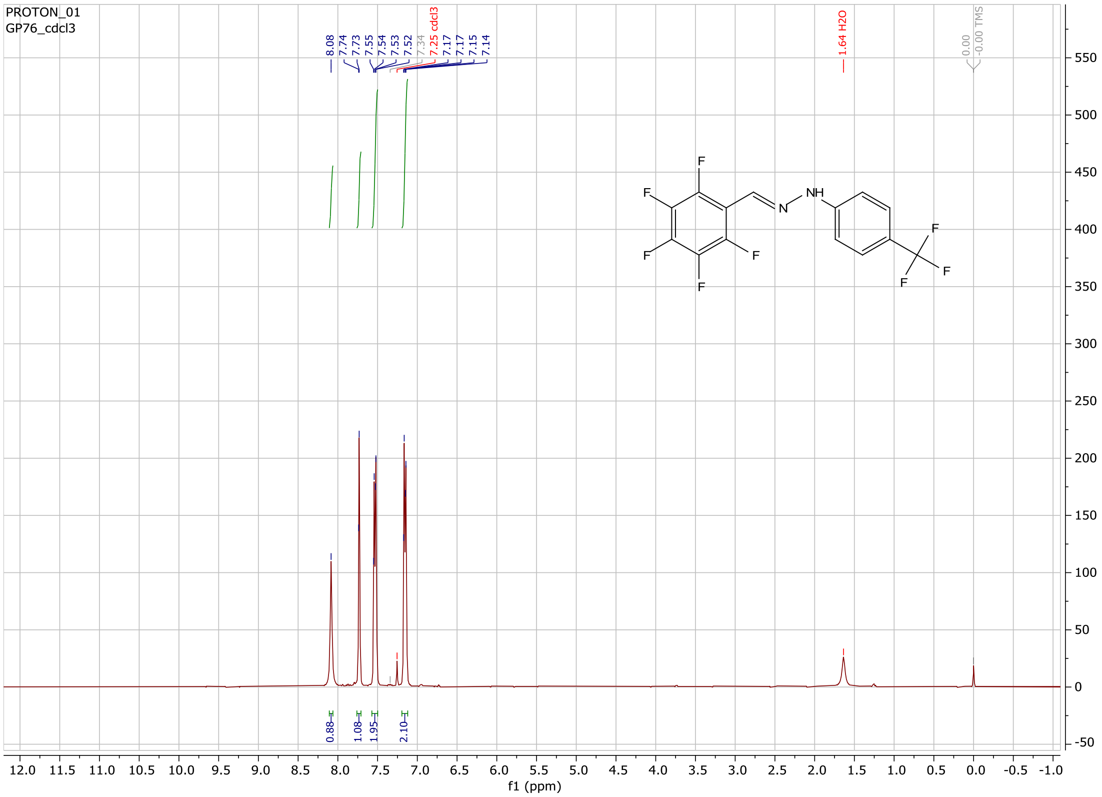

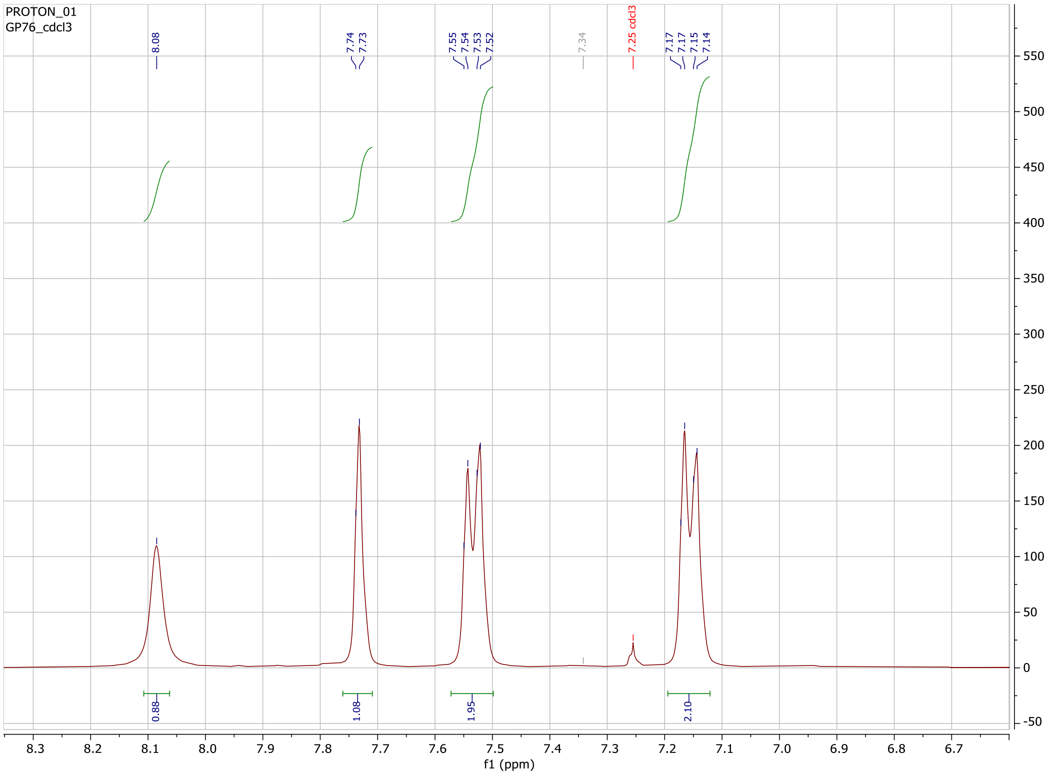

Figure S1. ^{1}H NMR of 1-((perfluorophenyl)methylene)-2-(4-(trifluoromethyl)phenyl)hydrazine (**1**).

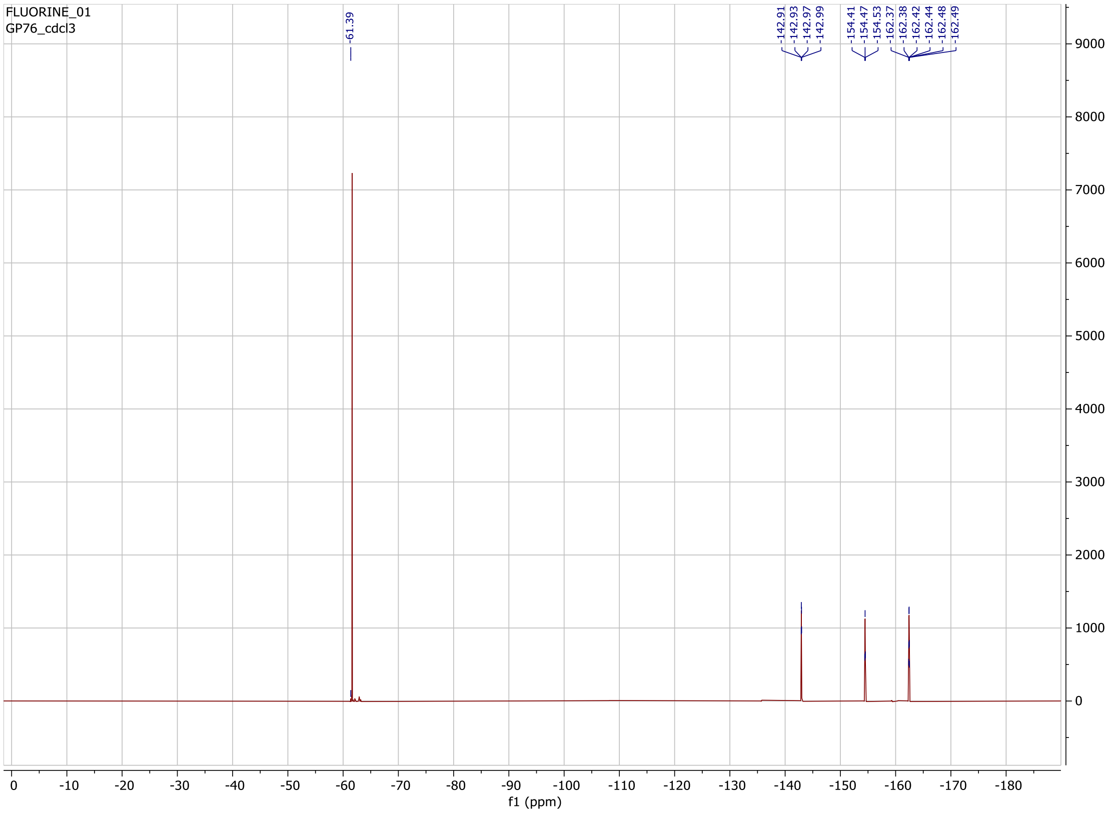

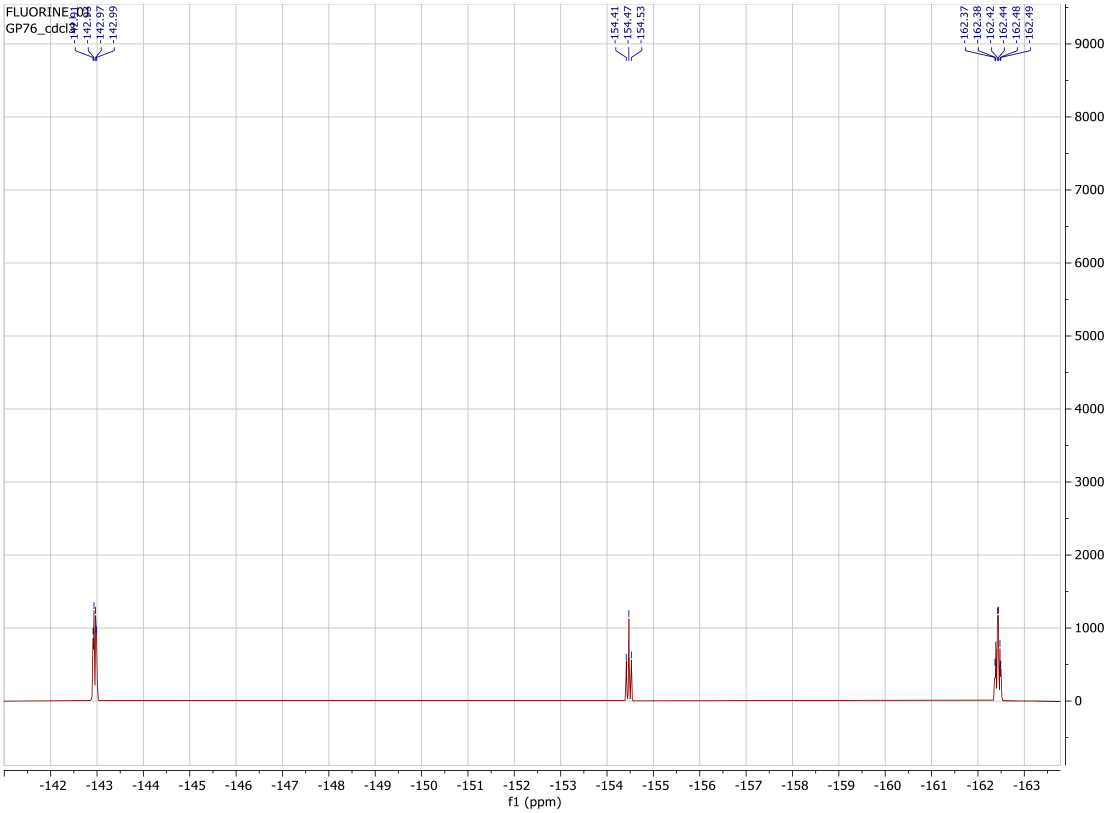

Figure S2. ^{19}F NMR of 1-((perfluorophenyl)methylene)-2-(4-(trifluoromethyl)phenyl) hydrazine (**1**).

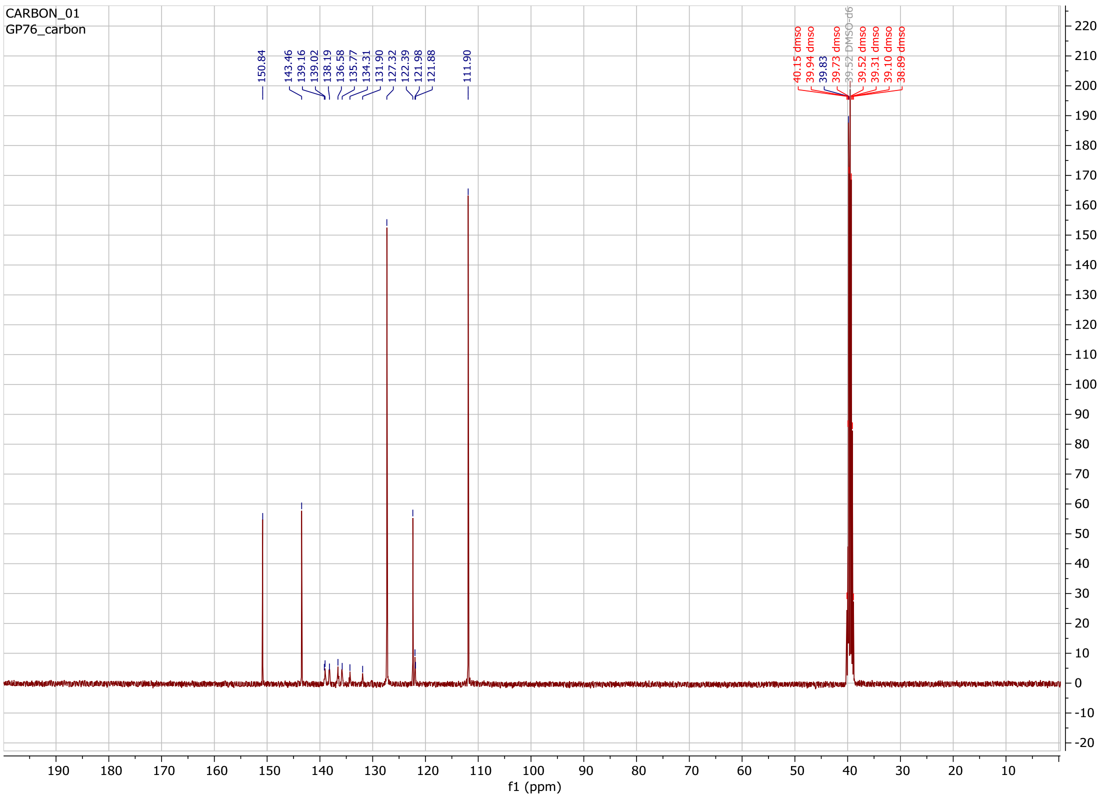

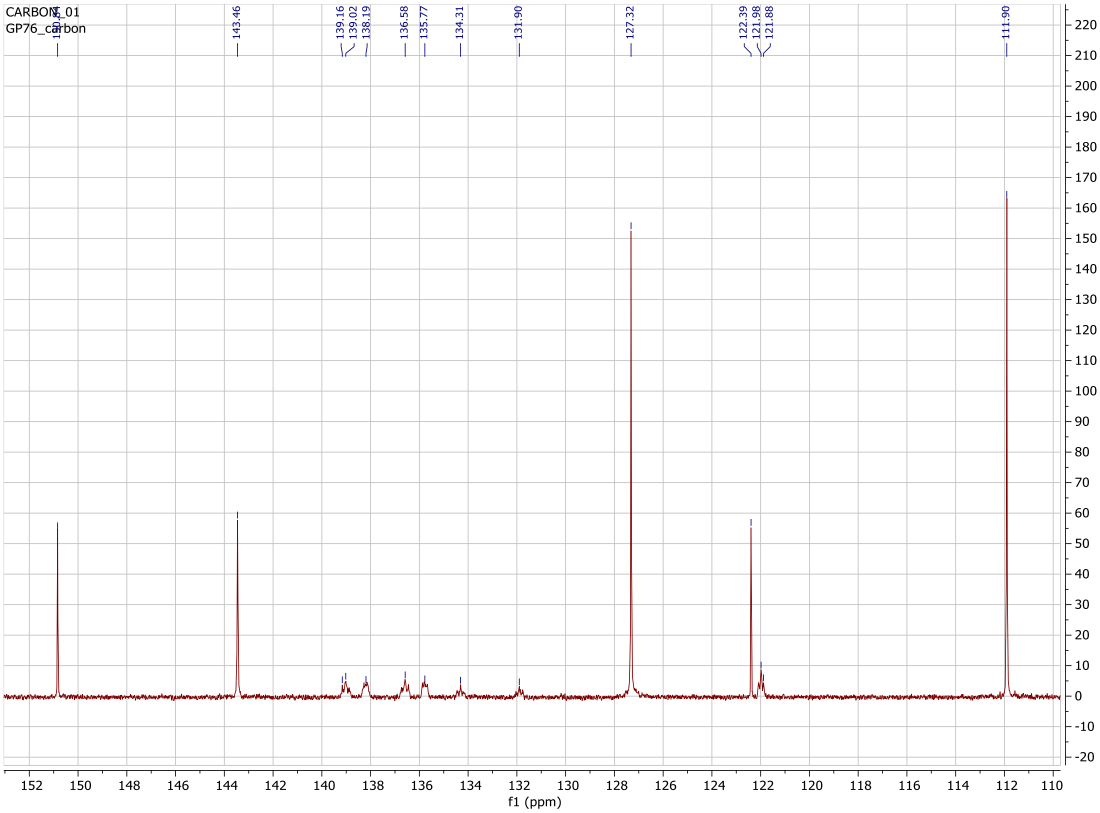

Figure S3. ^{13}C NMR of 1-((perfluorophenyl)methylene)-2-(4-(trifluoromethyl)phenyl) hydrazine (**1**).

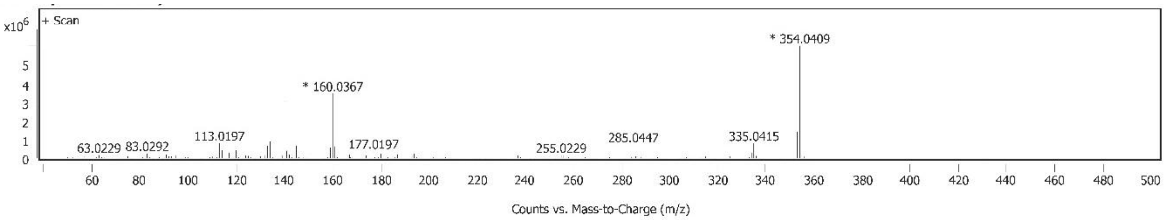

Figure S4. HRMS of 1-((perfluorophenyl)methylene)-2-(4-(trifluoromethyl)phenyl)hydrazine (**1**).

***N,N*-dimethyl-4-((2-(4-(trifluoromethyl)phenyl)hydrazineylidene)methyl)aniline (2)**

^{1}H NMR (399.822 MHz, DMSO) δ (ppm) = 10.46 (s, 1H), 7.85 (s, 1H), 7.52 – 7.49 (m, 4H), 7.15 – 7.13 (d, 2H), 6.73 – 6.71 (d, 2H), 2.92 (s, 6H); ^{13}C NMR (DMSO, 100 MHz) δ (ppm) = 150.74, 148.77, 140.30, 129.32 – 121.25 (q, J = 269 Hz), 127.39, 126.55 – 126.44 (q, J = 4 Hz), 123.00, 118.07 – 117.12 (q, J = 32 Hz), 112.08, 111.27, 39.93; ^{19}F NMR (376 MHz, DMSO) δ (ppm): -59.21; HRMS (EI): m/z: [M+] calcd. for $C_{16}H_{16}F_3N_3$: 307.1296; found: 307.1297.

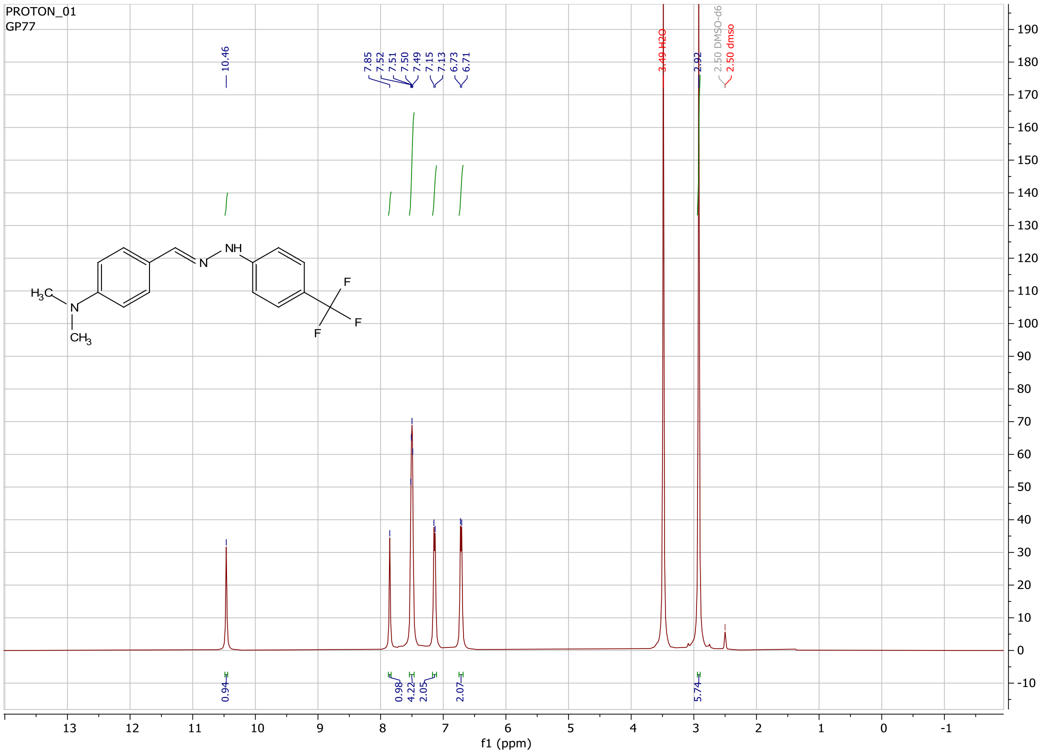

Figure S5. ^{1}H NMR of *N,N*-dimethyl-4-((2-(4-(trifluoromethyl)phenyl)hydrazineylidene) methyl)aniline (**2**).

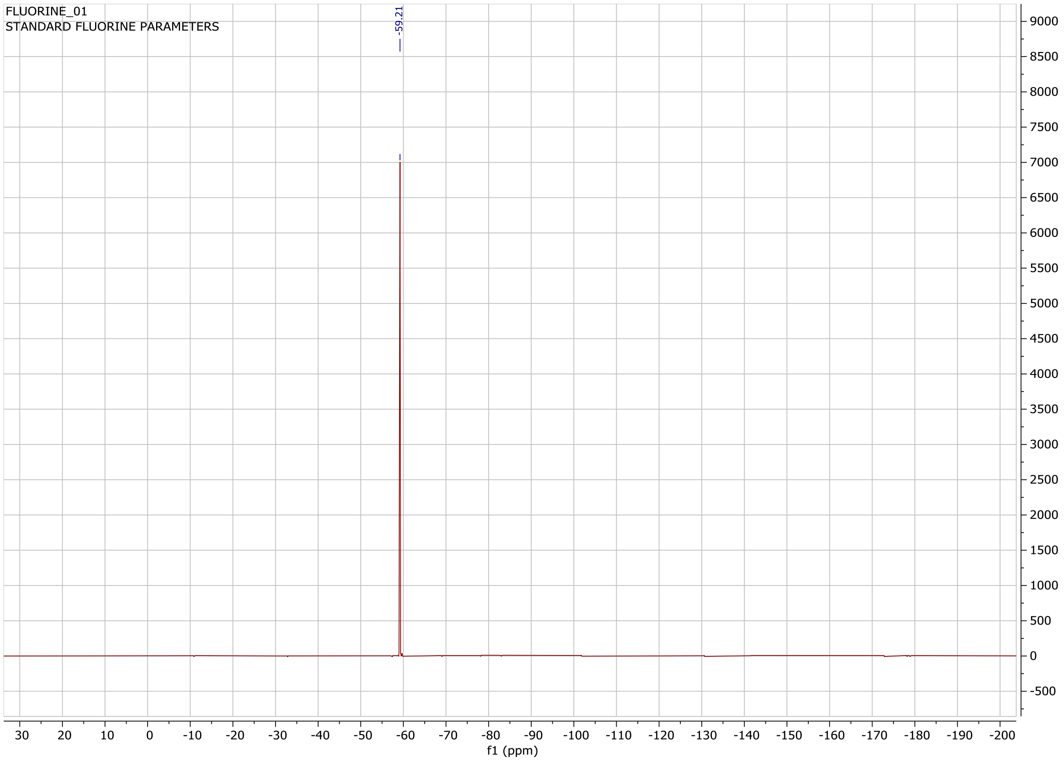

Figure S6. ^{19}F NMR of *N,N*-dimethyl-4-((2-(4-(trifluoromethyl)phenyl)hydrazineylidene) methyl)aniline (**2**).

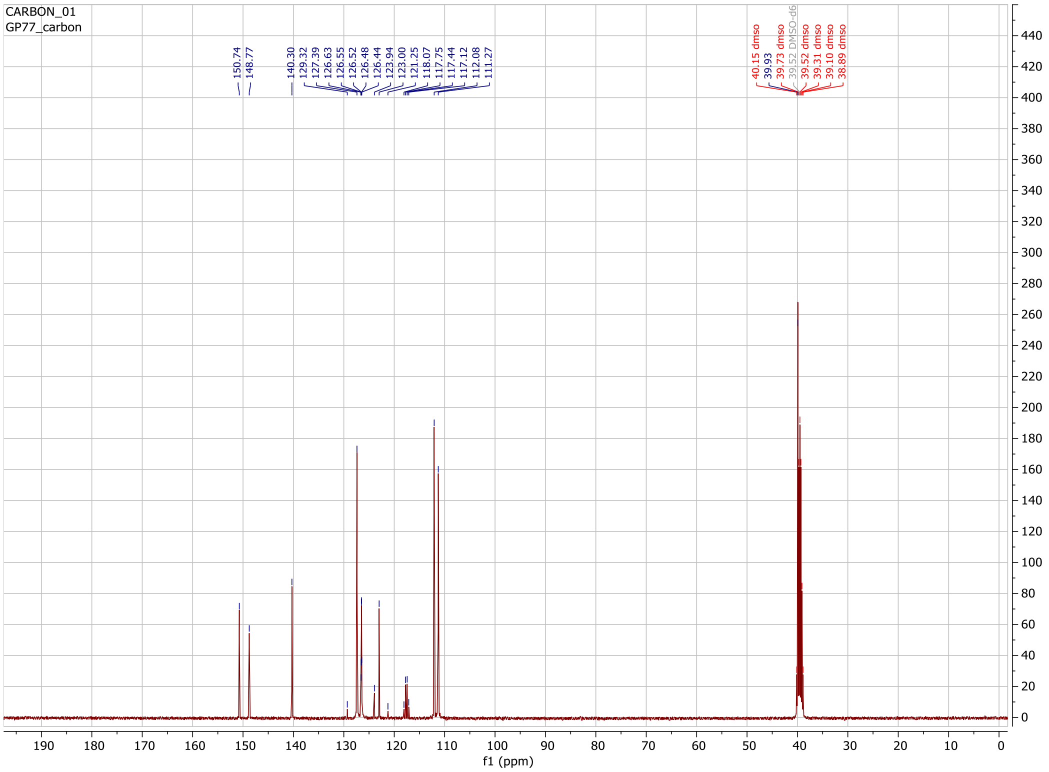

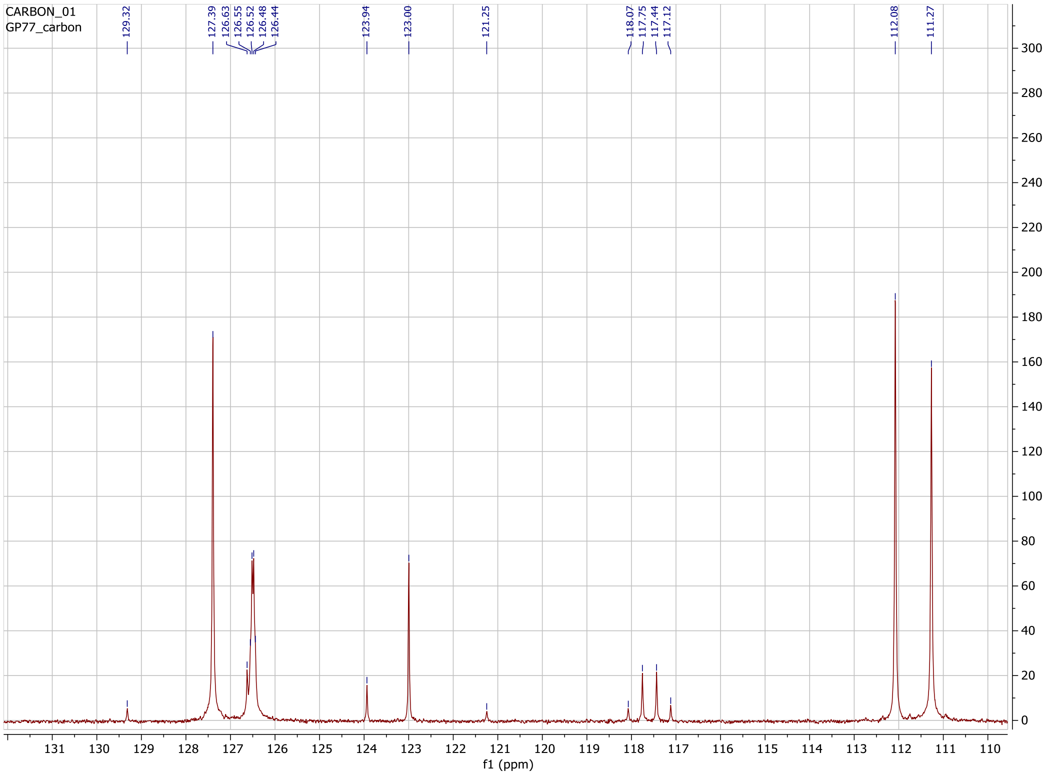

Figure S7. ^{13}C NMR of *N*,*N*-dimethyl-4-((2-(4-(trifluoromethyl)phenyl)hydrazineylidene) methyl)aniline (**2**).

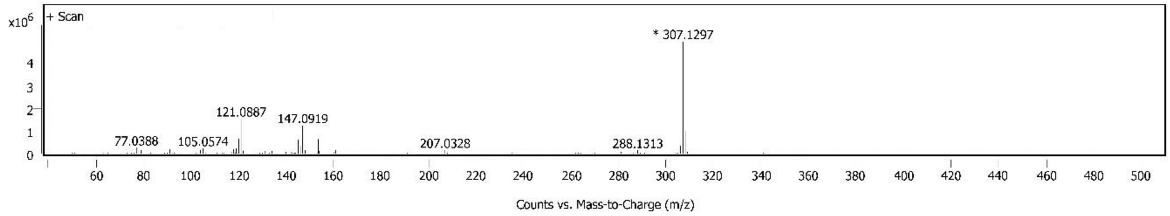

Figure S8. HRMS of *N,N*-dimethyl-4-((2-(4-(trifluoromethyl)phenyl)hydrazineylidene) methyl)-aniline (**2**).

5-methoxy-2-((2-(4-(trifluoromethyl)phenyl)hydrazineylidene)methyl)phenol (3)

^{1}H NMR (399.822 MHz, DMSO) δ (ppm) = 10.64 (s, 1H), 10.50 (s, 1H), 8.17 (s, 1H), 7.54 – 7.50 (m, 3H), 7.07 – 7.05 (d, 2H), 6.51 – 6.47 (m, 2H), 3.75 (s, 3H); ^{13}C NMR (DMSO, 100 MHz) δ (ppm) = 161.09, 157.54, 148.07, 139.60, 129.20 – 121.13 (q, *J* = 269 Hz), 128.65, 126.77 – 126.65 (q, *J* = 4 Hz), 118.73 – 117.78 (q, *J* = 32 Hz), 113.53, 111.27, 106.34, 101.15, 55.24; ^{19}F NMR (376 MHz, DMSO) δ (ppm): -59.14; HRMS (EI): m/z: [M+] calcd. for $C_{15}H_{13}F_3N_2O_2$: 310.0929; found: 310.0926.

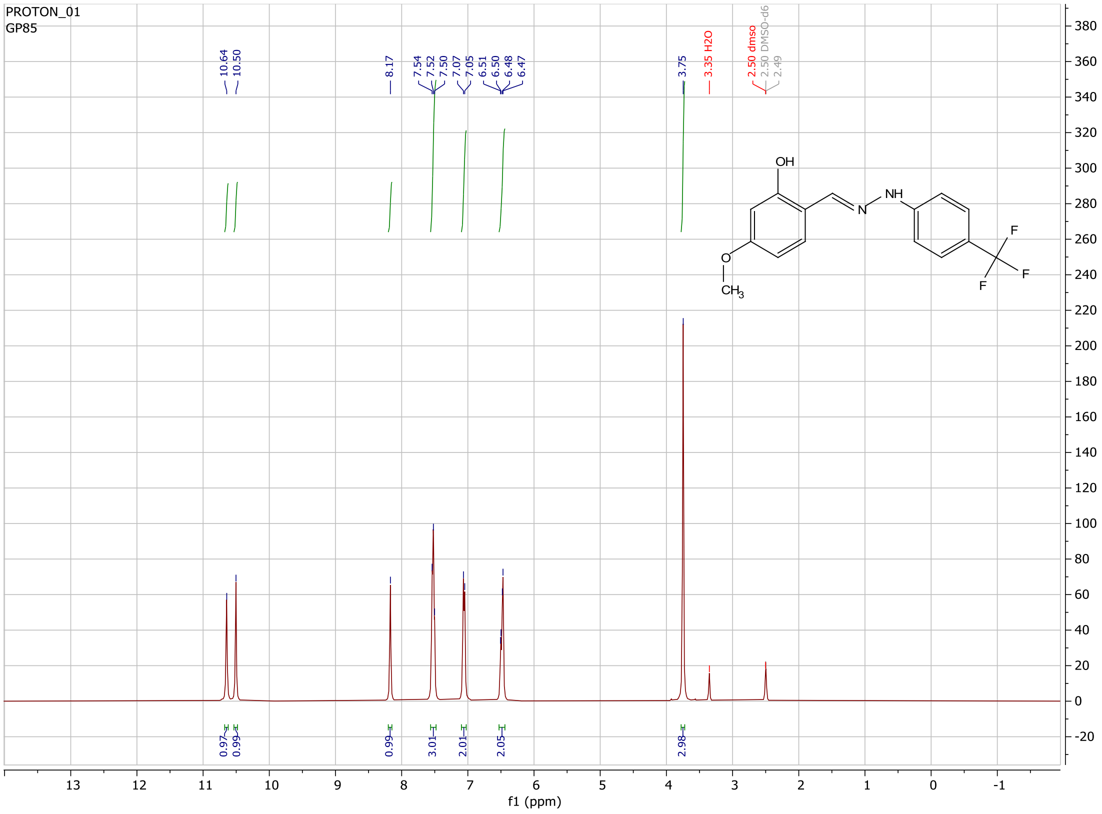

Figure S9. ^{1}H NMR of 5-methoxy-2-((2-(4-(trifluoromethyl)phenyl)hydrazineylidene) methyl)phenol (**3**).

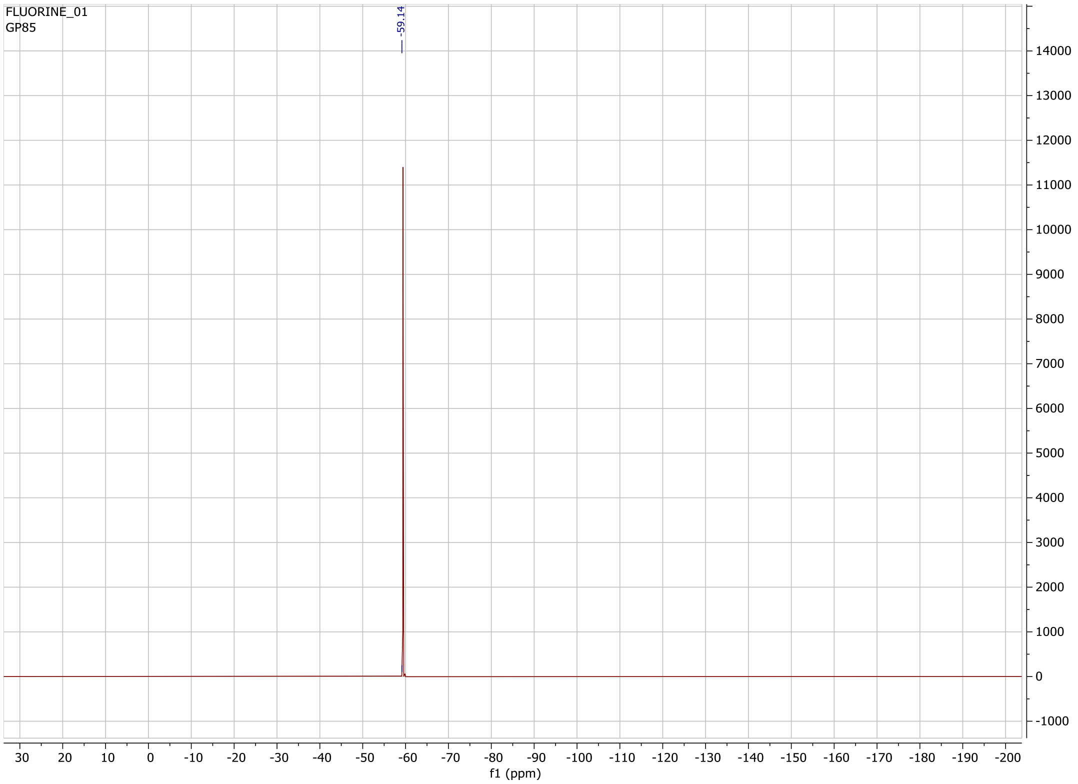

Figure S10. ^{19}F NMR of 5-methoxy-2-((2-(4-(trifluoromethyl)phenyl)hydrazineylidene) methyl)phenol (**3**).

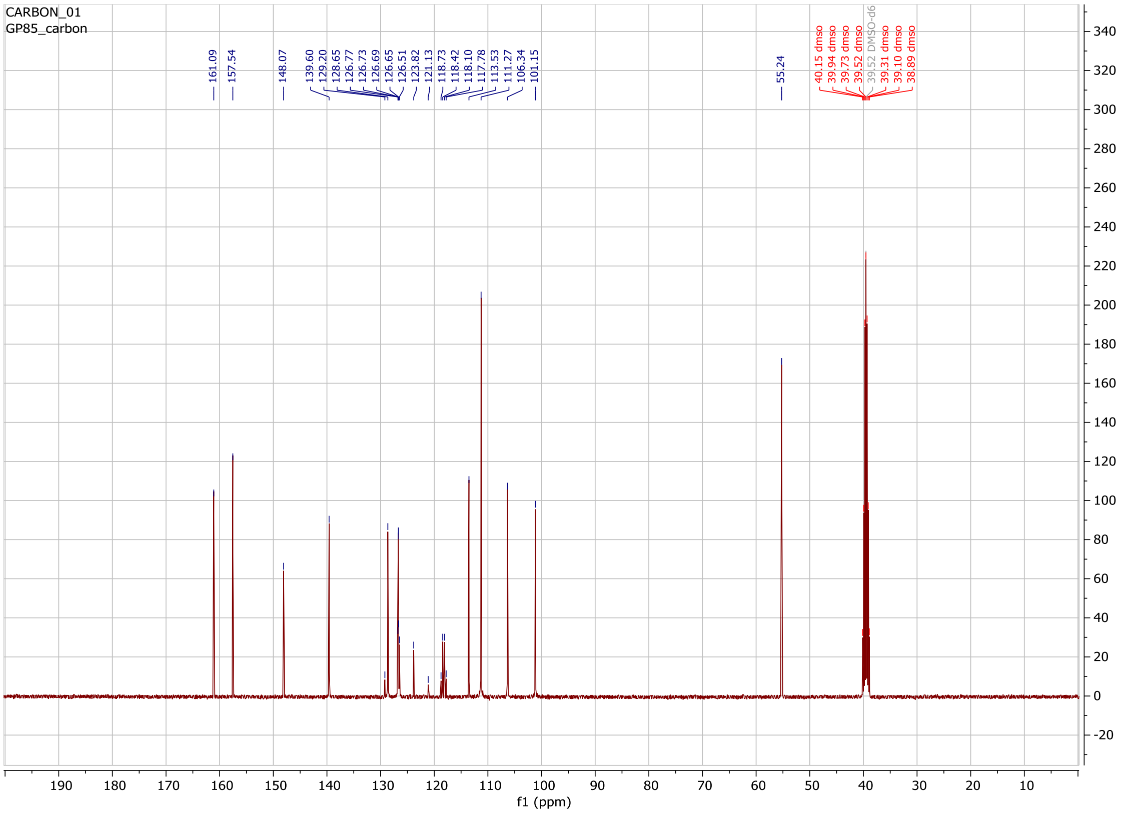

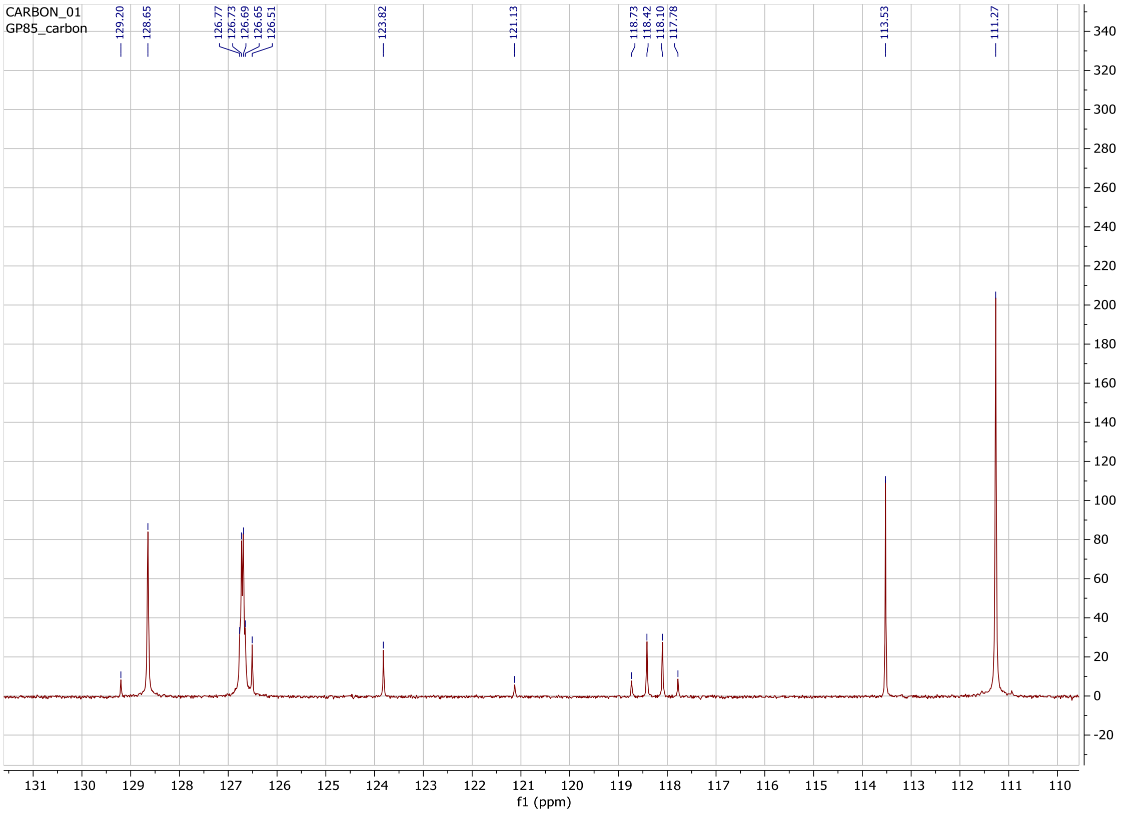

Figure S11. ^{13}C NMR of 5-methoxy-2-((2-(4-(trifluoromethyl)phenyl)hydrazineylidene) methyl)phenol (**3**).

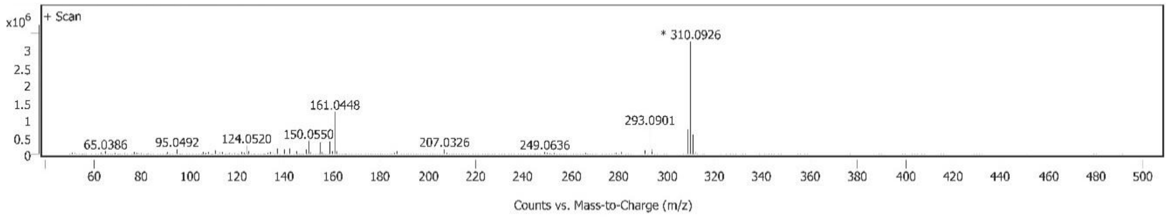

Figure S12. HRMS of 5-methoxy-2-((2-(4-(trifluoromethyl)phenyl)hydrazineylidene) methyl)phenol (**3**).

1-(3,4-dimethoxybenzylidene)-2-(3-(trifluoromethyl)phenyl)hydrazine (4)

^{1}H NMR (399.822 MHz, DMSO) δ (ppm) = 10.75 (s, 1H), 8.18 (s, 1H), 7.50 – 7.40 (m, 2H), 7.32 (s, 1H), 7.29 – 7.27 (d, 1H), 7.12 – 6.99 (m, 3H), 3.81 (s, 3H), 3.78 (s, 3H); ^{13}C NMR (DMSO, 100 MHz) δ (ppm) = 152.65, 146.71, 145.82, 133.79, 130.14, 130.49 – 129.56 (q, J = 32 Hz), 128.65, 128.40 – 120.27 (q, J = 271 Hz), 116.52, 115.57, 114.64-114.60 (q, J = 4 Hz), 112.62, 107.63 – 107.59 (q, J = 4 Hz), 60.85, 55.65; ^{19}F NMR (376 MHz, DMSO) δ (ppm): -61.27; HRMS (EI): m/z: [M+] calcd. for $C_{16}H_{15}F_3N_2O_2$: 324.1086; found: 324.1081.

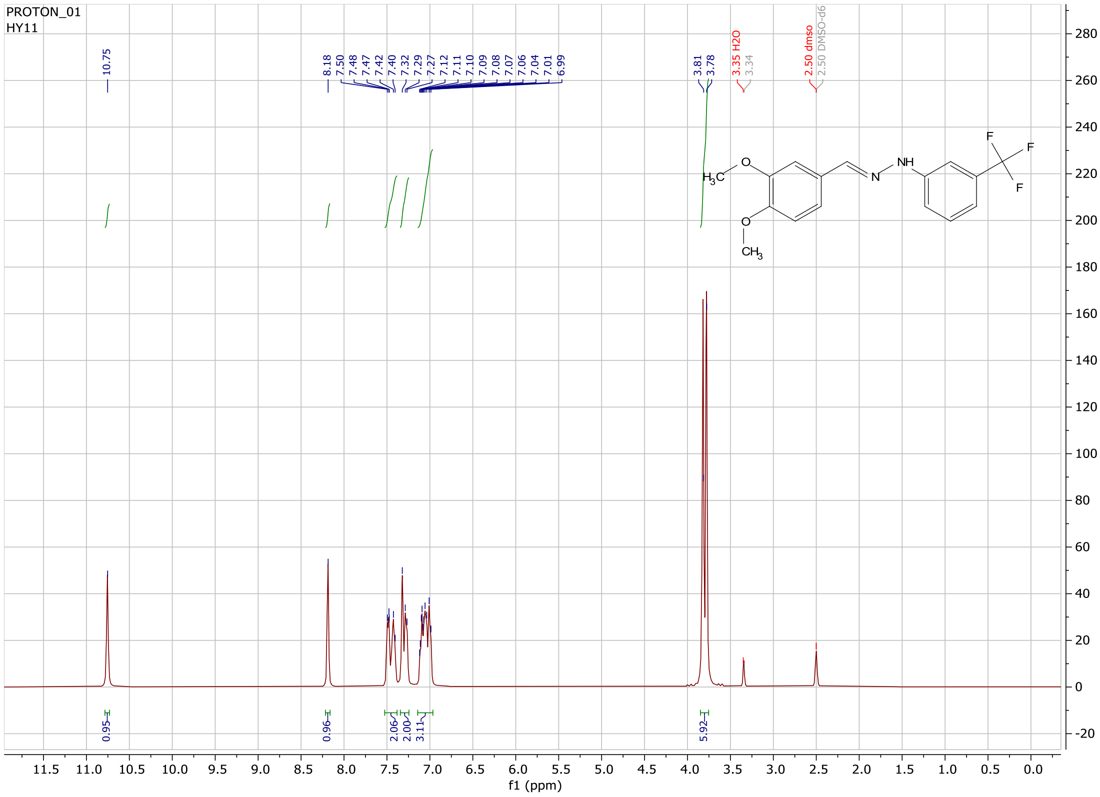

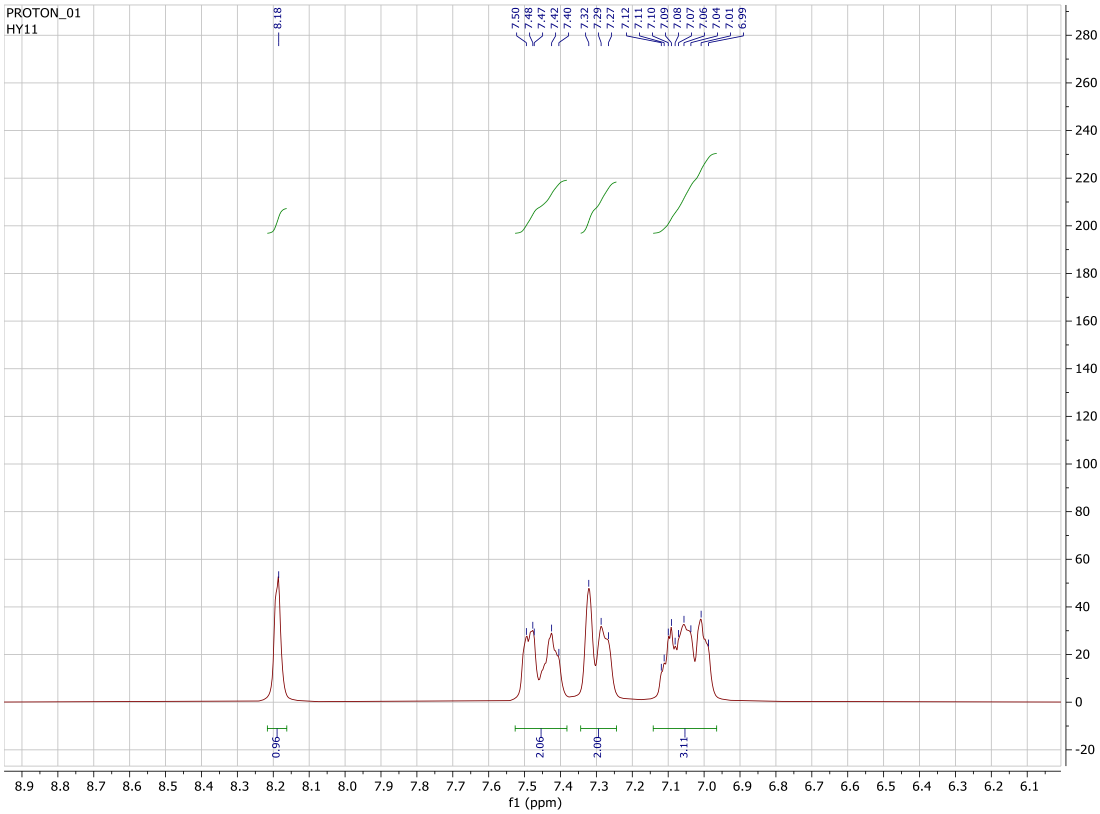

Figure S13. ^{1}H NMR of 1-(3,4-dimethoxybenzylidene)-2-(3-(trifluoromethyl)phenyl)hydrazine (**4**).

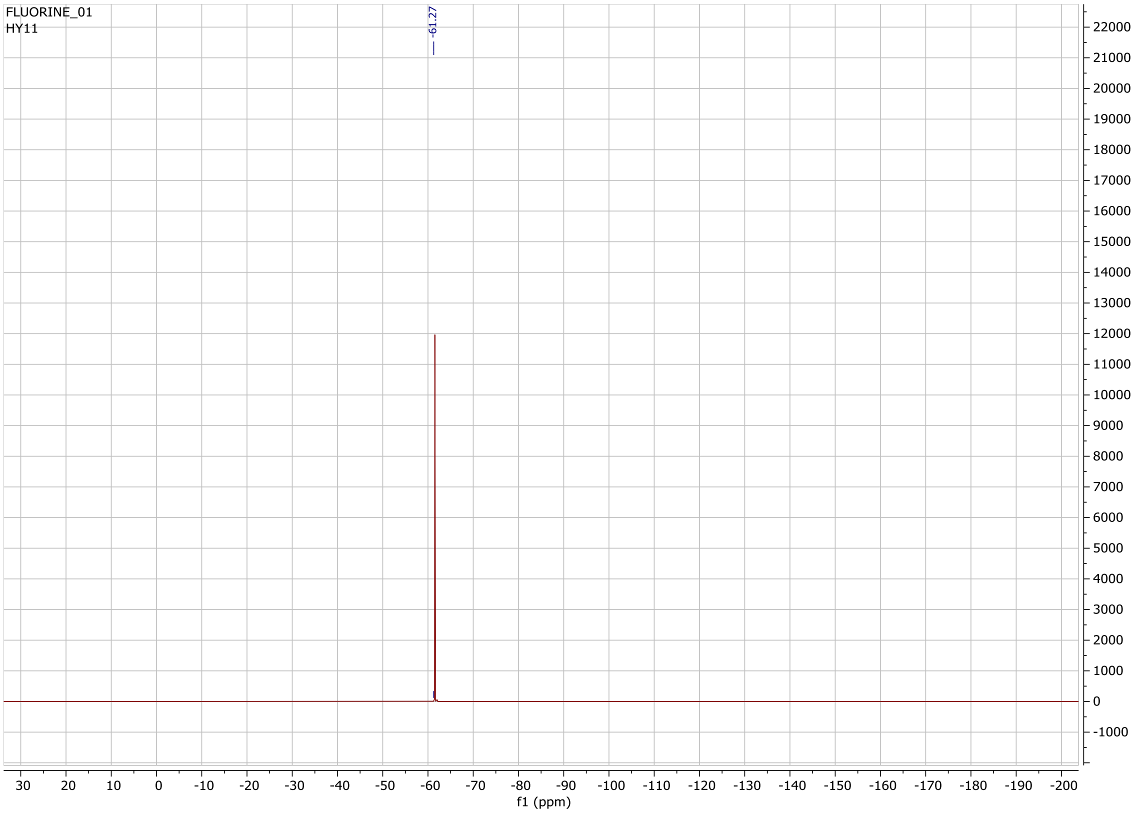

Figure S14. ^{19}F NMR of 1-(3,4-dimethoxybenzylidene)-2-(3-(trifluoromethyl)phenyl)hydrazine (**4**).

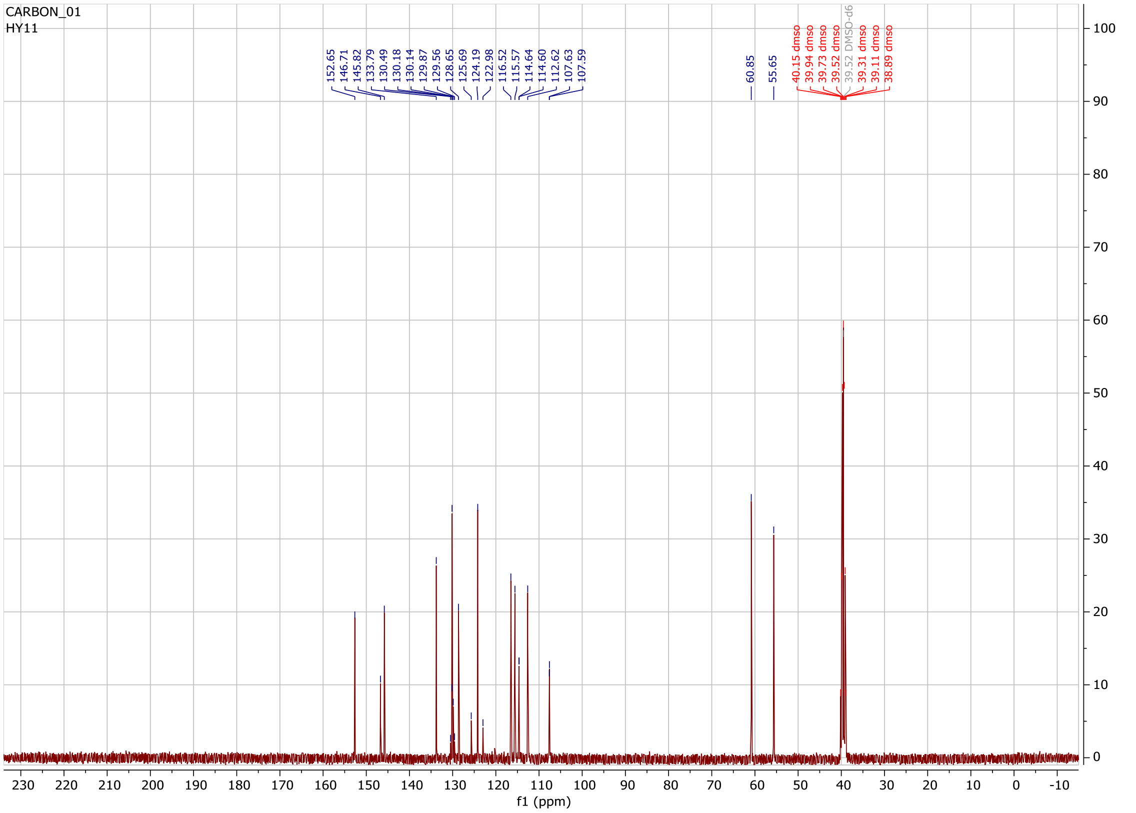

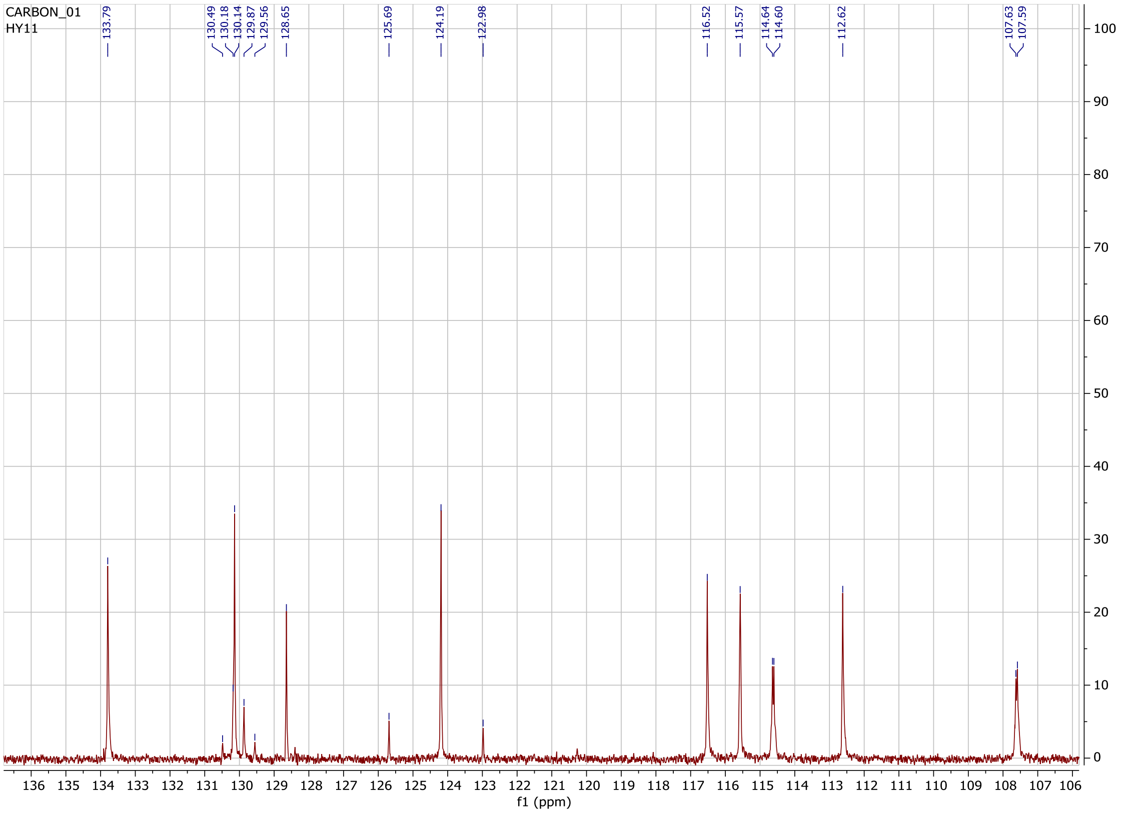

Figure S15. ^{13}C NMR of 1-(3,4-dimethoxybenzylidene)-2-(3-(trifluoromethyl)phenyl)hydrazine (**4**).

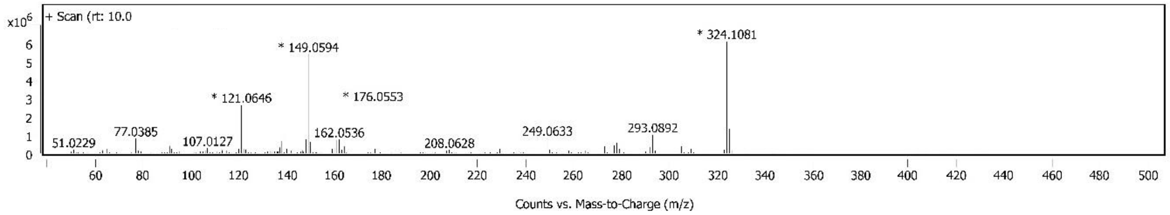

Figure S16. HRMS of 1-(3,4-dimethoxybenzylidene)-2-(3-(trifluoromethyl)phenyl)hydrazine (**4**).

***N,N*-dimethyl-4-((2-(3-(trifluoromethyl)phenyl)hydrazineylidene)methyl)aniline (5)**

^{1}H NMR (399.822 MHz, DMSO) δ (ppm) = 10.45 (s, 1H), 7.83 (s, 1H), 7.51 - 7.48 (d, 4H), 7.12 – 7.10 (d, 2H), 6.74 – 6.72 (d, 2H), 2.95 (s, 6H); ^{13}C NMR (DMSO, 100 MHz) δ (ppm) = 150.67, 146.47, 139.68, 130.55-129.62 (q, *J*= 32 Hz), 130.16, 128.65-120.52 (q, *J*= 272 Hz), 127.28, 123.10, 115.31, 113.86 -113.83 (q, *J*= 4 Hz), 112.11, 107.42 – 107.38 (q, *J*= 4 Hz), 39.93; ^{19}F NMR (376 MHz, DMSO) δ (ppm): -62.35; HRMS (EI): m/z: [M+] calcd. for $C_{16}H_{16}F_3N_3$: 307.1226; found: 307.1292.

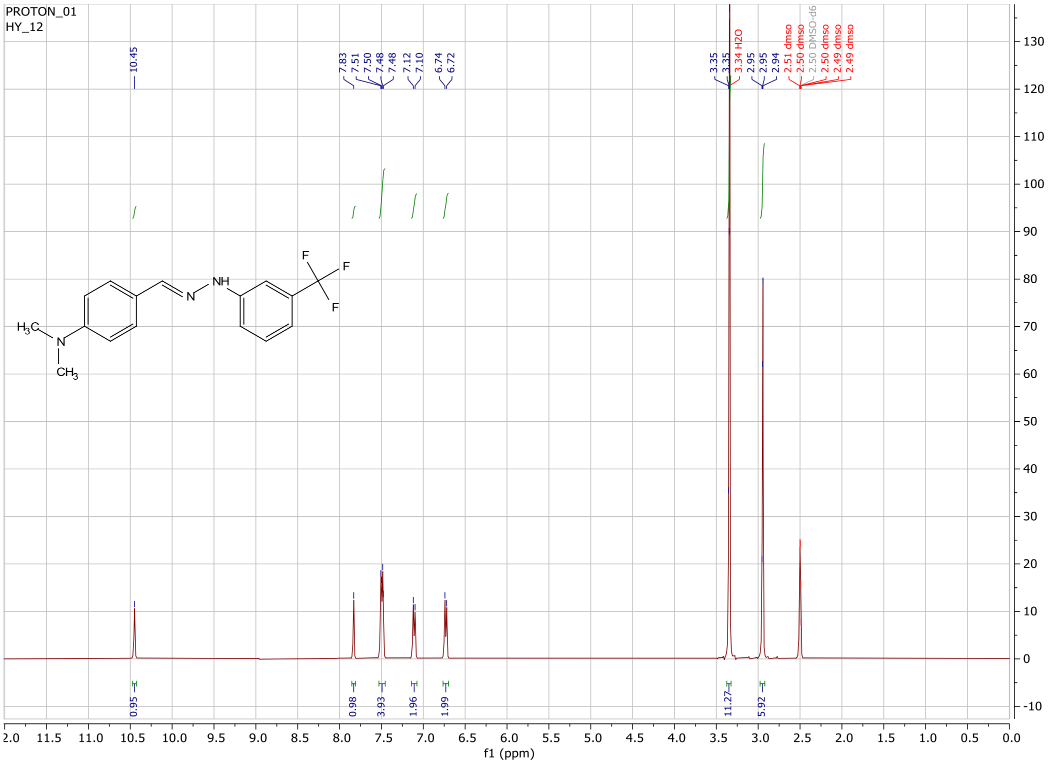

Figure S17. ^{1}H NMR of N,N-dimethyl-4-((2-(3-(trifluoromethyl)phenyl)hydrazineylidene)-methyl)aniline (**5**).

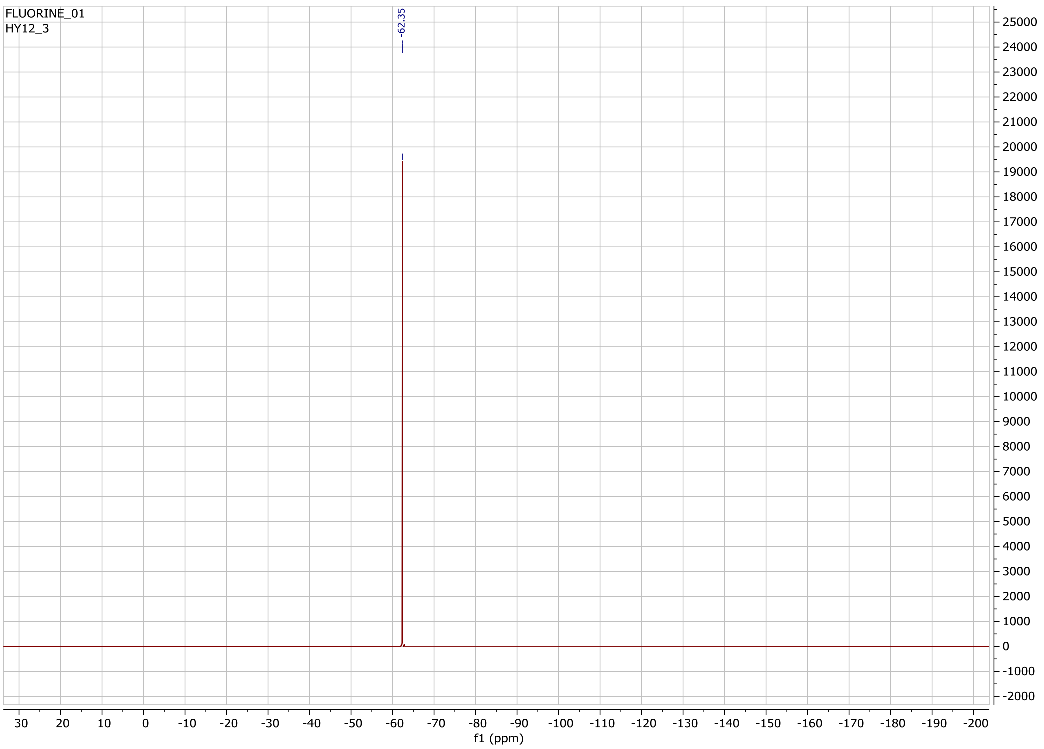

Figure S18. ^{19}F NMR of N,N-dimethyl-4-((2-(3-(trifluoromethyl)phenyl)hydrazineylidene)-methyl)aniline (**5**).

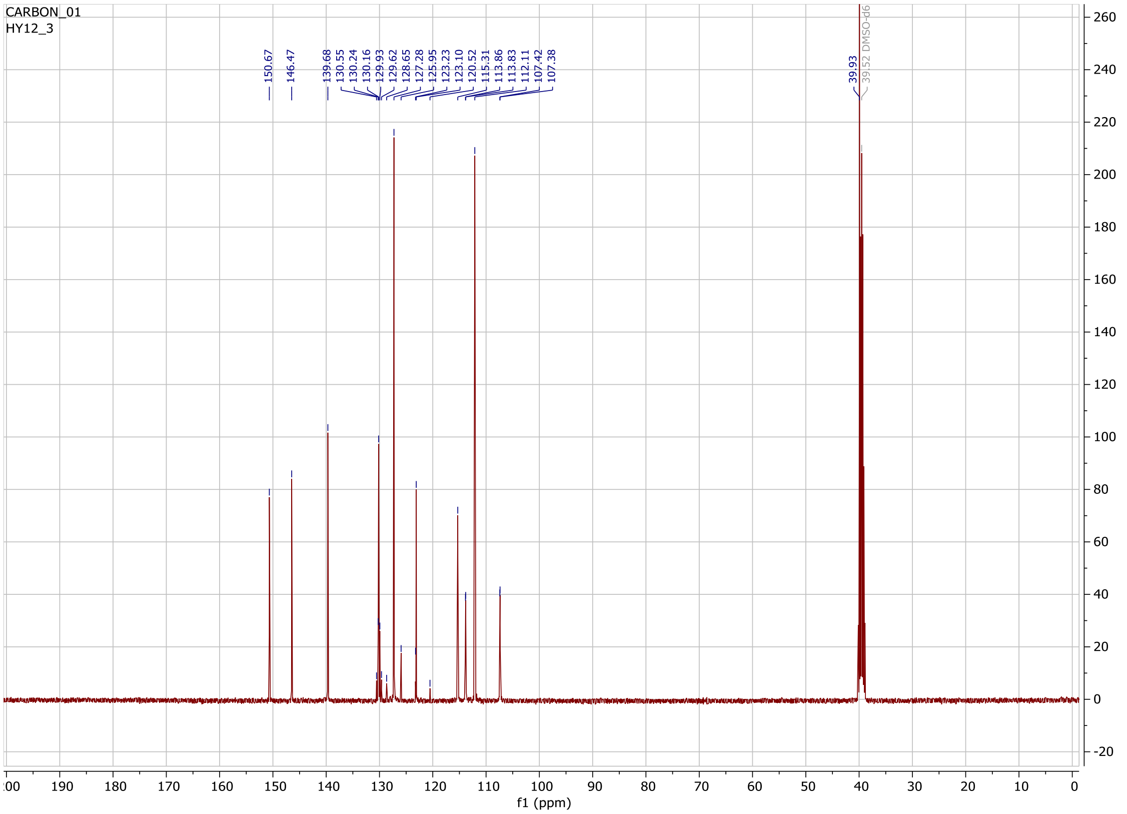
CARBON_01
HY12_3
150.67
146.47
139.68
130.55
130.24
130.16
129.93
129.62
128.65
127.28
125.95
123.23
123.10
120.52
115.31
113.86
113.83
112.11
107.42
107.38
39.93
39.52 DMSO-d6
f1 (ppm)

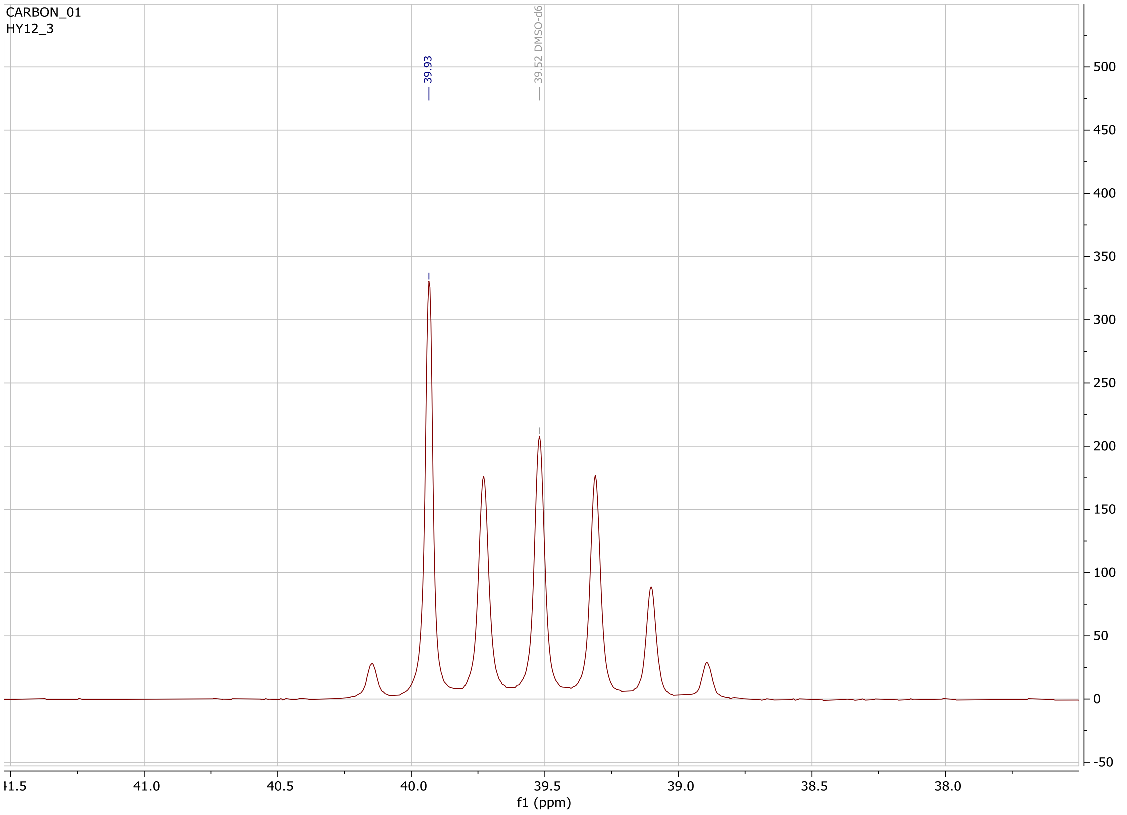
CARBON_01
HY12_3
39.93
39.52 DMSO-d6
f1 (ppm)

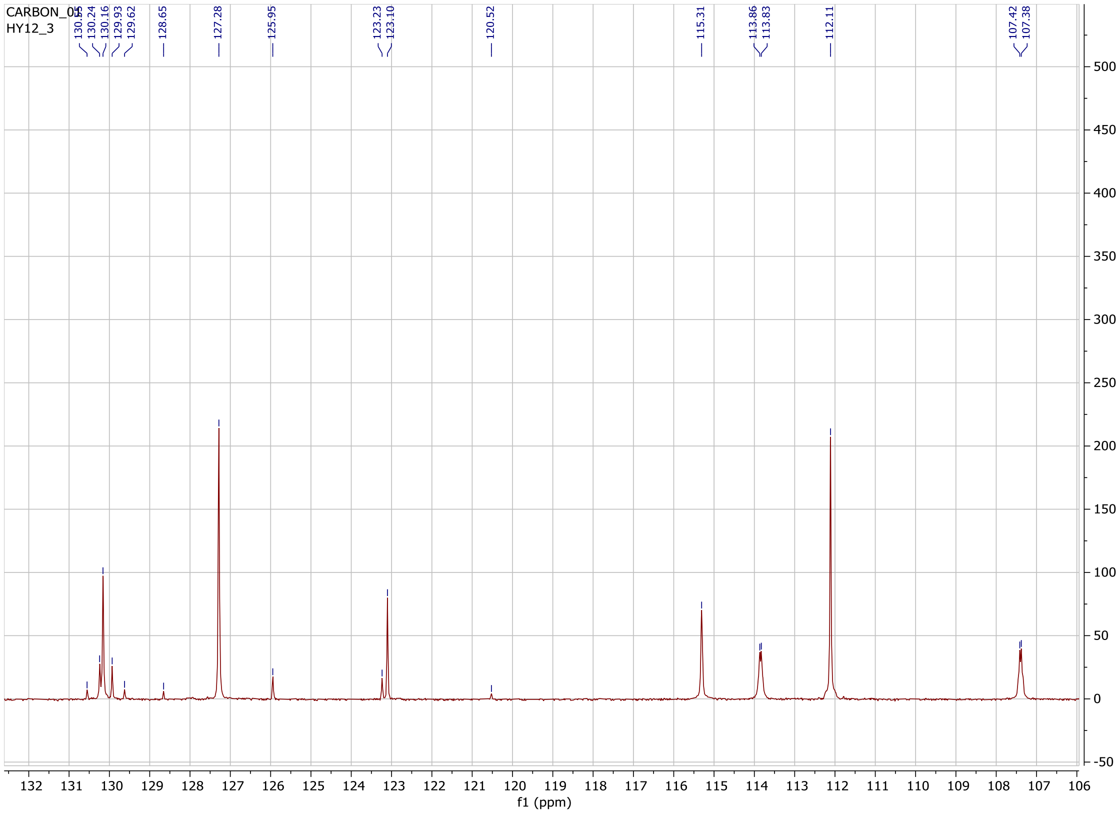

Figure S19. ^{13}C NMR of N,N-dimethyl-4-((2-(3-(trifluoromethyl)phenyl)hydrazineylidene)-methyl)aniline (**5**).

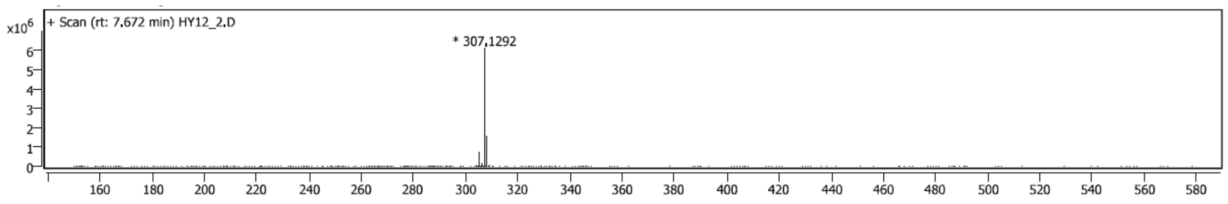

Figure S20. HRMS of N,N-dimethyl-4-((2-(3-(trifluoromethyl)phenyl)hydrazineylidene)-methyl)aniline (**5**).

***N,N*-dimethyl-4-((2-(2-(trifluoromethyl)phenyl)hydrazineylidene)methyl)aniline (6)**

^{1}H NMR (399.822 MHz, DMSO) δ (ppm) = 9.33 (s, 1H), 8.24 (s, 1H), 7.74 – 7.72 (d, 1H), 7.51 – 7.48 (m, 4H), 6.88 – 6.84 (t, 1H), 6.75 – 6.73 (d, 2H), 2.9 (s, 6H); ^{13}C NMR (DMSO, 100 MHz) δ (ppm) = 150.77, 142.99, 142.63, 133.47, 128.76 – 120.64 (q, J = 270 Hz), 127.47, 126.08 – 126.02 (q, J= 4 Hz), 122.93, 117.62, 114.40, 112.02, 110.98 – 110.08 (q, J= 30 Hz), 39.86; ^{19}F NMR (376 MHz, DMSO) δ (ppm): -59.96; HRMS (EI): m/z: [M+] calcd. for $C_{16}H_{16}F_3$: 307.1226; found: 307.1292.

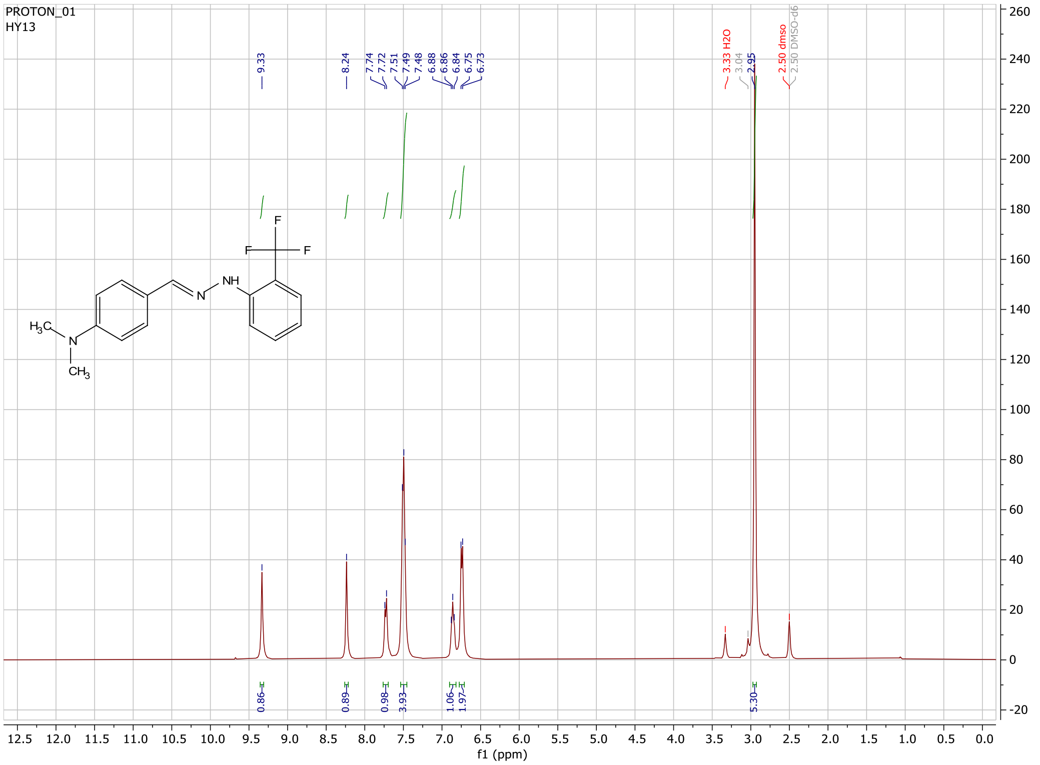

Figure S21. ^{1}H NMR of *N,N*-dimethyl-4-((2-(2-(trifluoromethyl)phenyl)hydrazineylidene) methyl)aniline (**6**).

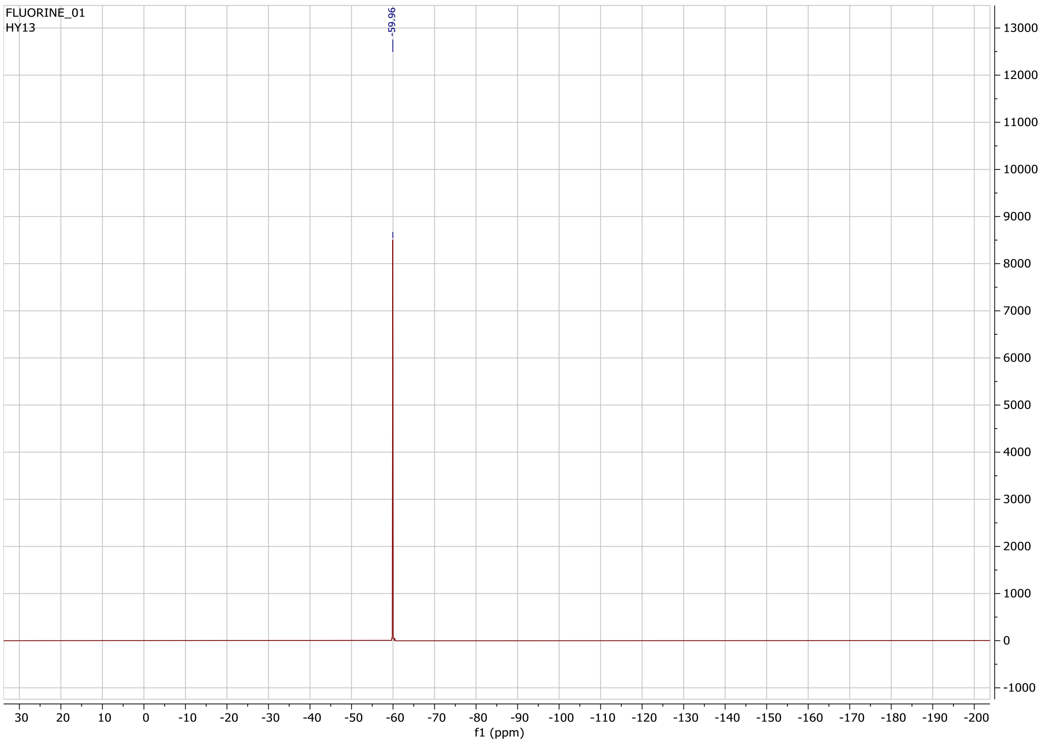

Figure S22. ^{19}F NMR of *N,N*-dimethyl-4-((2-(2-(trifluoromethyl)phenyl)hydrazineylidene) methyl)aniline (**6**).

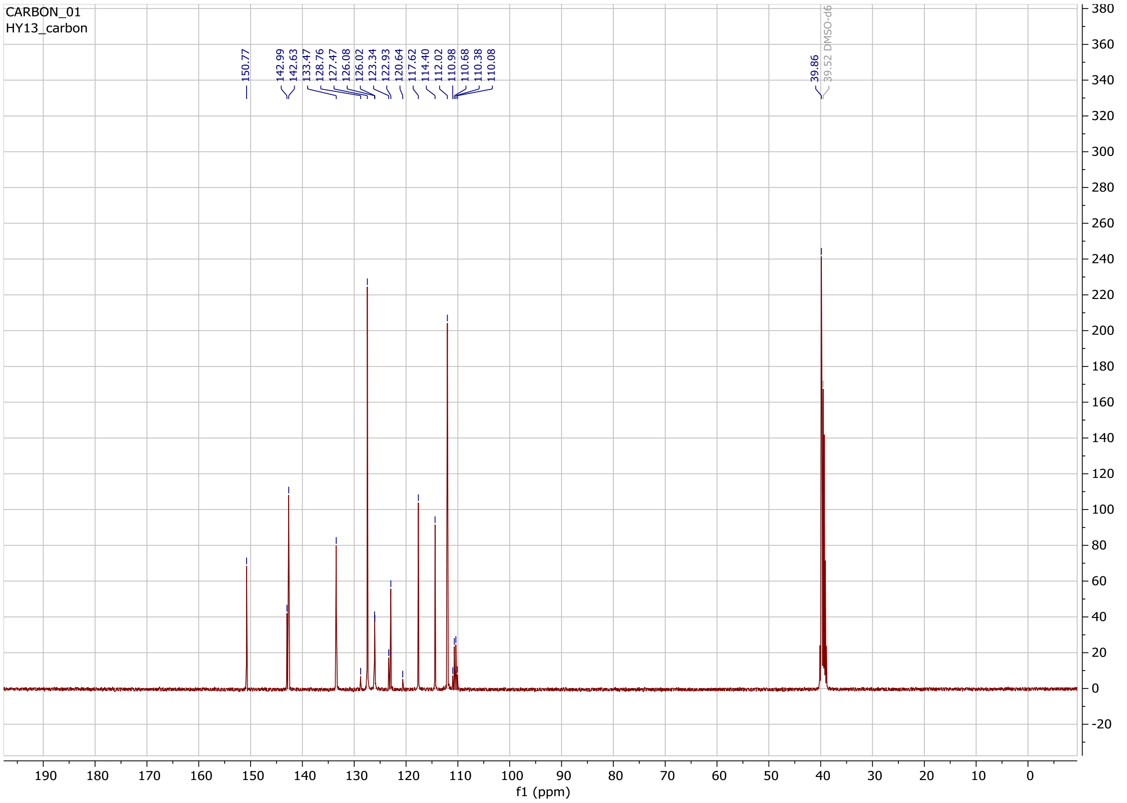

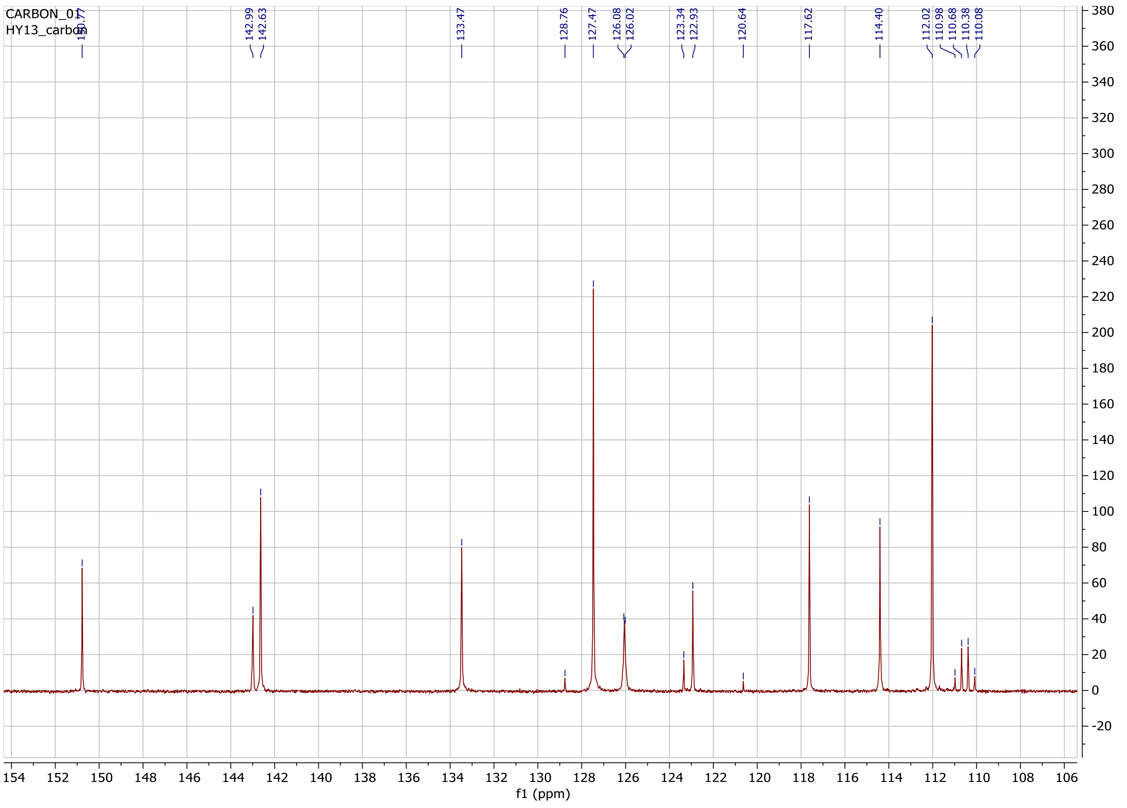

Figure S23. ^{13}C NMR of *N,N*-dimethyl-4-((2-(2-(trifluoromethyl)phenyl)hydrazineylidene) methyl)aniline (**6**).

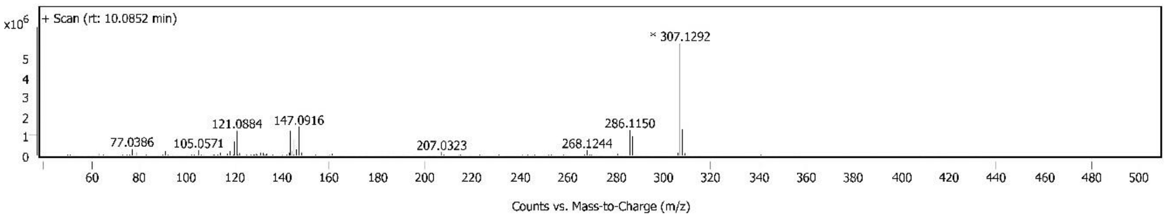

Figure S24. HRMS of *N,N*-dimethyl-4-((2-(2-(trifluoromethyl)phenyl)hydrazineylidene) methyl)aniline (**6**).

1-(4-nitrobenzylidene)-2-(3-(trifluoromethyl)phenyl)hydrazine (7)

^{1}H NMR (399.822 MHz, DMSO) δ (ppm) = 11.11 (s, 1H), 8.23-8.21 (d, 2H), 7.98 (s, 1H), 7.91-7.89 (d, 2H), 7.49 – 7.45 (t, 1H), 7.38 – 7.36 (m, 2H), 7.13-.11 (d, 1H); ^{13}C NMR (DMSO, 100 MHz) δ (ppm) = 146.43, 145.11, 141.89, 135.57, 130.25, 130.54 – 129.60 (q, J = 32 Hz), 128.29-120.16 (q, J = 271 Hz), 126.41, 123.94, 116.15, 115.74 – 115.70 (q, J = 4 Hz), 108.30-108.26 (q, J = 4 Hz); ^{19}F NMR (376 MHz, DMSO) δ (ppm): -61.50; HRMS (EI): m/z: [M+] calcd. for $C_{14}H_{10}F_3N_3O_2$: 309.0725; found: 309.0774.

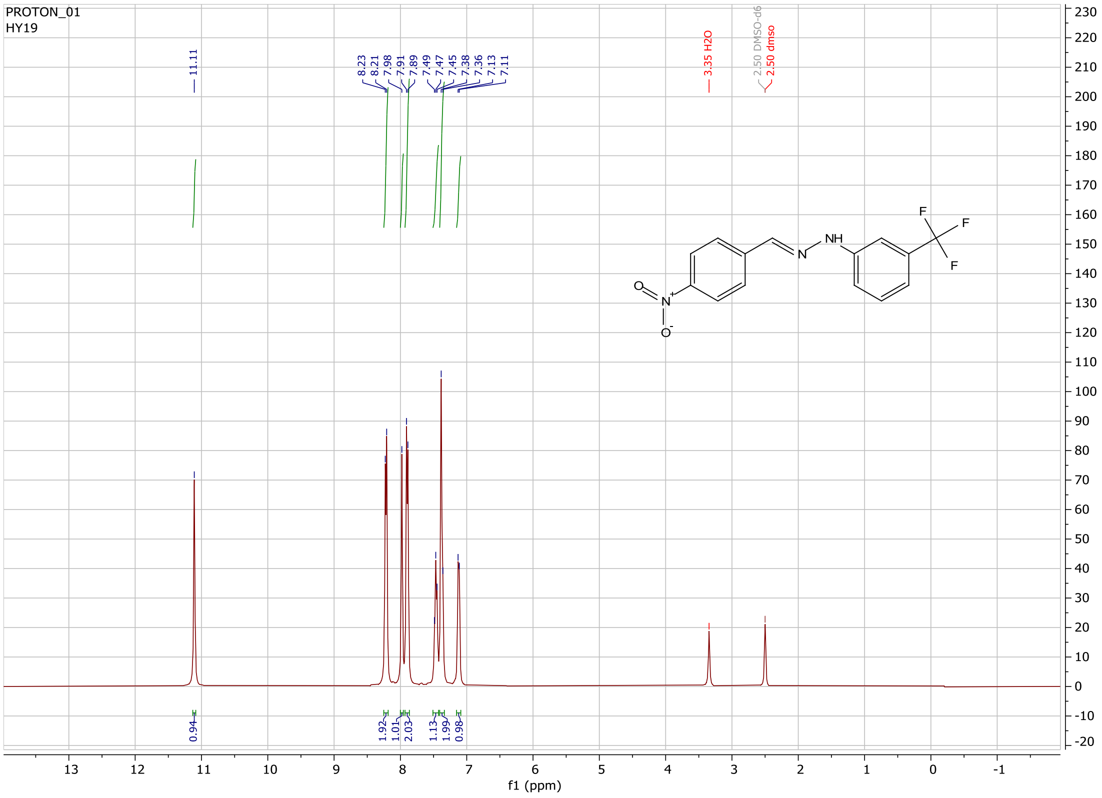

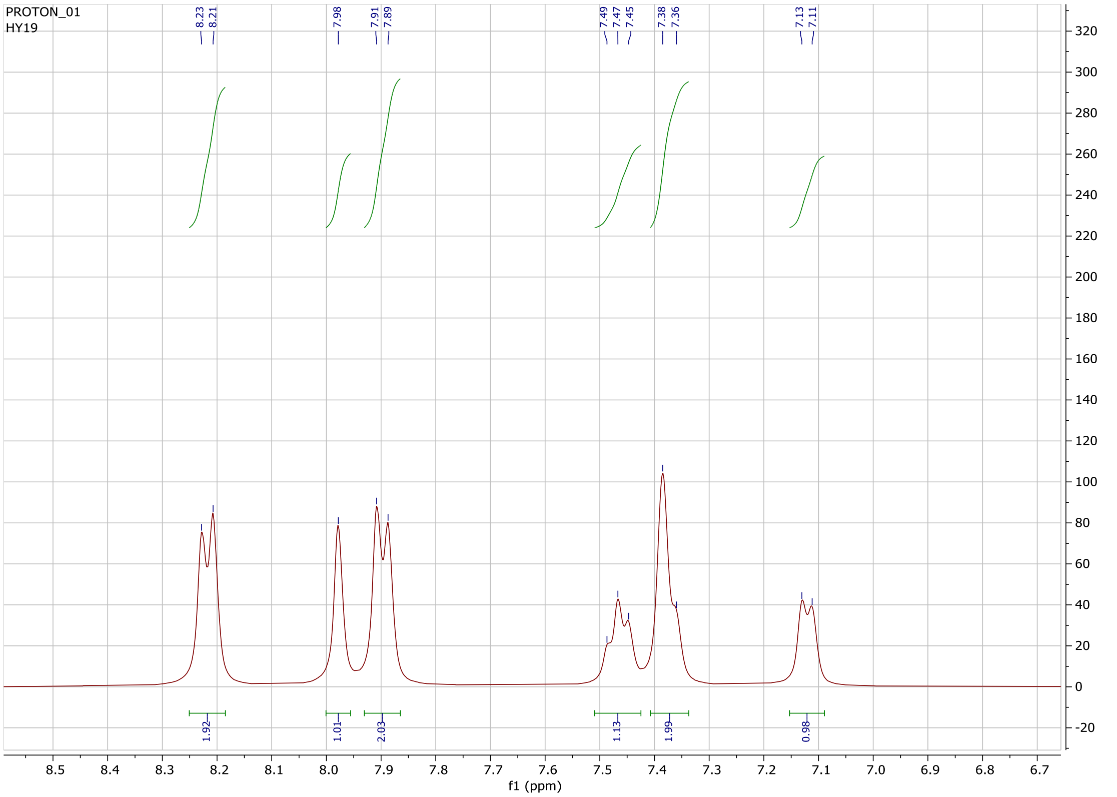

Figure S25. ^{1}H NMR of 1-(4-nitrobenzylidene)-2-(3-(trifluoromethyl)phenyl)hydrazine (**7**).

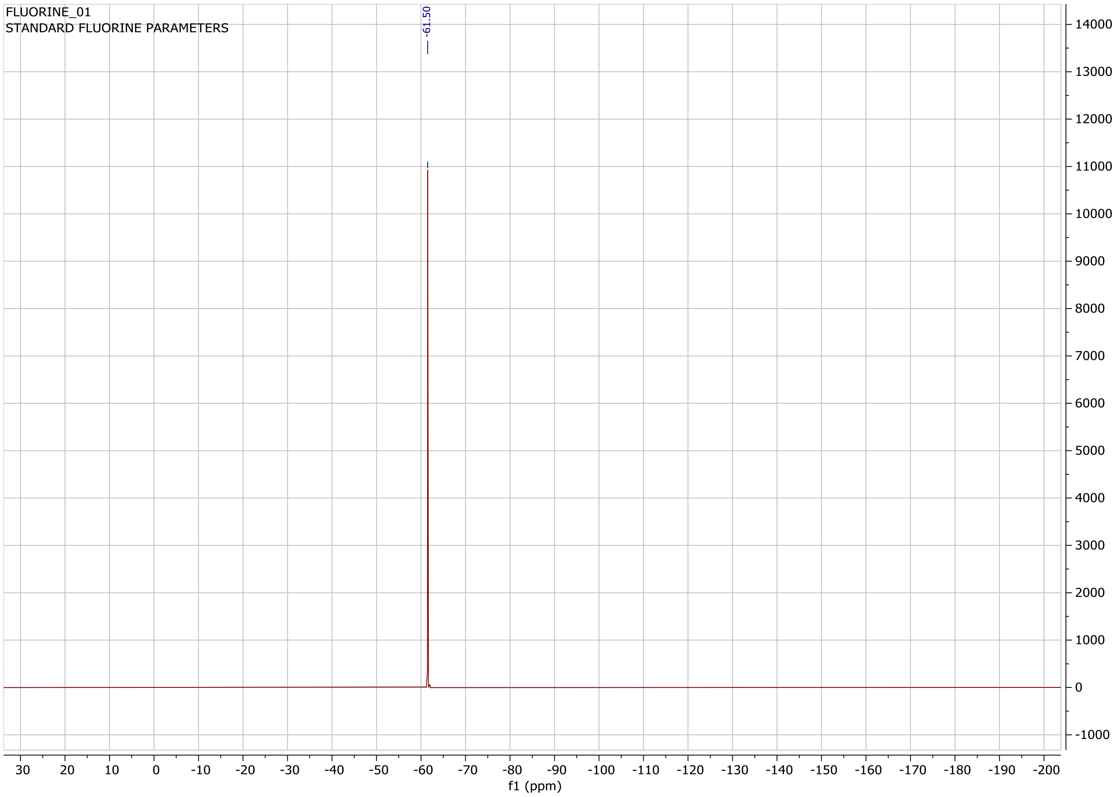

Figure S26. ^{19}F NMR of 1-(4-nitrobenzylidene)-2-(3-(trifluoromethyl)phenyl)hydrazine (**7**).

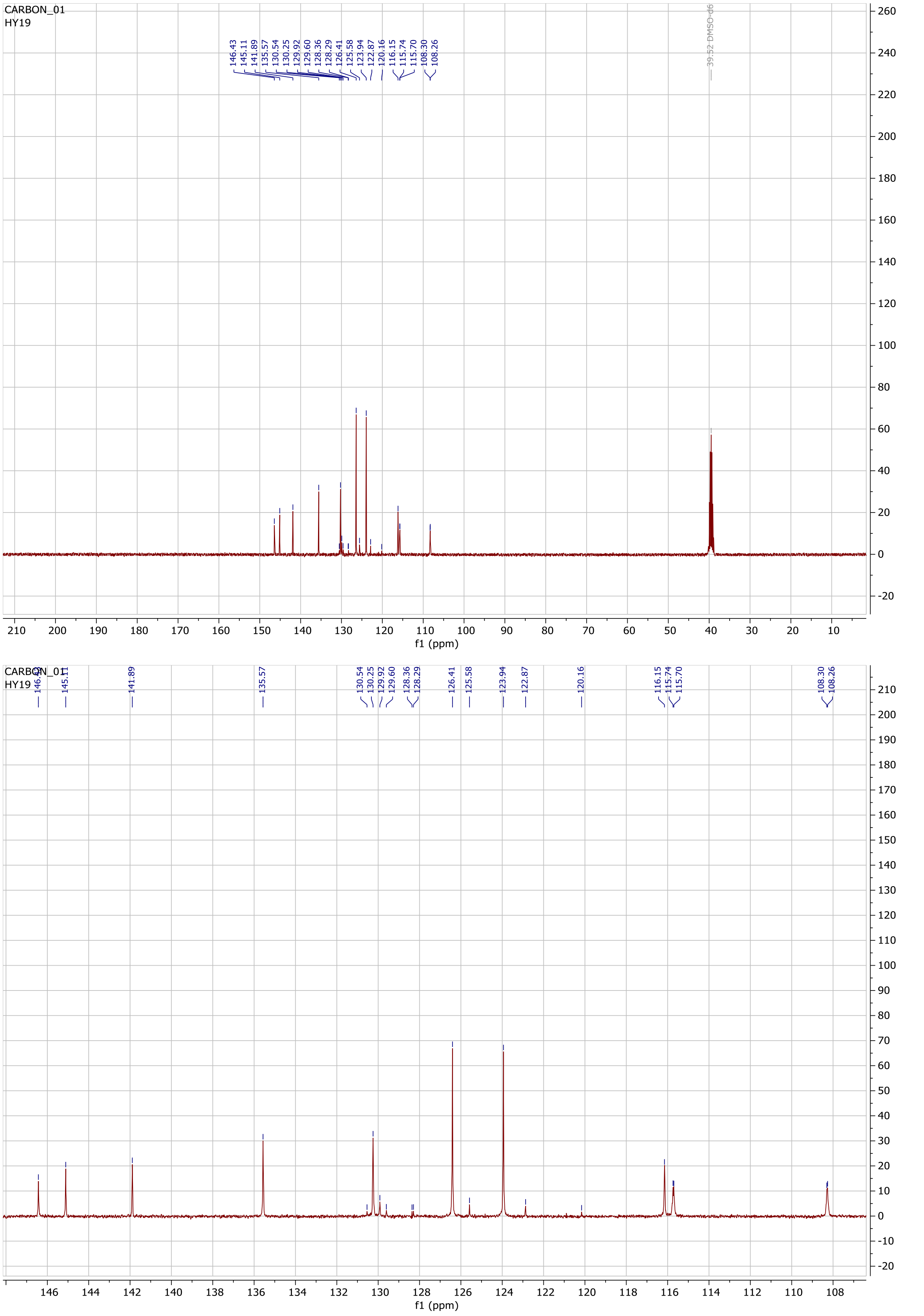

Figure S27. ^{13}C NMR of 1-(4-nitrobenzylidene)-2-(3-(trifluoromethyl)phenyl)hydrazine (**7**).

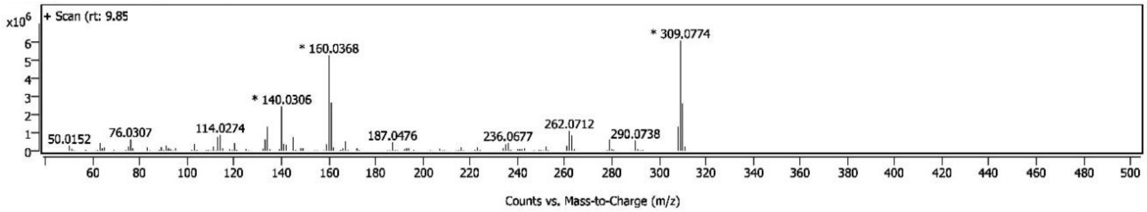

Figure S28. HRMS of 1-(4-nitrobenzylidene)-2-(3-(trifluoromethyl)phenyl)hydrazine (**7**).

<u>Mix of *E* and *Z* isomers</u> of 1-(2,4-dinitrophenyl)-2-(phenyl(4-(trifluoromethyl)phenyl) methylene)hydrazine (8)

<u>^{1}H NMR</u> (399.822 MHz, DMSO) δ (ppm) = 11.11 (s, 1H) (other isomer 10.93 (s, 1H)), 8.81 (s, 1H), 8.48 – 8.44 (s, 1H), 8.28 – 8.23 (t, 1H), 8.10 – 8.08 (d, 1H), 7.84 (s, 2H), 7.77 – 7.73 (t, 2H), 7.72 (s, 1H), 7.64 (d, 1H), 7.5 (m, 2H) ; <u>^{13}C NMR</u> (DMSO, 100 MHz) δ (ppm) = 153.52 (153.36), 144.49 (144.39), 140.56, 138.45 (138.25), 136.29 (136.12), 131.25, 131.10 (130.96), 130.58, 130.07, 129.27, 128.74 (128.57), 127.92, 127.29, 126.08, 123.20, 117.11 (117.01); <u>^{19}F NMR</u> (376 MHz, DMSO) δ (ppm): -61.25, -61.41; <u>HRMS</u> (EI): m/z: [M+] calcd. for $C_{20}H_{13}F_3N_4O_4$: 430.0889; found: 430.0297.

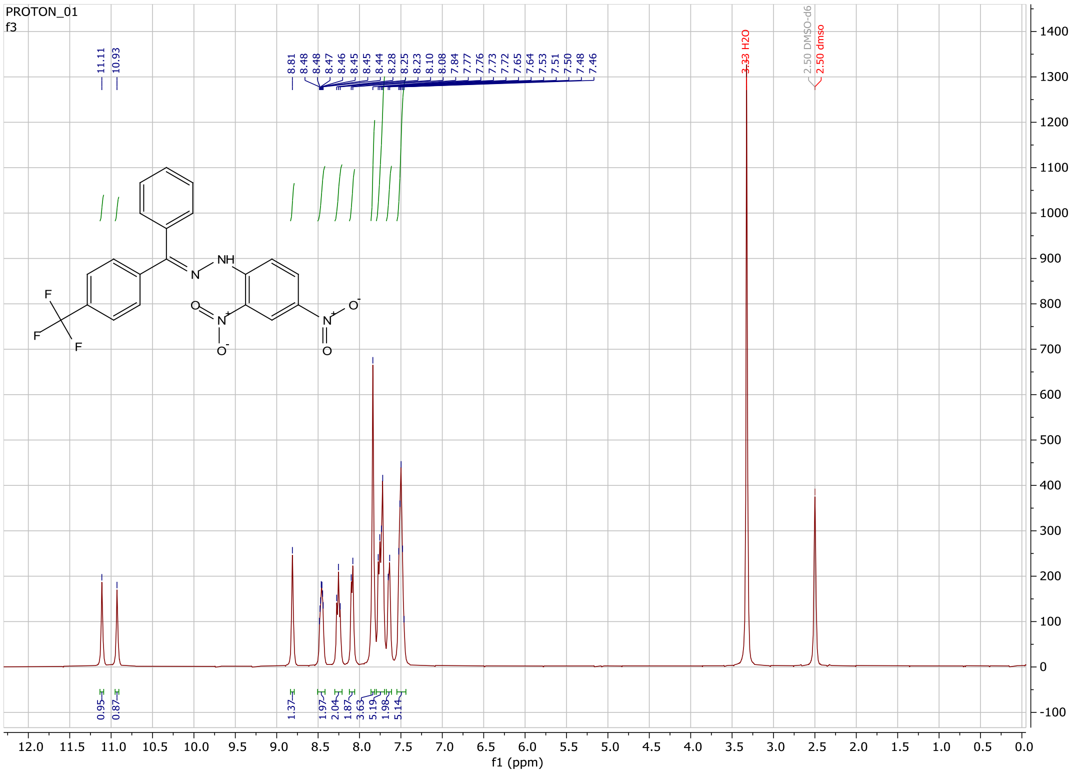

Figure S29. ^{1}H NMR of 1-(2,4-dinitrophenyl)-2-(phenyl(4-(trifluoromethyl)phenyl)methylene) hydrazine (**8**).

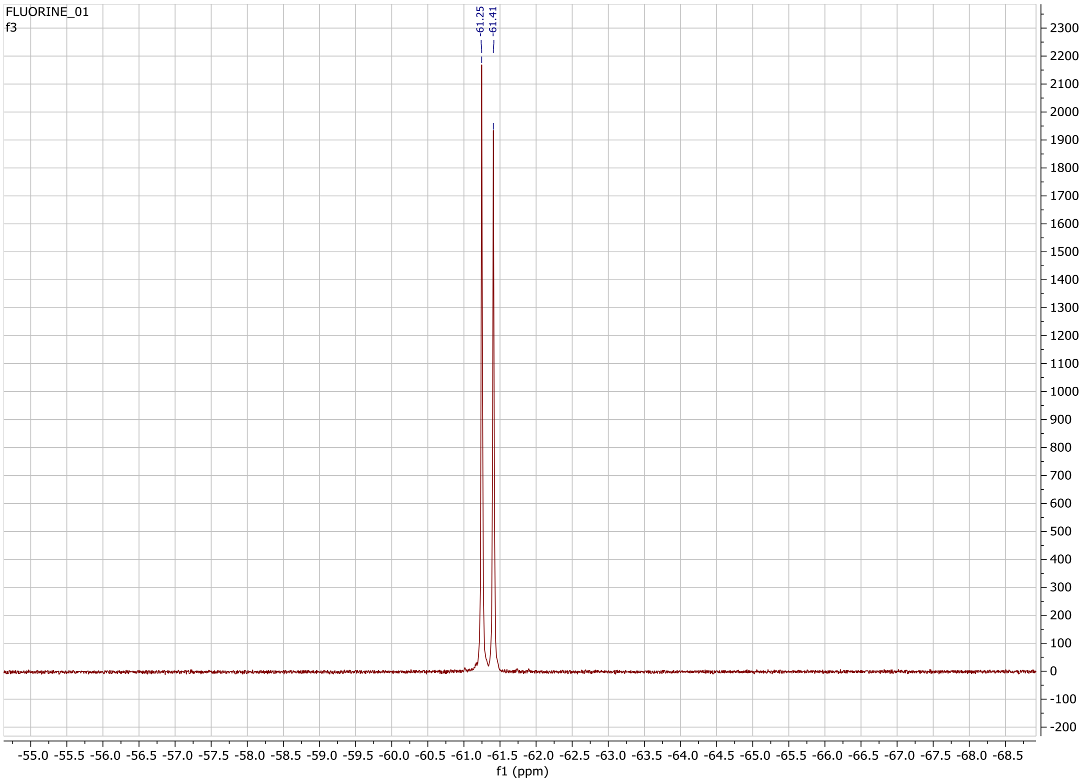

Figure S30. ^{19}F NMR of 1-(2,4-dinitrophenyl)-2-(phenyl(4-(trifluoromethyl)phenyl)methylene) hydrazine (**8**).

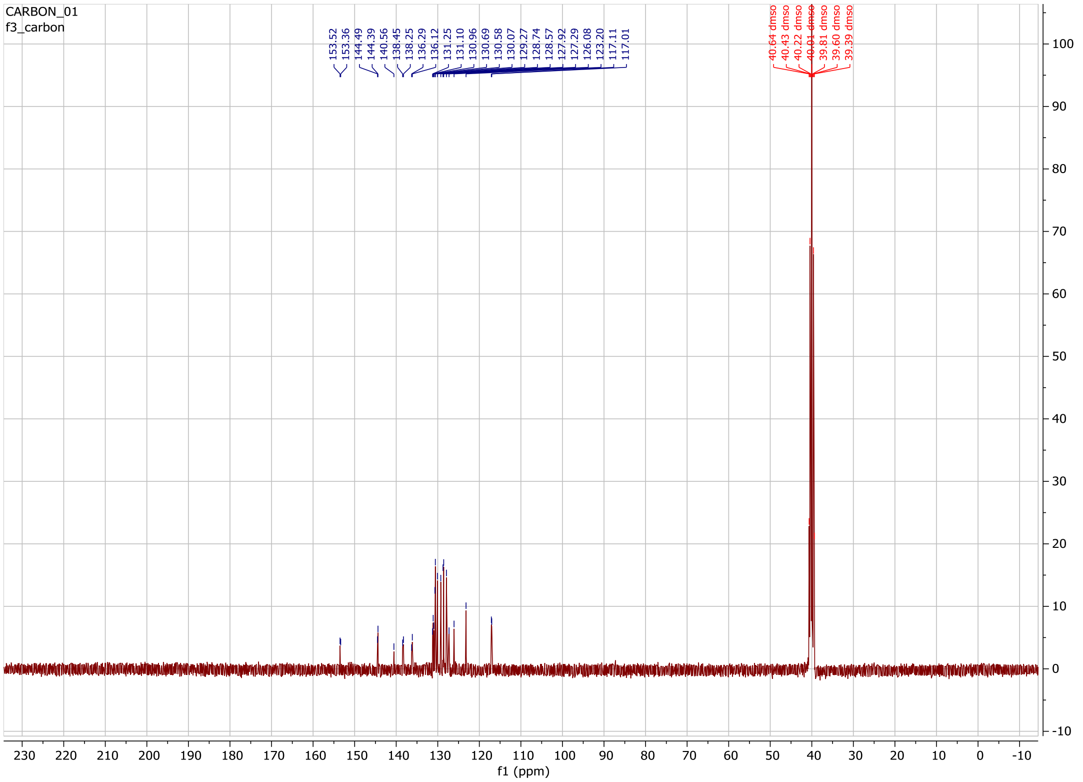

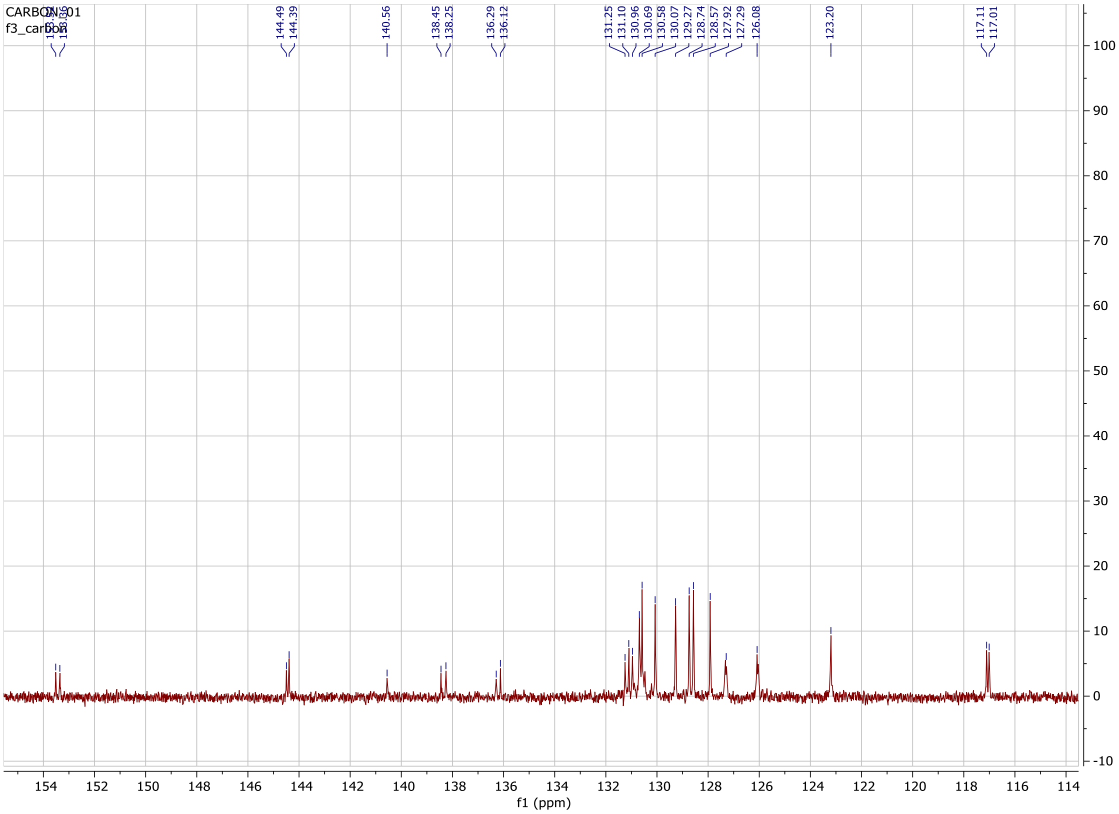

Figure S31. ^{13}C NMR of 1-(2,4-dinitrophenyl)-2-(phenyl(4-(trifluoromethyl)phenyl)methylene) hydrazine (**8**).

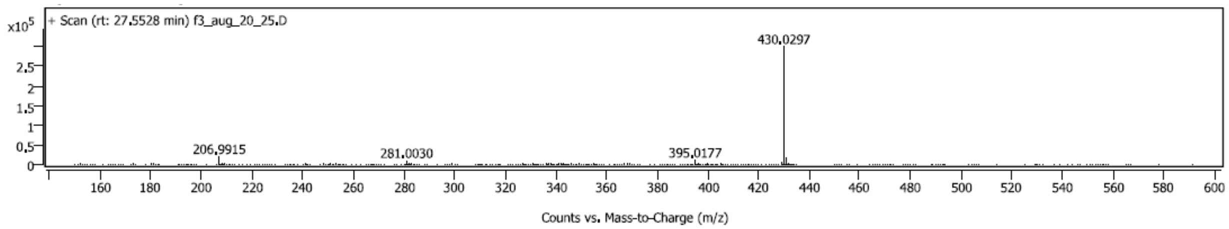

Figure S32. HRMS of 1-(2,4-dinitrophenyl)-2-(phenyl(4-(trifluoromethyl)phenyl)methylene) hydrazine (**8**).

1,4-bis-(2-(3-(trifluoromethyl)phenyl)hydrazinylidene)methyl)benzene (9)

^{1}H NMR (399.822 MHz, DMSO) δ (ppm) = 10.73 (s, 2NH), 7.93 (s, 2H), 7.71 (s, 4H), 7.46 – 7.42 (t, 2H), 7.36 (s, 2H), 7.34 – 7.32 (d, 2H), 7.07- 7.05 (d, 2H); ^{13}C NMR (DMSO, 100 MHz) δ (ppm) = 145.75, 137.94, 135.25, 130.14, 130.49-129.56 (q, J = 32 Hz), 126.20, 128.40 - 120.27 (q, J = 271 Hz), 115.66, 114.74-114.70 (q, J = 4 Hz), 107.81 – 107.77 (q, J = 4 Hz); ^{19}F NMR (376 MHz, DMSO) δ (ppm): -61.26; HRMS (EI): m/z: [M+] calcd. for $C_{22}H_{16}F_{6}N_{4}$: 450.1279; found: 450.1263.

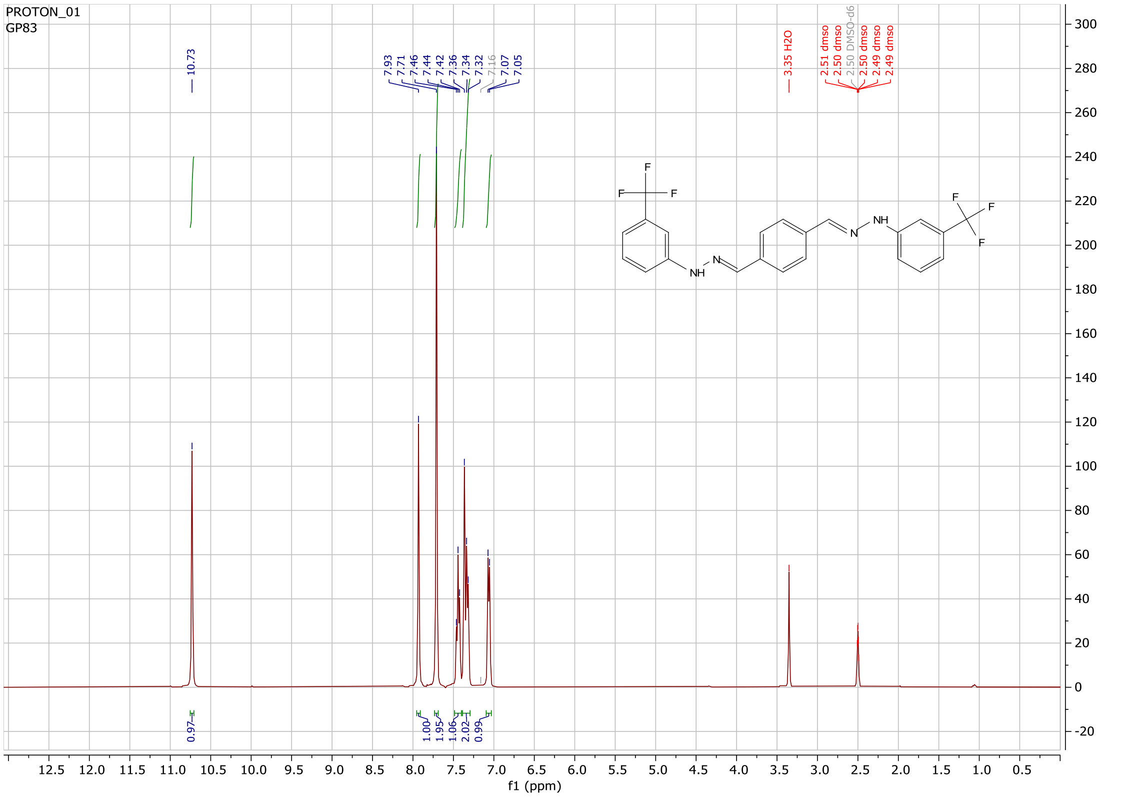

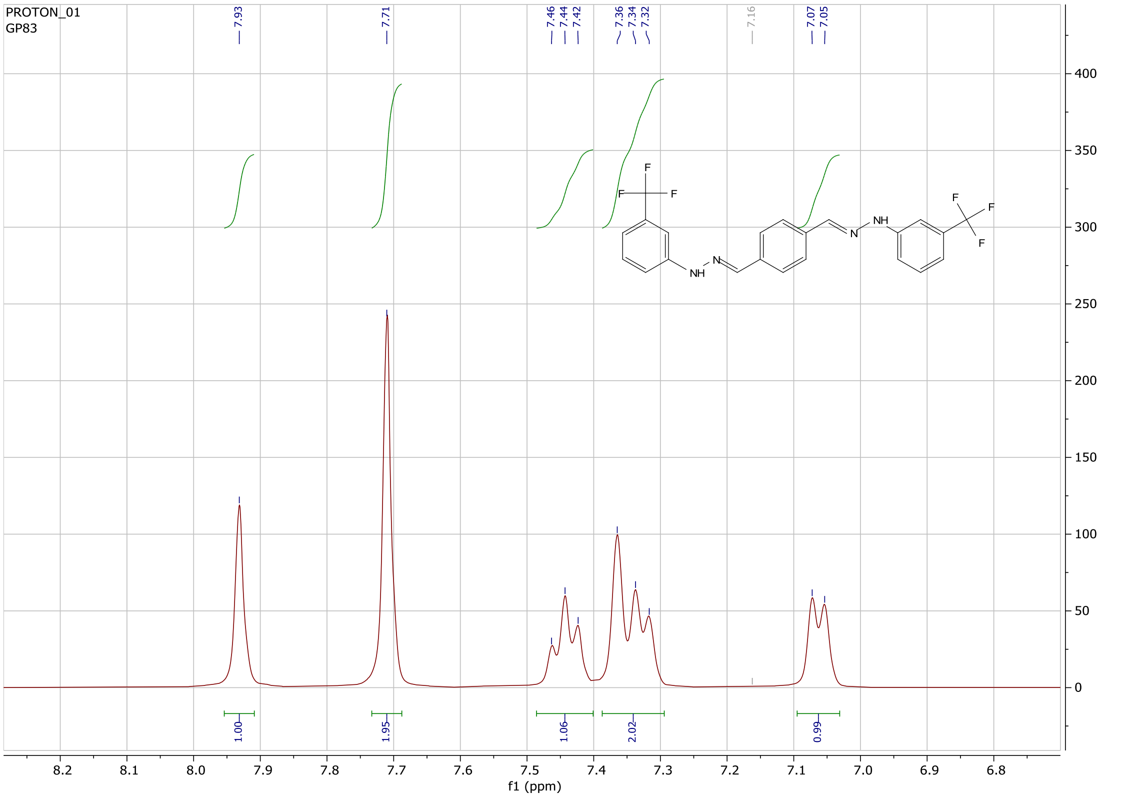

Figure S33. ^{1}H NMR of 1,4-bis(2-(3-(trifluoromethyl)phenyl)hydrazineylidene)methyl)benzene (**9**).

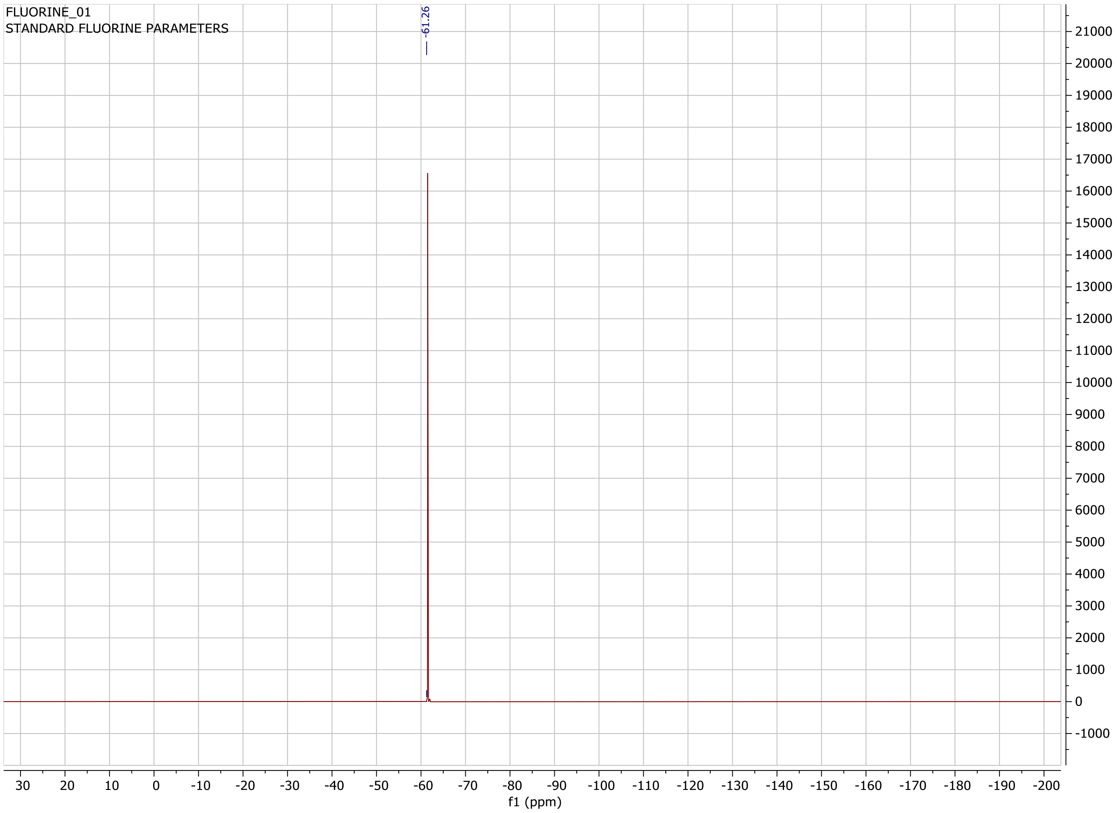

Figure S34. ^{19}F NMR of 1,4-bis(2-(3-(trifluoromethyl)phenyl)hydrazineylidene)methyl)benzene (**9**).

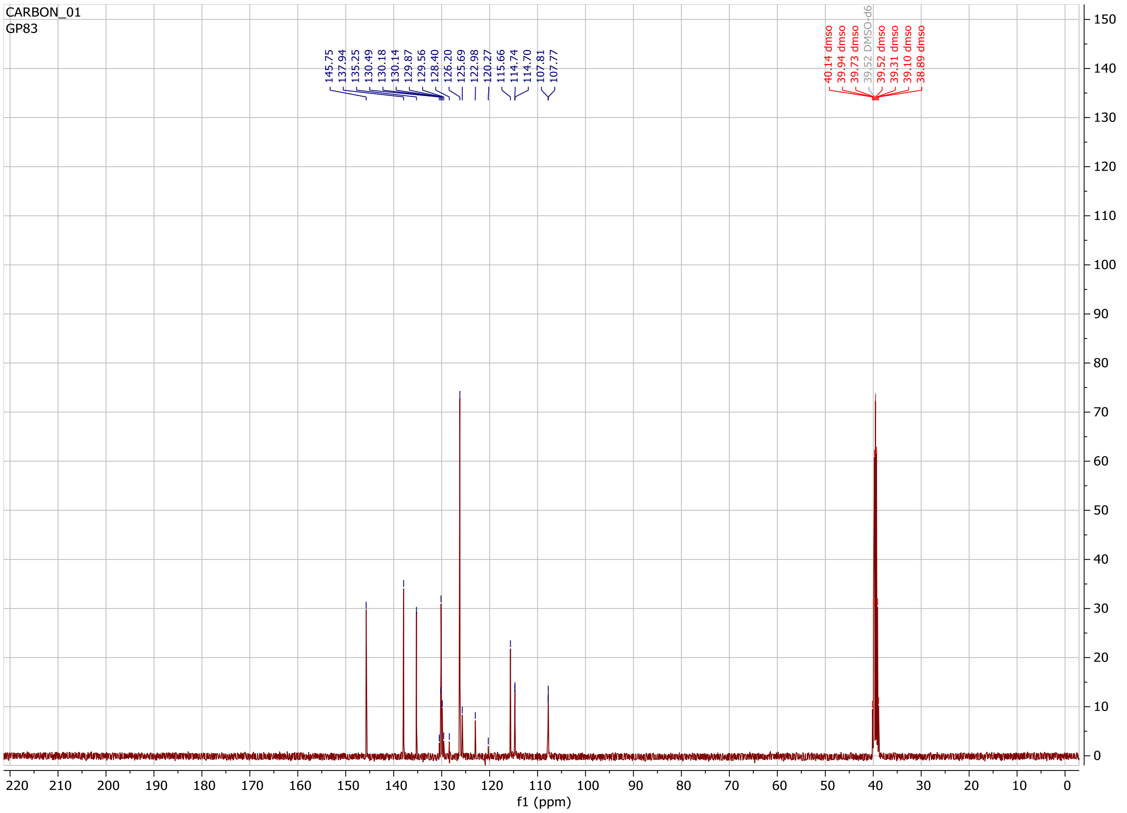

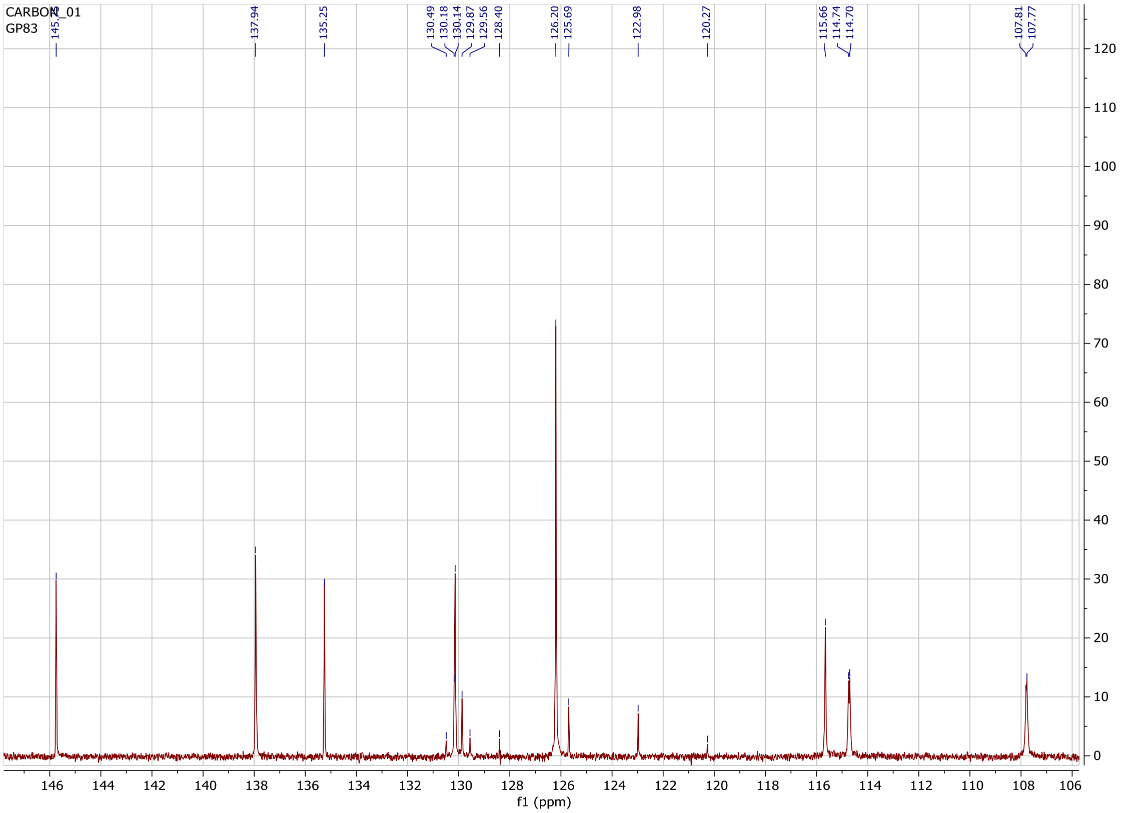

Figure S35. ^{13}C NMR of 1,4-bis(2-(3-(trifluoromethyl)phenyl)hydrazineylidene)methyl)benzene (**9**).

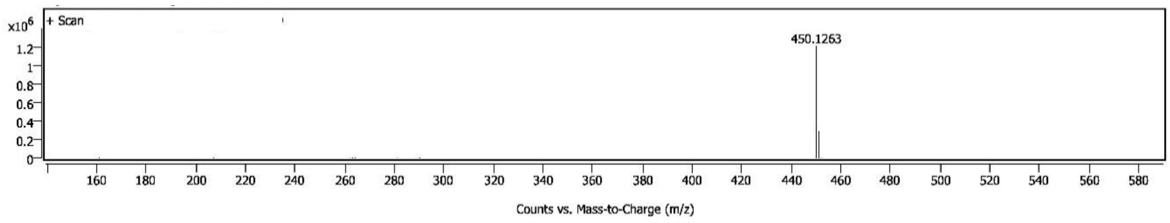

Figure S36. HRMS of 1,4-bis(2-(3-(trifluoromethyl)phenyl)hydrazineylidene)methyl)benzene (**9**).

1-(anthracen-9-ylmethylene)-2-(3-(trifluoromethyl)phenyl)hydrazine (10)

^{1}H NMR (399.822 MHz, DMSO) δ (ppm) = 11.04 (s, 1H), 9.11 (s, 1H), 8.70 – 8.67 (d, 2H), 8.64 (s, 1H), 8.15 – 8.13 (d, 2H), 7.64 – 7.55 (m, 4H), 7.52 – 7.48 (t, 1H), 7.37 (s, 1H), 7.35 (s, 1H), 7.12 – 7.10 (d, 1H); ^{13}C NMR (DMSO, 100 MHz) δ (ppm) = 145.81, 138.43, 133.07 133.04, 132.98, 130.18, 130.47 – 129.54 (q, J = 32 Hz), 128.41 – 120.28 (q, J = 271 Hz), 128.25, 127.89, 127.65, 126.50, 126.45, 126.25, 122.43, 115.68, 114.73 – 114.65 (q, J = 4 Hz), 107.80 – 107.76 (q, J = 4 Hz); ^{19}F NMR (376 MHz, DMSO) δ (ppm): -61.42; HRMS (EI): m/z: [M+] calcd. for $C_{22}H_{15}F_3N_2$: 364.1187; found: 364.1190.

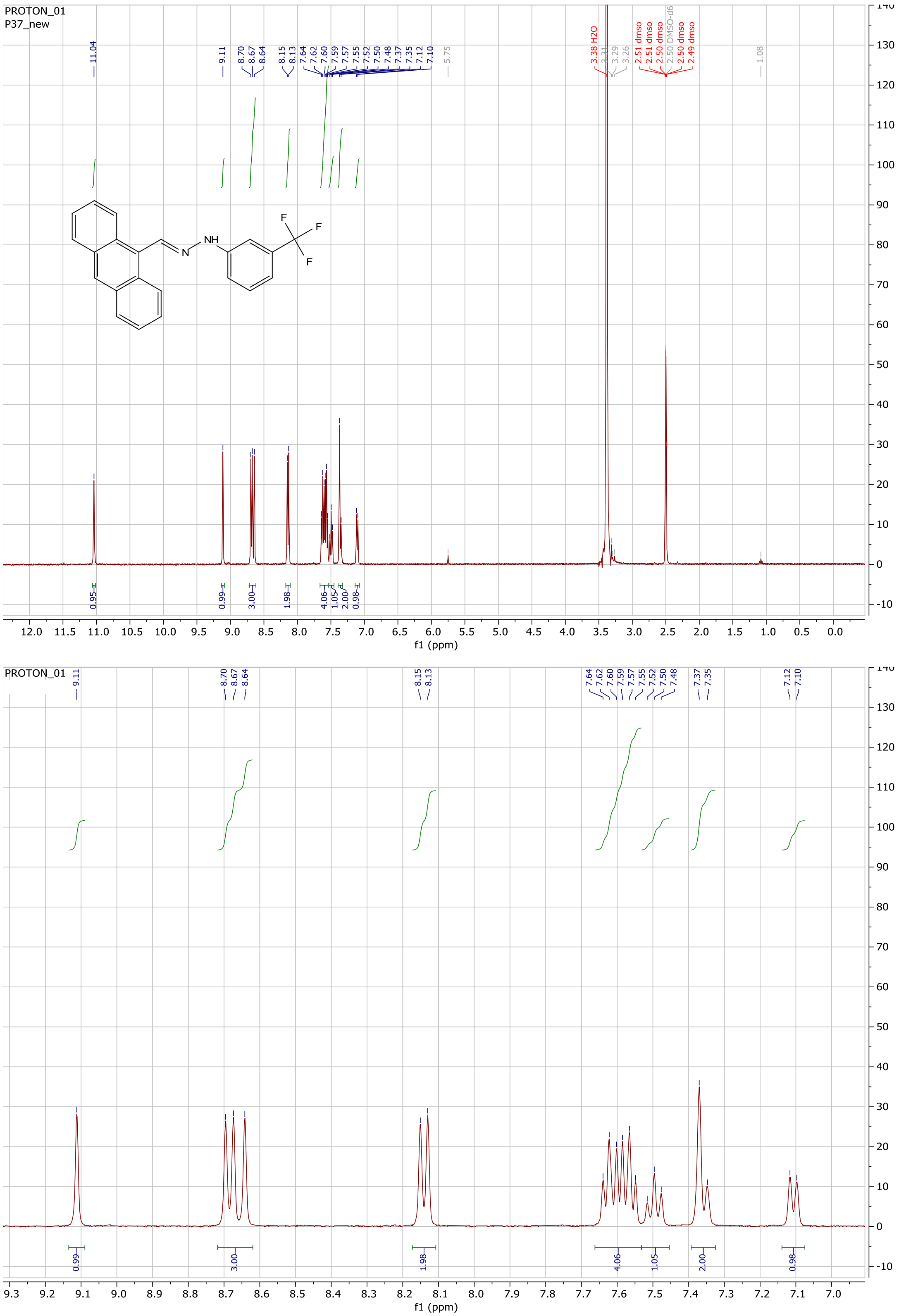

Figure S37. ^{1}H NMR of 1-(anthracen-9-ylmethylene)-2-(3-(trifluoromethyl)phenyl)hydrazine (**10**).

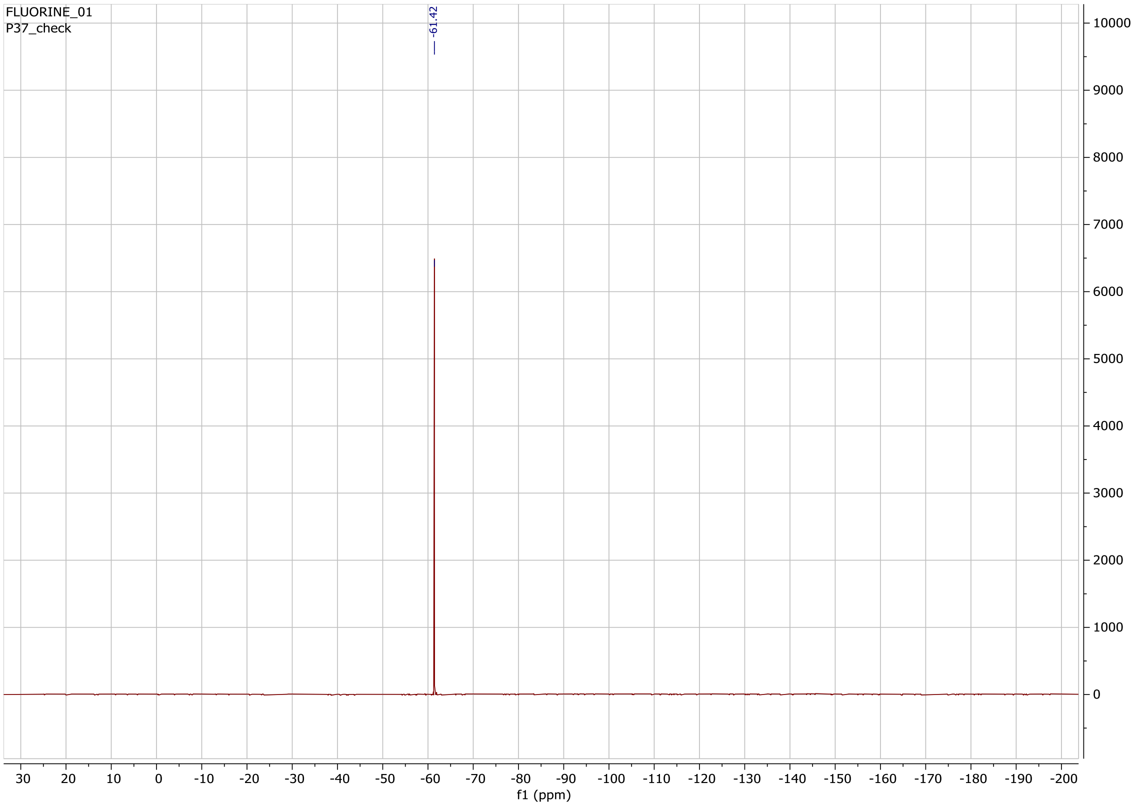

Figure S38. ^{19}F NMR of 1-(anthracen-9-ylmethylene)-2-(3-(trifluoromethyl)phenyl)hydrazine (**10**).

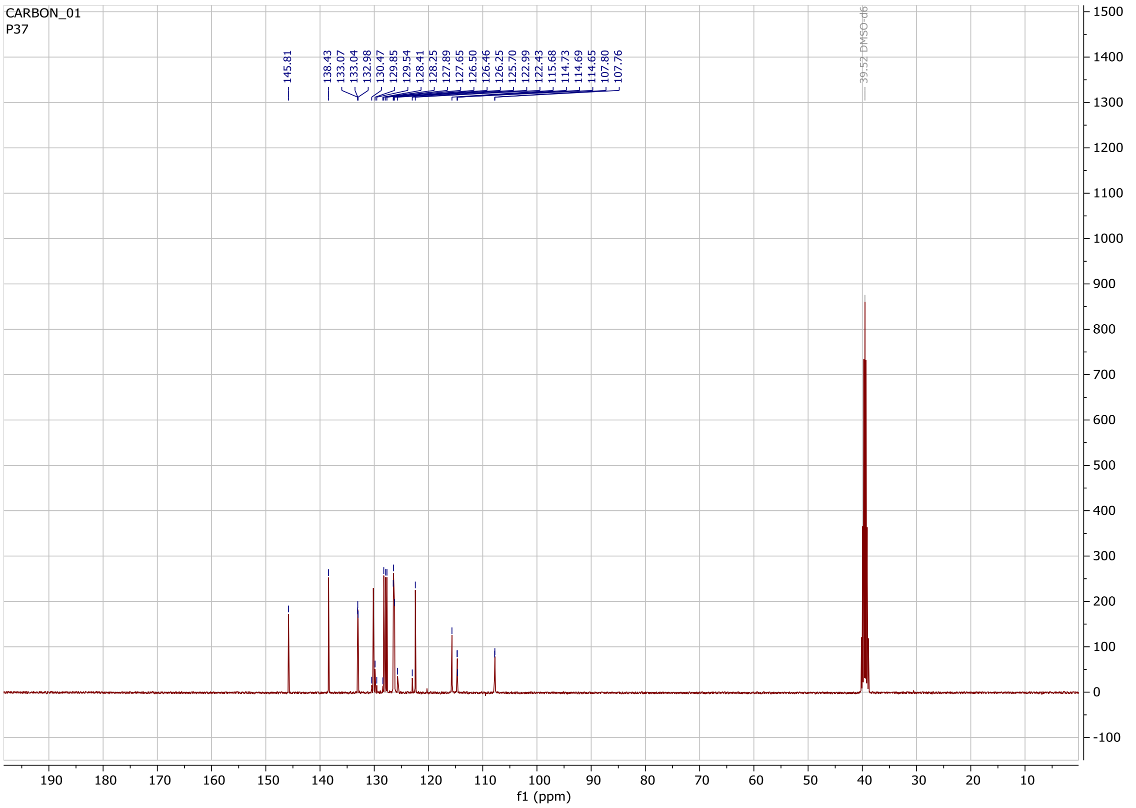

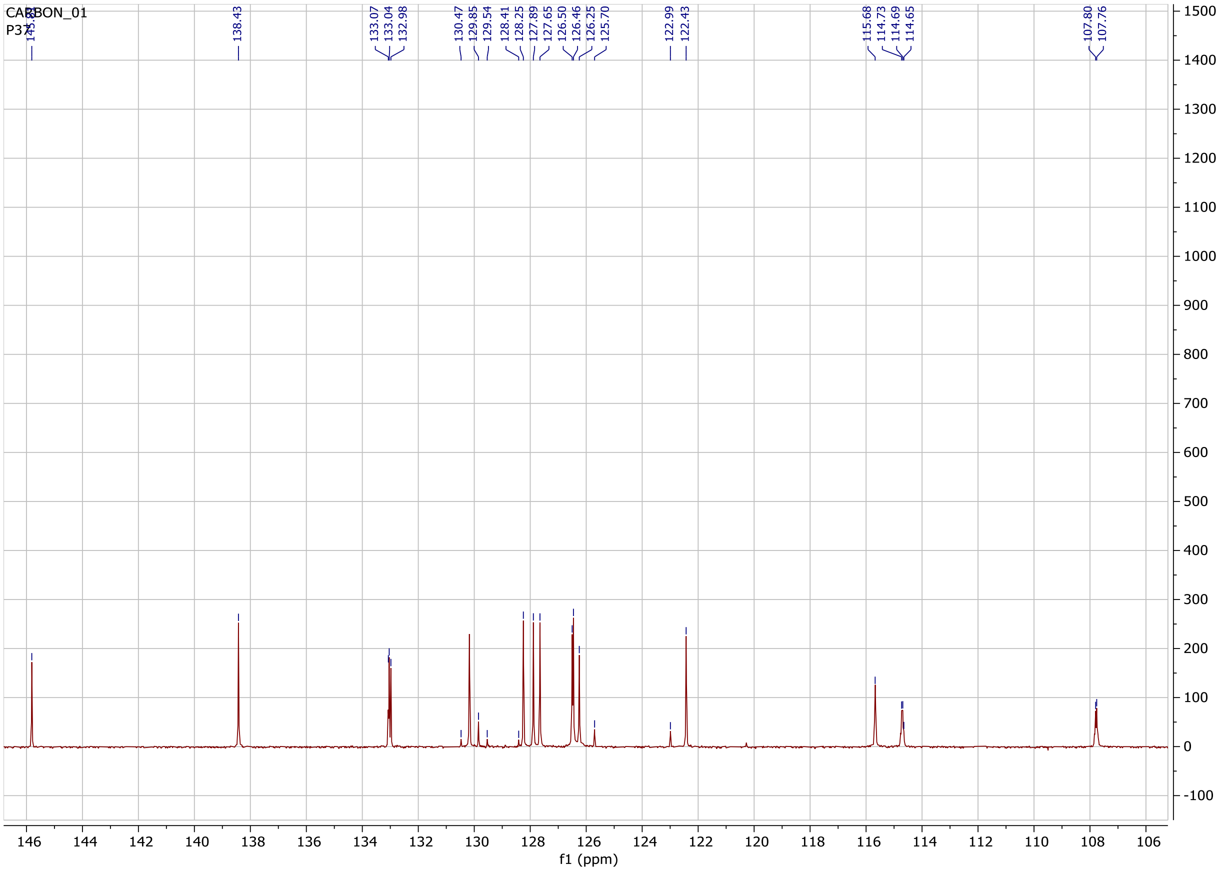

Figure S39. ^{13}C NMR of 1-(anthracen-9-ylmethylene)-2-(3-(trifluoromethyl)phenyl)hydrazine (**10**).

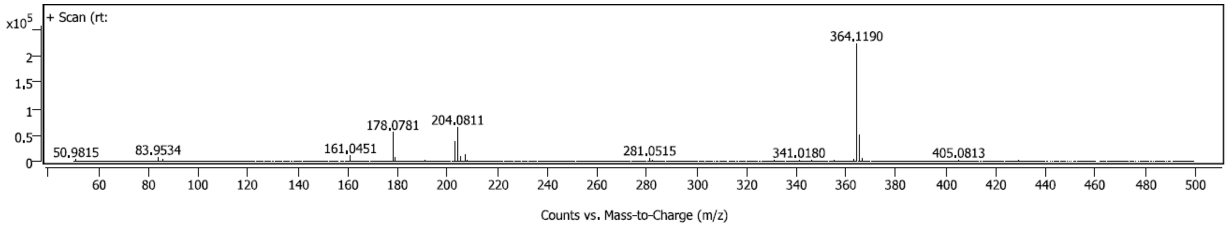

Figure S40. HRMS of 1-(anthracen-9-ylmethylene)-2-(3-(trifluoromethyl)phenyl)hydrazine (**10**).

2. Additional assay/analysis data

2.1. Numerical data used for plotting the dose–response curves

DPPH

Plate [ ] (μM)	Ascorbic Acid	Trolox	1	2	3	4	5	6	7	8	9	10
50	84.177	91.720	86.785	84.194	89.736	48.818	90.487	86.107	60.863	0.000	75.720	30.047
25	41.121	48.333	63.874	73.633	67.234	23.277	67.188	58.597	36.788	0.000	34.979	22.968
12.5	18.639	12.534	34.462	38.333	37.816	12.483	34.118	25.094	37.692	0.000	21.556	12.458
6.25	5.143	5.218	11.935	14.053	12.439	5.460	29.924	9.278	4.922	0.000	8.549	3.950
3.13	0.000	3.491	3.751	0.000	9.640	2.244	1.662	0.042	0.000	0.000	1.690	0.504

ABTS

Plate [ ] (μM)	Ascorbic Acid	Trolox	1	2	3	4	5	6	7	8	9	10
20	27.810	35.717	44.466	46.503	64.340	56.740	73.545	57.982	59.697	21.535	37.207	54.996
10	13.034	17.503	28.533	30.819	39.635	24.277	47.703	38.175	41.565	4.715	24.287	33.805
5	5.543	8.302	19.869	20.159	31.129	11.607	28.437	26.589	25.900	3.797	19.227	14.859
2.5	2.053	3.332	10.400	9.850	19.828	5.854	13.750	9.172	14.579	-1.133	13.129	5.362
1.25	0.484	0.089	4.403	1.736	6.139	0.962	4.280	3.273	3.071	-1.838	8.975	1.257

ORAC

Plate [ ] (μM)	Ascorbic Acid	Trolox	1	2	3	4	5	6	7	8	9	10
50	88.064	94.742	95.666	97.260	74.820	105.588	88.195	76.280	88.745	60.060	57.855	37.466
10	42.546	92.888	94.500	94.662	75.716	95.305	81.882	81.726	79.505	19.596	44.469	26.445
1.56	0.000	84.873	93.287	96.189	65.128	55.912	82.328	85.417	41.893	3.296	34.849	26.516
0.156	0.000	24.485	36.526	46.813	11.985	25.860	21.748	42.806	6.199	0.888	20.462	4.309
0.0488	0.000	9.438	9.261	25.676	0.000	10.543	1.834	12.247	0.000	0.000	3.985	0.000

2.2. Description of the dose–response curves

DPPH:

Model	DoseResp
Equation	y = A1 + (A2-A1)/(1 + 10^((LOGx0-x)*p))
Plot	**Ascorbic Acid**
A1	6.30228 ± 4.7638
A2	89.4827 ± 9.86164
LOGx0	1.42457 ± 0.05315
p	4.15481 ± 0
Reduced Chi-Sqr	61.35694
R-Square (COD)	0.97392
Adj. R-Square	0.94784

Model	DoseResp
Equation	y = A1 + (A2-A1)/(1 + 10^((LOGx0-x)*p))
Plot	**Trolox**
A1	3.62955 ± 0.4153
A2	105.8177 ± 1.95872
LOGx0	1.43435 ± 0.0074
p	3.00778 ± 0.12953
Reduced Chi-Sqr	0.23642
R-Square (COD)	0.99996
Adj. R-Square	0.99984

Model	DoseResp
Equation	y = A1 + (A2-A1)/(1 + 10^((LOGx0-x)*p))
Plot	**1**
A1	-1.01249 ± 1.85771
A2	101.53512 ± 3.98445
LOGx0	1.26016 ± 0.02048
p	1.7572 ± 0.1506
Reduced Chi-Sqr	0.92804
R-Square (COD)	0.99981
Adj. R-Square	0.99924

Model	DoseResp
Equation	y = A1 + (A2-A1)/(1 + 10^((LOGx0-x)*p))
Plot	**2**
A1	-2.05615 ± 6.08877
A2	89.41555 ± 6.89979
LOGx0	1.12485 ± 0.04621
p	2.27585 ± 0.61803
Reduced Chi-Sqr	14.9172
R-Square (COD)	0.99721
Adj. R-Square	0.98882

Model	DoseResp
Equation	y = A1 + (A2-A1)/(1 + 10^((LOGx0-x)*p))
Plot	**3**
A1	5.7443 ± 5.4009
A2	98.85328 ± 10.69349
LOGx0	1.24908 ± 0.06063
p	2.09654 ± 0.60982
Reduced Chi-Sqr	14.49333
R-Square (COD)	0.99701
Adj. R-Square	0.98803

Model	DoseResp
Equation	y = A1 + (A2-A1)/(1 + 10^((LOGx0-x)*p))
Plot	**4**
A1	5.96554 ± 2.67002
A2	52.48778 ± 5.83172
LOGx0	1.44577 ± 0.05591
p	4.15481 ± 0
Reduced Chi-Sqr	19.49916
R-Square (COD)	0.97239
Adj. R-Square	0.94478

Model	DoseResp
Equation	y = A1 + (A2-A1)/(1 + 10^((LOGx0-x)*p))
Plot	**5**
A1	17.07637 ± 10.31114
A2	88.55925 ± 15.34514
LOGx0	1.26923 ± 0.14086
p	4.15481 ± 0
Reduced Chi-Sqr	226.03468
R-Square (COD)	0.90552
Adj. R-Square	0.81104

Model	DoseResp
Equation	y = A1 + (A2-A1)/(1 + 10^((LOGx0-x)*p))
Plot	**6**
A1	-2.13402 ± 3.42986
A2	106.28472 ± 11.19786
LOGx0	1.34488 ± 0.05369
p	1.82062 ± 0.34167
Reduced Chi-Sqr	4.55186
R-Square (COD)	0.99912
Adj. R-Square	0.99646

Model	DoseResp
Equation	y = A1 + (A2-A1)/(1 + 10^((LOGx0-x)*p))
Plot	**7**
A1	-0.90661 ± 10.20207
A2	50.03591 ± 8.54584
LOGx0	0.99491 ± 0.13966
p	4.15481 ± 0
Reduced Chi-Sqr	135.29207
R-Square (COD)	0.89462
Adj. R-Square	0.78924

Model	DoseResp
Equation	y = A1 + (A2-A1)/(1 + 10^((LOGx0-x)*p))
Plot	**9**
A1	9.38578 ± 5.38205
A2	81.75202 ± 11.9863
LOGx0	1.45238 ± 0.07391
p	4.15481 ± 0
Reduced Chi-Sqr	79.47586
R-Square (COD)	0.95375
Adj. R-Square	0.9075

Model	DoseResp
Equation	y = A1 + (A2-A1)/(1 + 10^((LOGx0-x)*p))
Plot	**10**
A1	-1.25738 ± 0.38335
A2	34.03356 ± 0.64208
LOGx0	1.20835 ± 0.00976
p	1.81737 ± 0.08439
Reduced Chi-Sqr	0.03799
R-Square (COD)	0.99994
Adj. R-Square	0.99976

ABTS:

Model	DoseResp
Equation	y = A1 + (A2-A1)/(1 + 10^((LOGx0-x)*p))
Plot	**Ascorbic Acid**
A1	2.23925 ± 1.2511
A2	29.86784 ± 2.68242
LOGx0	1.04091 ± 0.04334
p	4.15241 ± 0
Reduced Chi-Sqr	4.26555
R-Square (COD)	0.98293
Adj. R-Square	0.96586

Model	DoseResp
Equation	y = A1 + (A2-A1)/(1 + 10^((LOGx0-x)*p))
Plot	**Trolox**
A1	3.25029 ± 2.09268
A2	38.03464 ± 4.37408
LOGx0	1.03067 ± 0.0563
p	4.15241 ± 0
Reduced Chi-Sqr	11.86674
R-Square (COD)	0.97101
Adj. R-Square	0.94201

Model	DoseResp
Equation	y = A1 + (A2-A1)/(1 + 10^((LOGx0-x)*p))
Plot	**1**
A1	10.18605 ± 4.1737
A2	45.04817 ± 7.67935
LOGx0	0.96326 ± 0.10884
p	4.15241 ± 0
Reduced Chi-Sqr	44.51358
R-Square (COD)	0.91053
Adj. R-Square	0.82106

Model	DoseResp
Equation	y = A1 + (A2-A1)/(1 + 10^((LOGx0-x)*p))
Plot	**2**
A1	5.56193 ± 5.55077
A2	41.16831 ± 6.10285
LOGx0	0.7633 ± 0.11526
p	4.15241 ± 0
Reduced Chi-Sqr	54.82422
R-Square (COD)	0.91155
Adj. R-Square	0.82309

Model	DoseResp
Equation	y = A1 + (A2-A1)/(1 + 10^((LOGx0-x)*p))
Plot	**3**
A1	17.52244 ± 7.01941
A2	66.27888 ± 13.74361
LOGx0	0.99901 ± 0.12975
p	4.15241 ± 0
Reduced Chi-Sqr	130.59663
R-Square (COD)	0.86408
Adj. R-Square	0.72816

Model	DoseResp
Equation	y = A1 + (A2-A1)/(1 + 10^((LOGx0-x)*p))
Plot	**4**
A1	5.39631 ± 2.73503
A2	62.17938 ± 6.37533
LOGx0	1.06797 ± 0.05023
p	4.15241 ± 0
Reduced Chi-Sqr	20.63923
R-Square (COD)	0.97937
Adj. R-Square	0.95874

Model	DoseResp
Equation	y = A1 + (A2-A1)/(1 + 10^((LOGx0-x)*p))
Plot	**5**
A1	12.8477 ± 6.25643
A2	74.00507 ± 11.12971
LOGx0	0.94407 ± 0.09488
p	4.15241 ± 0
Reduced Chi-Sqr	97.37279
R-Square (COD)	0.93667
Adj. R-Square	0.87333

Model	DoseResp
Equation	y = A1 + (A2-A1)/(1 + 10^((LOGx0-x)*p))
Plot	**6**
A1	5.55786 ± 6.70208
A2	50.59156 ± 7.01565
LOGx0	0.73275 ± 0.10221
p	4.15241 ± 0
Reduced Chi-Sqr	77.6377
R-Square (COD)	0.92101
Adj. R-Square	0.84201

Model	DoseResp
Equation	y = A1 + (A2-A1)/(1 + 10^((LOGx0-x)*p))
Plot	**7**
A1	8.65568 ± 6.57013
A2	54.06141 ± 7.4195
LOGx0	0.77711 ± 0.11152
p	4.15241 ± 0
Reduced Chi-Sqr	77.84327
R-Square (COD)	0.92177
Adj. R-Square	0.84354

Model	DoseResp
Equation	y = A1 + (A2-A1)/(1 + 10^((LOGx0-x)*p))
Plot	**8**
A1	0.06534 ± 1.72048
A2	26.86165 ± 7.28263
LOGx0	1.15559 ± 0.11346
p	4.15241 ± 0
Reduced Chi-Sqr	8.35991
R-Square (COD)	0.95335
Adj. R-Square	0.90671

Model	DoseResp
Equation	y = A1 + (A2-A1)/(1 + 10^((LOGx0-x)*p))
Plot	**9**
A1	13.09078 ± 2.79912
A2	38.42355 ± 5.5778
LOGx0	1.00831 ± 0.10019
p	4.15241 ± 0
Reduced Chi-Sqr	20.91919
R-Square (COD)	0.91324
Adj. R-Square	0.82648

Model	DoseResp
Equation	y = A1 + (A2-A1)/(1 + 10^((LOGx0-x)*p))
Plot	**10**
A1	-0.54318 ± 0.46872
A2	77.4691 ± 2.88519
LOGx0	1.06366 ± 0.02026
p	1.65673 ± 0.07668
Reduced Chi-Sqr	0.07367
R-Square (COD)	0.99996
Adj. R-Square	0.99985

ORAC:

Model	DoseResp
Equation	y = A1 + (A2-A1)/(1 + 10^((LOGx0-x)*p))
Plot	**Ascorbic Acid**
A1	-1.09264 ± 1.19871
A2	95.7668 ± 2.62827
LOGx0	1.05572 ± 0.02964
p	1.66083 ± 0
Reduced Chi-Sqr	3.96171
R-Square (COD)	0.99871
Adj. R-Square	0.99742

Model	DoseResp
Equation	y = A1 + (A2-A1)/(1 + 10^((LOGx0-x)*p))
Plot	**Trolox**
A1	5.26536 ± 1.48624
A2	94.21687 ± 0.68365
LOGx0	-0.42967 ± 0.02468
p	1.48507 ± 0.07536
Reduced Chi-Sqr	0.79303
R-Square (COD)	0.99988
Adj. R-Square	0.99953

Model	DoseResp
Equation	y = A1 + (A2-A1)/(1 + 10^((LOGx0-x)*p))
Plot	**1**
A1	4.31651 ± 1.9997
A2	95.12397 ± 0.5747
LOGx0	-0.67198 ± 0.01365
p	1.93836 ± 0.24741
Reduced Chi-Sqr	0.62433
R-Square (COD)	0.9999
Adj. R-Square	0.99962

Model	DoseResp
Equation	y = A1 + (A2-A1)/(1 + 10^((LOGx0-x)*p))
Plot	**2**
A1	19.80786 ± 3.1091
A2	97.00412 ± 1.32119
LOGx0	-0.6496 ± 0.05072
p	1.66083 ± 0
Reduced Chi-Sqr	4.8278
R-Square (COD)	0.99786
Adj. R-Square	0.99572

Model	DoseResp
Equation	y = A1 + (A2-A1)/(1 + 10^((LOGx0-x)*p))
Plot	**3**
A1	-3.36328 ± 1.94472
A2	75.66205 ± 0.90819
LOGx0	-0.37542 ± 0.03823
p	1.43931 ± 0.10376
Reduced Chi-Sqr	1.3633
R-Square (COD)	0.99975
Adj. R-Square	0.99898

Model	DoseResp
Equation	y = A1 + (A2-A1)/(1 + 10^((LOGx0-x)*p))
Plot	**4**
A1	5.93003 ± 16.98083
A2	115.2496 ± 17.81421
LOGx0	0.22949 ± 0.23252
p	0.74436 ± 0.42521
Reduced Chi-Sqr	44.64196
R-Square (COD)	0.99357
Adj. R-Square	0.97428

Model	DoseResp
Equation	y = A1 + (A2-A1)/(1 + 10^((LOGx0-x)*p))
Plot	**5**
A1	-1.64732 ± 7.2333
A2	85.17782 ± 3.21765
LOGx0	-0.57515 ± 0.10557
p	1.87428 ± 0.87112
Reduced Chi-Sqr	19.39687
R-Square (COD)	0.99702
Adj. R-Square	0.98807

Model	DoseResp
Equation	y = A1 + (A2-A1)/(1 + 10^((LOGx0-x)*p))
Plot	**6**
A1	1.0064 ± 9.36415
A2	81.57581 ± 3.16817
LOGx0	-0.83196 ± 0.11191
p	1.66083 ± 0
Reduced Chi-Sqr	28.75109
R-Square (COD)	0.98547
Adj. R-Square	0.97095

Model	DoseResp
Equation	y = A1 + (A2-A1)/(1 + 10^((LOGx0-x)*p))
Plot	**7**
A1	-1.32988 ± 1.78154
A2	91.66557 ± 2.12947
LOGx0	0.24465 ± 0.03213
p	1.06084 ± 0.1124
Reduced Chi-Sqr	1.839
R-Square (COD)	0.99972
Adj. R-Square	0.99889

Model	DoseResp
Equation	y = A1 + (A2-A1)/(1 + 10^((LOGx0-x)*p))
Plot	**8**
A1	0.97046 ± 0.64392
A2	71.28556 ± 2.0384
LOGx0	1.26494 ± 0.03009
p	1.66083 ± 0
Reduced Chi-Sqr	1.17583
R-Square (COD)	0.99909
Adj. R-Square	0.99819

Model	DoseResp
Equation	y = A1 + (A2-A1)/(1 + 10^((LOGx0-x)*p))
Plot	**9**
A1	11.34148 ± 7.31694
A2	52.0487 ± 7.40405
LOGx0	0.1111 ± 0.3092
p	1.66083 ± 0
Reduced Chi-Sqr	96.88688
R-Square (COD)	0.88924
Adj. R-Square	0.77848

Model	DoseResp
Equation	y = A1 + (A2-A1)/(1 + 10^((LOGx0-x)*p))
Plot	**10**
A1	0.30782 ± 4.82964
A2	32.10597 ± 3.90861
LOGx0	-0.22003 ± 0.36714
p	1.66083 ± 0
Reduced Chi-Sqr	29.63542
R-Square (COD)	0.94244
Adj. R-Square	0.88488

3. Calculations

3.1. Cartesian coordinates of the optimized geometries

N,N-dimethyl-4-((2-(4-(trifluoromethyl)phenyl)hydrazineylidene)methyl)aniline (**2**)

0 1

C	-6.50837500	2.40690800	-0.01515300
N	-6.43083700	0.96768400	0.01540000
C	-7.65183700	0.20187100	-0.00237800
C	-5.21698100	0.33816700	0.00819400
C	-5.11929100	-1.06341600	0.01149500
C	-3.89551200	-1.69122200	0.00995300
C	-2.70341200	-0.96352000	0.00432800
C	-1.43410100	-1.63857100	0.00254200
N	-0.32685800	-1.00261200	-0.00273900
N	0.82097700	-1.68753700	-0.00402800
C	2.03649500	-1.06592300	-0.00950500
C	3.20919400	-1.82990100	-0.01165500
C	4.43987900	-1.21311900	-0.01723100
C	4.52110600	0.17606300	-0.02550900
C	5.86384100	0.83347300	-0.00009600
F	6.36603800	0.93584400	1.25239900
F	6.79125000	0.15966700	-0.70500800
F	5.85329600	2.08353000	-0.49076000
C	3.35995700	0.94402200	-0.01841600
C	2.12841900	0.33095300	-0.01300900
C	-2.79603100	0.43205600	0.00002000
C	-4.01357400	1.06684200	0.00156900
H	-7.55079700	2.71553800	-0.00647700
H	-6.04086700	2.81196000	-0.91845100
H	-6.01755100	2.84624900	0.85859000
H	-8.50482000	0.87585900	0.00723100
H	-7.72274700	-0.44661100	0.87626900
H	-7.71949700	-0.41986900	-0.90115100
H	-6.00799100	-1.67268200	0.01505200
H	-3.85831000	-2.77205900	0.01260300
H	-1.45556600	-2.73784800	0.00607800
H	3.14563700	-2.90818700	-0.00821100
H	5.34397900	-1.80255300	-0.02075100
H	3.43076800	2.02081700	-0.02250000
H	1.21773700	0.90604300	-0.01052000
H	-1.88349100	1.00668000	-0.00509800
H	-4.03084500	2.14410600	-0.00254200

N,N-dimethyl-4-((2-(3-(trifluoromethyl)phenyl)hydrazineylidene)methyl)aniline (**5**)

0 1

C	6.99516800	1.76469800	-0.08753500
N	6.63772600	0.37913600	0.14783200
C	7.65583600	-0.63533700	-0.03995700
C	5.30951300	0.00990600	0.08443000
C	4.92403900	-1.34413600	0.06753300
C	3.58413200	-1.69759100	0.03171100
C	2.57268200	-0.73744600	0.00231400
C	1.17344700	-1.15447900	-0.03726700
N	0.21062000	-0.31653700	-0.05460900
N	-1.04631900	-0.80518200	-0.10045400
C	-2.14437700	0.03318000	-0.05467900
C	-2.00558700	1.42626500	-0.00924100
C	-3.13488900	2.23072500	0.02174800
C	-4.41402600	1.68370900	0.01072900
C	-4.53744200	0.29976700	-0.03566000
C	-5.90090600	-0.32616100	0.01524300
F	-6.84290100	0.46624900	-0.51397900
F	-5.94778100	-1.49493300	-0.64415200
F	-6.28164100	-0.58653600	1.27906300
C	-3.42555900	-0.52816300	-0.06677700
C	2.95243600	0.60948500	0.00383100
C	4.28209700	0.97965400	0.03985100
H	6.67558700	2.11540800	-1.07774800
H	8.07596300	1.86818600	-0.01550800
H	6.54999200	2.41506300	0.66915300
H	8.63712100	-0.17096100	0.03385400
H	7.57559000	-1.13095600	-1.01674300
H	7.59128300	-1.39811600	0.73984700
H	5.66788100	-2.12797800	0.08182100
H	3.32088800	-2.75083900	0.02182200
H	0.97951100	-2.23404300	-0.05199200
H	-1.01306700	1.85417800	-0.00173900
H	-3.01360400	3.30683900	0.05338200
H	-5.29405100	2.31253000	0.02921600
H	-3.55201000	-1.60436700	-0.11029300
H	2.18240300	1.37116500	-0.02901800
H	4.52652300	2.03260200	0.03240100

N,N-dimethyl-4-((2-(2-(trifluoromethyl)phenyl)hydrazineylidene)methyl)aniline (**6**)

0 1

C	-7.09244800	-0.87905200	0.00593300
N	-6.15599300	0.22472800	-0.00517300
C	-6.63836700	1.59000700	0.00645000
C	-4.80531200	-0.02638000	-0.00365400
C	-4.30423000	-1.34341400	-0.00268300
C	-2.93867500	-1.57841700	-0.00228300
C	-2.01376400	-0.53366000	-0.00242700
C	-0.58403400	-0.82905800	-0.00180300
N	0.30384700	0.08828800	-0.00143200
N	1.59885500	-0.30464400	-0.00093200
C	2.61674500	0.62362100	-0.00036900
C	2.33485000	1.99799300	-0.00084900
C	3.36503700	2.92254300	-0.00037500
C	4.69912800	2.52146700	0.00058600
C	4.98825200	1.16565100	0.00109500
C	3.96779900	0.22010400	0.00064000
C	4.31132900	-1.23502200	0.00126000
F	3.80327500	-1.87999200	-1.07332100
F	5.62616700	-1.46201600	0.00223600
F	3.80178000	-1.87941700	1.07547800
C	-2.50844100	0.77583900	-0.00288200
C	-3.86510000	1.03041300	-0.00329300
H	-6.97278200	-1.51592800	-0.87757700
H	-8.10688300	-0.48634600	0.00469000
H	-6.96905300	-1.50320800	0.89828800
H	-6.30193500	2.12947400	0.89892000
H	-7.72616300	1.58290100	0.00559700
H	-6.30144700	2.14330000	-0.87703100
H	-4.97916700	-2.18770600	-0.00222000
H	-2.58398600	-2.60452300	-0.00157000
H	-0.29841300	-1.88784900	-0.00174800
H	1.30123700	2.31178800	-0.00160500
H	3.11925600	3.97819500	-0.00077600
H	5.49834100	3.25052700	0.00094000
H	6.01553500	0.82463100	0.00186800
H	-1.80600000	1.60098900	-0.00268400
H	-4.20115800	2.05804900	-0.00342300

1-(2,4-dinitrophenyl)-2-(phenyl(4-(trifluoromethyl)phenyl) methylene)hydrazine (**8**)

0 1

O	5.69979657	-4.00268905	-0.15127806
N	5.89399054	-2.80923696	-0.05283305
C	4.71439341	-1.93712993	-0.03506105
C	4.88974638	-0.57821183	0.06876495
C	3.77097428	0.24640119	0.08191396
N	4.03294324	1.68158230	0.18494496
O	3.07832316	2.44677033	0.12979496
O	5.17664532	2.04787637	0.31360997
C	2.45509819	-0.27980588	-0.00503205
N	1.34062509	0.49835714	0.01038195
N	0.11520302	-0.07577394	-0.03798505
C	-0.91145108	0.69658208	-0.04425905
C	-0.82903912	2.18605419	-0.04721105
C	-0.22113710	2.86064826	-1.10842213
C	-0.13427313	4.24819336	-1.09543913
C	-0.65016519	4.96748440	-0.02279505
C	-1.26035322	4.29988133	1.03518102
C	-1.35542918	2.91365622	1.02220302
C	-2.24417915	0.04584999	-0.03232405
C	-3.37751226	0.75250001	-0.44118508
C	-4.62456033	0.13909493	-0.45197408
C	-4.74156030	-1.18328318	-0.04656405
C	-6.09240640	-1.83716226	0.00561095
F	-6.63914640	-1.74191427	1.22975704
F	-6.95726046	-1.27767825	-0.85043411
F	-6.02627035	-3.14373135	-0.28574407
C	-3.62109219	-1.89861419	0.37115198
C	-2.37989712	-1.28615111	0.37755498
C	2.33536723	-1.68744899	-0.11509206
C	3.43987034	-2.50117502	-0.12969706
O	6.98317057	-2.28502589	0.03216695
H	5.87905541	-0.14756377	0.13899896
H	1.45032807	1.50506421	0.08985396
H	0.18132595	2.29630723	-1.94324319
H	0.34012189	4.76508641	-1.92044219
H	-0.57590522	6.04808145	-0.01022005
H	-1.66167426	4.85915936	1.87154209
H	-1.83591220	2.38903118	1.84089708
H	-3.28476528	1.78236508	-0.76411710
H	-5.49985239	0.68468294	-0.78166810
H	-3.72590417	-2.92913727	0.68830300
H	-1.50236304	-1.82844112	0.70492900
H	1.34242517	-2.10379305	-0.19155306
H	3.34003236	-3.57506309	-0.21535207

1-(anthracen-9-ylmethylene)-2-(3-(trifluoromethyl)phenyl)hydrazine (**10**)

0 1

F	7.02178200	-0.43153000	-0.70594100
C	6.07122900	-0.55251100	0.23020400
C	4.74942800	-0.01008800	-0.23160600
C	3.58451200	-0.54879800	0.29400800
C	2.34876400	-0.01620600	-0.08197800
N	1.19331200	-0.56495100	0.45443500
N	-0.01908200	-0.10891700	0.09802000
C	-1.04630600	-0.68489000	0.59081700
C	-2.42136000	-0.24767900	0.29149900
C	-3.39264400	-1.24649200	0.05786200
C	-3.07081800	-2.64180300	0.00291500
C	-4.03197400	-3.58274000	-0.21621400
C	-5.39156600	-3.20596900	-0.41020600
C	-5.73706800	-1.89052500	-0.39553500
C	-4.75698600	-0.87360100	-0.17176200
C	-5.10337400	0.47487100	-0.18771000
C	-4.15581200	1.47214700	0.02848800
C	-2.78799500	1.11750600	0.28731000
C	-1.87209700	2.18451500	0.56632200
C	-2.27814600	3.48495900	0.55325700
C	-3.62810800	3.83122700	0.25963200
C	-4.53662400	2.85119900	0.01173400
C	2.30189300	1.05458600	-0.98194500
C	3.48254100	1.57505400	-1.49146300
C	4.71928800	1.05442800	-1.12488800
F	5.99887500	-1.85175100	0.55972800
F	6.51875600	0.09447400	1.32091500
H	3.63589500	-1.38280100	0.98517600
H	1.26748300	-1.36622600	1.07218200
H	-0.92677900	-1.54094400	1.26366200
H	-2.04192700	-2.95933200	0.11045500
H	-3.75969400	-4.63071500	-0.25675600
H	-6.14045900	-3.96958100	-0.58148700
H	-6.76468300	-1.58421200	-0.55862300
H	-6.13578800	0.75570800	-0.37239000
H	-0.84165600	1.95024400	0.78682200
H	-1.56469100	4.27019300	0.77373900
H	-3.92512500	4.87294600	0.24631200
H	-5.57273000	3.09275600	-0.19917000
H	1.34104800	1.45606700	-1.27395300
H	3.43511600	2.40028300	-2.19160900
H	5.63901100	1.45682500	-1.52828800

3.2. Mulliken charges

N,N-dimethyl-4-((2-(4-(trifluoromethyl)phenyl)hydrazineylidene)methyl)aniline (**2**)

1	C	-0.365766
2	N	0.083980
3	C	-0.380485
4	C	-0.912901
5	C	-0.257376
6	C	-0.651855
7	C	0.508794
8	C	0.007765
9	N	0.433158
10	N	-0.222167
11	C	-1.182459
12	C	-0.003537
13	C	0.024887
14	C	-0.516463
15	C	0.775238
16	F	-0.065129
17	F	-0.106733
18	F	-0.124842
19	C	-0.659389
20	C	0.913253
21	C	0.198326
22	C	-0.113836
23	H	0.158917
24	H	0.171624
25	H	0.169518
26	H	0.160617
27	H	0.168098
28	H	0.170607
29	H	0.147097
30	H	0.149552
31	H	0.093605
32	H	0.238818
33	H	0.139600
34	H	0.177986
35	H	0.174456
36	H	0.180827
37	H	0.168222
38	H	0.147994

N,N-dimethyl-4-((2-(4-(trifluoromethyl)phenyl)hydrazineylidene)methyl)aniline radical (with spin density)

1	C	-0.362957	-0.003338
2	N	0.087694	0.048727
3	C	-0.375589	-0.003587
4	C	-0.919941	0.085266
5	C	-0.204736	-0.033939
6	C	-0.542908	0.097431
7	C	0.694867	-0.060173
8	C	-0.040387	0.339159
9	N	0.052300	-0.186243
10	N	0.125464	0.522955
11	C	-1.033179	-0.150924
12	C	0.197110	0.160830
13	C	-0.012517	-0.056675
14	C	-0.478169	0.145943
15	C	0.692792	0.009482
16	F	-0.058650	0.000964
17	F	-0.113532	0.000226
18	F	-0.103042	0.000713
19	C	-0.616584	-0.067964
20	C	0.550320	0.141963
21	C	0.052923	0.064421
22	C	-0.124180	-0.044823
23	H	0.163575	0.000139
24	H	0.176565	0.004058
25	H	0.176531	0.004063
26	H	0.164908	0.000130
27	H	0.175532	0.004071
28	H	0.175494	0.004067
29	H	0.152877	0.000937
30	H	0.162015	-0.004210
31	H	0.148145	-0.012469
32	H	0.168184	-0.006651
33	H	0.178796	0.002401
34	H	0.176667	0.002382
35	H	0.182324	-0.006903
36	H	0.178874	-0.003525
37	H	0.152413	0.001098

N,N-dimethyl-4-((2-(3-(trifluoromethyl)phenyl)hydrazineylidene)methyl)aniline (**5**)

1	C	-0.391190
2	N	0.096968
3	C	-0.401270
4	C	-0.880248
5	C	-0.183113
6	C	-0.710882
7	C	0.485118
8	C	-0.072746
9	N	0.412828
10	N	-0.236905
11	C	-0.571974
12	C	1.084137
13	C	-0.638782
14	C	-0.039284
15	C	-0.813300
16	C	0.524211
17	F	-0.110265
18	F	-0.109454
19	F	-0.058213
20	C	-0.178777
21	C	0.266829
22	C	-0.106373
23	H	0.178735
24	H	0.158773
25	H	0.164562
26	H	0.161181
27	H	0.178134
28	H	0.163257
29	H	0.147523
30	H	0.150652
31	H	0.095241
32	H	0.240561
33	H	0.181887
34	H	0.162047
35	H	0.174021
36	H	0.159700
37	H	0.168815
38	H	0.147596

N,N-dimethyl-4-((2-(3-(trifluoromethyl)phenyl)hydrazineylidene)methyl)aniline radical (with spin density)

1	C	-0.361854	-0.003262
2	N	0.088540	0.047490
3	C	-0.377053	-0.003511
4	C	-0.915624	0.084695
5	C	-0.206733	-0.033175
6	C	-0.581396	0.096806
7	C	0.523275	-0.061742
8	C	-0.005403	0.342385
9	N	0.066391	-0.188119
10	N	0.100082	0.524493
11	C	-0.661599	-0.143292
12	C	0.649410	0.152291
13	C	-0.681230	-0.074909
14	C	0.408985	0.162464
15	C	-0.816608	-0.064456
16	C	0.583431	0.011486
17	F	-0.095138	0.000139
18	F	-0.120122	0.000093
19	F	-0.056334	0.000106
20	C	-0.166590	0.149280
21	C	0.197269	0.068887
22	C	-0.103546	-0.047877
23	H	0.175730	0.003987
24	H	0.162430	0.000137
25	H	0.175921	0.003991
26	H	0.165240	0.000124
27	H	0.175321	0.003992
28	H	0.175528	0.004003
29	H	0.152679	0.000975
30	H	0.162103	-0.004230
31	H	0.148736	-0.012573
32	H	0.182008	-0.007151
33	H	0.163818	0.002557
34	H	0.179713	-0.007864
35	H	0.183775	-0.005761
36	H	0.177051	-0.003578
37	H	0.151793	0.001118

N,N-dimethyl-4-((2-(2-(trifluoromethyl)phenyl)hydrazineylidene)methyl)aniline (**6**)

1	C	-0.374043
2	N	0.078279
3	C	-0.370775
4	C	-0.877226
5	C	-0.081621
6	C	-0.727995
7	C	0.704522
8	C	-0.200946
9	N	0.264843
10	N	-0.047884
11	C	-0.355176
12	C	0.356399
13	C	-0.314589
14	C	-0.419369
15	C	-0.282755
16	C	0.098516
17	C	0.217716
18	F	-0.073961
19	F	-0.142103
20	F	-0.074056
21	C	0.083524
22	C	-0.106488
23	H	0.169552
24	H	0.160685
25	H	0.170219
26	H	0.170905
27	H	0.159225
28	H	0.170223
29	H	0.148453
30	H	0.151240
31	H	0.102885
32	H	0.253461
33	H	0.183807
34	H	0.160738
35	H	0.156709
36	H	0.172333
37	H	0.166052
38	H	0.148703

N,N-dimethyl-4-((2-(2-(trifluoromethyl)phenyl)hydrazineylidene)methyl)aniline radical (with spin density)

1	C	-0.373793	-0.003430
2	N	0.086144	0.047221
3	C	-0.368537	-0.003291
4	C	-0.897829	0.083636
5	C	-0.079806	-0.030882
6	C	-0.639319	0.093947
7	C	0.640186	-0.053700
8	C	-0.014420	0.329972
9	N	0.139182	-0.203132
10	N	0.066554	0.541092
11	C	-0.179827	-0.134965
12	C	0.142285	0.180189
13	C	-0.478814	-0.080299
14	C	-0.360697	0.164034
15	C	-0.314128	-0.091705
16	C	0.193640	0.151873
17	C	0.303924	-0.000142
18	F	-0.062421	0.001359
19	F	-0.167245	0.000172
20	F	-0.062457	0.001364
21	C	0.047285	0.066155
22	C	-0.135591	-0.047451
23	H	0.175186	0.003912
24	H	0.164783	0.000124
25	H	0.176155	0.004002
26	H	0.176444	0.003995
27	H	0.163019	0.000134
28	H	0.175526	0.003906
29	H	0.153104	0.000937
30	H	0.163176	-0.004119
31	H	0.156278	-0.012031
32	H	0.182844	-0.007438
33	H	0.162150	0.002760
34	H	0.162389	-0.008324
35	H	0.176136	0.002541
36	H	0.176221	-0.003496
37	H	0.152272	0.001082

1-(2,4-dinitrophenyl)-2-(phenyl(4-(trifluoromethyl)phenyl) methylene)hydrazine (**8**)

1	F	-0.045838
2	C	1.195373
3	C	-1.148130
4	C	-0.204210
5	C	-0.036660
6	C	0.605636
7	C	-0.394472
8	C	0.182421
9	C	-0.403730
10	C	-0.411970
11	C	-0.053610
12	C	-0.268290
13	C	0.220748
14	N	0.476245
15	N	-0.230882
16	C	-0.342617
17	C	-0.030556
18	N	-0.320123
19	O	0.065229
20	O	-0.015326
21	C	-0.103297
22	C	-0.010816
23	N	-0.261101
24	O	0.008056
25	O	0.013593
26	C	-0.591030
27	C	0.646640
28	C	-0.538336
29	C	-0.369417
30	F	-0.090074
31	F	-0.108098
32	H	0.182748
33	H	0.182261
34	H	0.166941
35	H	0.168947
36	H	0.169300
37	H	0.165501
38	H	0.178694
39	H	0.343563
40	H	0.233473
41	H	0.200612
42	H	0.199249
43	H	0.190613
44	H	0.182742

1-(2,4-dinitrophenyl)-2-(phenyl(4-(trifluoromethyl)phenyl) methylene)hydrazine radical (with spin density)

1	F	-0.042303	0.000094
2	C	0.990254	0.003951
3	C	-0.827244	0.048227
4	C	-0.378071	0.002120
5	C	0.073369	0.074781
6	C	0.318774	-0.115289
7	C	-0.321060	0.330270
8	C	0.862974	-0.082811
9	C	-0.767495	0.052134
10	C	-0.487405	-0.018507
11	C	-0.090486	0.049183
12	C	-0.431494	-0.001843
13	C	0.502729	0.080071
14	N	0.363449	-0.135416
15	N	0.017443	0.513935
16	C	-0.098355	-0.164626
17	C	-0.012099	0.160015
18	N	-0.384106	-0.011783
19	O	0.074223	0.001149
20	O	0.107088	0.014066
21	C	0.038598	-0.063202
22	C	-0.161817	0.139917
23	N	-0.223854	-0.015146
24	O	0.010011	0.013071
25	O	0.012929	0.016776
26	C	-0.702598	-0.079418
27	C	0.352151	0.168188
28	C	-0.035791	0.053663
29	C	-0.819728	-0.024934
30	F	-0.091170	0.000337
31	F	-0.099488	0.000073
32	H	0.184840	0.000854
33	H	0.183842	-0.001449
34	H	0.178861	-0.002091
35	H	0.165037	0.001146
36	H	0.169094	-0.002395
37	H	0.168852	0.000947
38	H	0.190598	-0.001607
39	H	0.223612	0.002202
40	H	0.204586	0.002362
41	H	0.200994	-0.006995
42	H	0.194686	-0.003083
43	H	0.185571	0.001064

1-(anthracen-9-ylmethylene)-2-(3-(trifluoromethyl)phenyl)hydrazine (**10**)

1	F	-0.105481
2	C	0.583906
3	C	-0.769730
4	C	-0.056040
5	C	-0.648802
6	N	-0.331697
7	N	0.544409
8	C	-1.065034
9	C	0.735655
10	C	0.272453
11	C	-0.330690
12	C	-0.705817
13	C	-0.373887
14	C	-0.230695
15	C	0.356903
16	C	0.091214
17	C	0.328284
18	C	0.419734
19	C	-0.345991
20	C	-0.517403
21	C	-0.343421
22	C	-0.114365
23	C	0.974064
24	C	-0.662912
25	C	-0.084244
26	F	-0.109539
27	F	-0.055938
28	H	0.160223
29	H	0.243280
30	H	0.126244
31	H	0.165897
32	H	0.158289
33	H	0.160997
34	H	0.159927
35	H	0.164513
36	H	0.194097
37	H	0.156682
38	H	0.160349
39	H	0.156176
40	H	0.196604
41	H	0.165335
42	H	0.176454

1-(anthracen-9-ylmethylene)-2-(3-(trifluoromethyl)phenyl)hydrazine radical (with spin density)

1	F	-0.094132	0.000194
2	C	0.642072	0.009861
3	C	-0.731219	-0.061398
4	C	-0.041046	0.141154
5	C	-0.930898	-0.117829
6	N	0.191823	0.477073
7	N	0.040635	-0.205487
8	C	-0.467906	0.346404
9	C	0.393253	-0.148203
10	C	0.518901	0.130176
11	C	-0.522353	-0.048469
12	C	-0.546474	0.059210
13	C	-0.456588	-0.038953
14	C	-0.199144	0.070043
15	C	0.234824	-0.089972
16	C	-0.138100	0.214947
17	C	0.015071	-0.094819
18	C	0.518539	0.092017
19	C	-0.181032	-0.044699
20	C	-0.347759	0.103117
21	C	-0.315431	-0.037344
22	C	-0.170307	0.072143
23	C	0.613090	0.129717
24	C	-0.610827	-0.066288
25	C	0.364701	0.148127
26	F	-0.116359	0.000050
27	F	-0.053640	0.000196
28	H	0.184822	-0.005450
29	H	0.152701	-0.014970
30	H	0.170959	0.001475
31	H	0.162246	-0.003266
32	H	0.163507	0.001767
33	H	0.160090	-0.002368
34	H	0.169781	-0.008427
35	H	0.203903	0.006069
36	H	0.160644	-0.003401
37	H	0.162514	0.001756
38	H	0.158823	-0.002475
39	H	0.189733	-0.006764
40	H	0.167760	0.002322
41	H	0.182824	-0.007236

3.3. Fukui function values

N,N-dimethyl-4-((2-(4-(trifluoromethyl)phenyl)hydrazineylidene)methyl)aniline (**2**)

Atom index	OW f+	OW f-	OW f0	OW DD
1(C)	0.01816	0.01531	0.01673	0.00285
2(N)	0.00754	0.11516	0.06135	-0.10761
3(C)	0.01855	0.01527	0.01691	0.00328
4(C)	0.01835	0.05246	0.03541	-0.03410
5(C)	0.01964	0.06132	0.04048	-0.04168
6(C)	0.02546	0.04049	0.03298	-0.01504
7(C)	0.01778	0.07814	0.04796	-0.06036
8(C)	0.03397	0.05244	0.04321	-0.01847
9(N)	0.02431	0.06329	0.04380	-0.03899
10(N)	0.01286	0.10408	0.05847	-0.09122
11(C)	0.02239	0.03519	0.02879	-0.01280
12(C)	0.02617	0.04448	0.03532	-0.01831
13(C)	0.02400	0.02119	0.02259	0.00281
14(C)	0.01632	0.05137	0.03385	-0.03506
15(C)	0.01380	0.00632	0.01006	0.00748
16(F)	0.00647	0.00354	0.00500	0.00293
17(F)	0.00625	0.00167	0.00396	0.00458
18(F)	0.00607	0.00121	0.00364	0.00486
19(C)	0.02305	0.02089	0.02197	0.00216
20(C)	0.02256	0.04572	0.03414	-0.02316
21(C)	0.02497	0.04014	0.03255	-0.01516
22(C)	0.01959	0.05391	0.03675	-0.03432
23(H)	0.02652	0.00096	0.01374	0.02557
24(H)	0.02076	0.00870	0.01473	0.01206
25(H)	0.02075	0.00827	0.01451	0.01248
26(H)	0.02671	0.00095	0.01383	0.02577
27(H)	0.02146	0.00827	0.01487	0.01319
28(H)	0.02149	0.00874	0.01511	0.01275
29(H)	0.01862	0.00595	0.01228	0.01267
30(H)	0.02754	0.00296	0.01525	0.02458
31(H)	0.02975	0.00400	0.01687	0.02575
32(H)	0.03280	0.00874	0.02077	0.02407
33(H)	0.03060	0.00362	0.01711	0.02698
34(H)	0.02171	0.00143	0.01157	0.02028
35(H)	0.02009	0.00143	0.01076	0.01866
36(H)	0.01580	0.00435	0.01008	0.01145
37(H)	0.01711	0.00284	0.00998	0.01427
38(H)	0.01718	0.00514	0.01116	0.01204

N,N-dimethyl-4-((2-(3-(trifluoromethyl)phenyl)hydrazineylidene)methyl)aniline (**5**)

Atom index	OW f+	OW f-	OW f0	OW DD
1(C)	0.01753	0.01496	0.01625	0.00257
2(N)	0.00732	0.11447	0.06089	-0.10715
3(C)	0.01773	0.01492	0.01633	0.00281
4(C)	0.01828	0.05299	0.03564	-0.03471
5(C)	0.01998	0.06077	0.04038	-0.04079
6(C)	0.02536	0.04113	0.03325	-0.01577
7(C)	0.01789	0.07512	0.04651	-0.05724
8(C)	0.03286	0.05352	0.04319	-0.02066
9(N)	0.02410	0.06000	0.04205	-0.03590
10(N)	0.01207	0.10987	0.06097	-0.09780
11(C)	0.02114	0.03773	0.02943	-0.01659
12(C)	0.02533	0.04406	0.03470	-0.01873
13(C)	0.02535	0.02288	0.02411	0.00247
14(C)	0.02148	0.05330	0.03739	-0.03182
15(C)	0.02031	0.02187	0.02109	-0.00156
16(C)	0.01642	0.00201	0.00922	0.01441
17(F)	0.00689	0.00061	0.00375	0.00628
18(F)	0.00742	0.00042	0.00392	0.00700
19(F)	0.00767	0.00099	0.00433	0.00668
20(C)	0.02595	0.04626	0.03610	-0.02031
21(C)	0.02509	0.04033	0.03271	-0.01523
22(C)	0.02000	0.05363	0.03682	-0.03363
23(H)	0.01989	0.00963	0.01476	0.01026
24(H)	0.02550	0.00103	0.01326	0.02447
25(H)	0.02046	0.00613	0.01329	0.01434
26(H)	0.02569	0.00103	0.01336	0.02465
27(H)	0.02026	0.00970	0.01498	0.01056
28(H)	0.02085	0.00610	0.01347	0.01475
29(H)	0.01853	0.00575	0.01214	0.01278
30(H)	0.02686	0.00301	0.01494	0.02385
31(H)	0.02807	0.00407	0.01607	0.02401
32(H)	0.03053	0.00930	0.01992	0.02124
33(H)	0.01717	0.00406	0.01061	0.01311
34(H)	0.02604	0.00170	0.01387	0.02434
35(H)	0.02043	0.00514	0.01279	0.01529
36(H)	0.02446	0.00364	0.01405	0.02082
37(H)	0.01814	0.00286	0.01050	0.01528
38(H)	0.01738	0.00497	0.01118	0.01241

N,N-dimethyl-4-((2-(2-(trifluoromethyl)phenyl)hydrazineylidene)methyl)aniline (**6**)

Atom index	OW f+	OW f-	OW f0	OW DD
1(C)	0.01740	0.01509	0.01624	0.00231
2(N)	0.00688	0.11553	0.06120	-0.10865
3(C)	0.01731	0.01515	0.01623	0.00216
4(C)	0.01690	0.05210	0.03450	-0.03520
5(C)	0.01838	0.06229	0.04033	-0.04391
6(C)	0.02317	0.04037	0.03177	-0.01720
7(C)	0.01615	0.07699	0.04657	-0.06084
8(C)	0.02938	0.05063	0.04001	-0.02125
9(N)	0.02146	0.06057	0.04102	-0.03911
10(N)	0.00893	0.10527	0.05710	-0.09633
11(C)	0.01892	0.03530	0.02711	-0.01637
12(C)	0.02148	0.04698	0.03423	-0.02549
13(C)	0.02526	0.02122	0.02324	0.00405
14(C)	0.02099	0.05547	0.03823	-0.03449
15(C)	0.02275	0.02470	0.02373	-0.00195
16(C)	0.01757	0.04113	0.02935	-0.02356
17(C)	0.01703	0.00454	0.01079	0.01249
18(F)	0.00855	0.00193	0.00524	0.00662
19(F)	0.00767	0.00041	0.00404	0.00726
20(F)	0.00855	0.00193	0.00524	0.00662
21(C)	0.02329	0.03954	0.03142	-0.01625
22(C)	0.01861	0.05484	0.03673	-0.03623
23(H)	0.02003	0.00828	0.01415	0.01175
24(H)	0.02528	0.00094	0.01311	0.02434
25(H)	0.01999	0.00845	0.01422	0.01154
26(H)	0.01984	0.00842	0.01413	0.01142
27(H)	0.02517	0.00095	0.01306	0.02422
28(H)	0.01987	0.00825	0.01406	0.01162
29(H)	0.01715	0.00600	0.01158	0.01115
30(H)	0.02382	0.00294	0.01338	0.02088
31(H)	0.02267	0.00383	0.01325	0.01884
32(H)	0.01333	0.00865	0.01099	0.00467
33(H)	0.01520	0.00445	0.00982	0.01075
34(H)	0.02490	0.00151	0.01321	0.02340
35(H)	0.02427	0.00543	0.01485	0.01884
36(H)	0.02037	0.00187	0.01112	0.01851
37(H)	0.01701	0.00278	0.00989	0.01422
38(H)	0.01648	0.00519	0.01084	0.01128

1-(2,4-dinitrophenyl)-2-(phenyl(4-(trifluoromethyl)phenyl) methylene)hydrazine (**8**)

Atom index	OW f+	OW f-	OW f0	OW DD
1(F)	0.00347	0.00239	0.00293	0.00108
2(C)	0.00726	0.00382	0.00554	0.00344
3(C)	0.02024	0.03699	0.02861	-0.01675
4(C)	0.01718	0.03164	0.02441	-0.01446
5(C)	0.02184	0.04109	0.03147	-0.01925
6(C)	0.02175	0.03809	0.02992	-0.01633
7(C)	0.02938	0.04899	0.03918	-0.01961
8(C)	0.01499	0.03895	0.02697	-0.02396
9(C)	0.01659	0.03849	0.02754	-0.0219
10(C)	0.01567	0.03599	0.02583	-0.02032
11(C)	0.01671	0.03702	0.02687	-0.02031
12(C)	0.01494	0.03647	0.02571	-0.02154
13(C)	0.01585	0.03715	0.0265	-0.0213
14(N)	0.02684	0.05588	0.04136	-0.02904
15(N)	0.01529	0.09116	0.05323	-0.07587
16(C)	0.03657	0.02827	0.03242	0.0083
17(C)	0.0277	0.04111	0.0344	-0.01341
18(N)	0.05301	0.00767	0.03034	0.04534
19(O)	0.04398	0.01825	0.03112	0.02572
20(O)	0.04191	0.01466	0.02829	0.02725
21(C)	0.04172	0.02301	0.03237	0.01871
22(C)	0.02572	0.0481	0.03691	-0.02238
23(N)	0.03762	0.0093	0.02346	0.02832
24(O)	0.03202	0.02397	0.02799	0.00805
25(O)	0.03108	0.02513	0.0281	0.00594
26(C)	0.03805	0.02019	0.02912	0.01786
27(C)	0.02633	0.04519	0.03576	-0.01886
28(C)	0.02052	0.04055	0.03054	-0.02004

1-(anthracen-9-ylmethylene)-2-(3-(trifluoromethyl)phenyl)hydrazine (**10**)

tom index	OW f+	OW f-	OW f0	OW DD
1(F)	0.0043	0.0010	0.0027	0.0033
2(C)	0.0092	0.0019	0.0056	0.0074
3(C)	0.0140	0.0203	0.0172	-0.0063
4(C)	0.0177	0.0381	0.0279	-0.0203
5(C)	0.0147	0.0321	0.0234	-0.0173
6(N)	0.0094	0.0776	0.0435	-0.0682
7(N)	0.0186	0.0345	0.0266	-0.0159
8(C)	0.0215	0.0436	0.0325	-0.0221
9(C)	0.0248	0.0639	0.0444	-0.0391
10(C)	0.0165	0.0354	0.0259	-0.0189
11(C)	0.0180	0.0464	0.0322	-0.0284
12(C)	0.0201	0.0415	0.0308	-0.0214
13(C)	0.0190	0.0393	0.0291	-0.0203
14(C)	0.0193	0.0451	0.0322	-0.0258
15(C)	0.0147	0.0321	0.0234	-0.0174
16(C)	0.0285	0.0627	0.0456	-0.0342
17(C)	0.0148	0.0316	0.0232	-0.0168
18(C)	0.0162	0.0354	0.0258	-0.0191
19(C)	0.0210	0.0414	0.0312	-0.0204
20(C)	0.0224	0.0372	0.0298	-0.0148
21(C)	0.0201	0.0369	0.0285	-0.0168
22(C)	0.0213	0.0405	0.0309	-0.0193
23(C)	0.0173	0.0367	0.0270	-0.0194
24(C)	0.0167	0.0214	0.0191	-0.0047
25(C)	0.0143	0.0433	0.0288	-0.0290
26(F)	0.0038	0.0006	0.0022	0.0032
27(F)	0.0042	0.0004	0.0023	0.0037
28(H)	0.0156	0.0030	0.0093	0.0127
29(H)	0.0210	0.0067	0.0139	0.0143
30(H)	0.0154	0.0052	0.0103	0.0102
31(H)	0.0077	0.0049	0.0063	0.0029
32(H)	0.0151	0.0038	0.0094	0.0113
33(H)	0.0148	0.0035	0.0091	0.0113
34(H)	0.0137	0.0039	0.0088	0.0098
35(H)	0.0136	0.0053	0.0095	0.0083
36(H)	0.0148	0.0043	0.0095	0.0105
37(H)	0.0169	0.0034	0.0101	0.0135
38(H)	0.0158	0.0033	0.0095	0.0125

39(H)	0.0147	0.0035	0.0091	0.0112
40(H)	0.0091	0.0033	0.0062	0.0058
41(H)	0.0149	0.0016	0.0083	0.0133
42(H)	0.0116	0.0041	0.0078	0.0075

3.4. EDDB

To calculate EDDB[2]:
RunEDDB.R -i filename.fchk filename.49 -f {fragment coordinate, e.g., 11-20, 22} -d {π NOBDs} -od -o EDDBG_PI.fchk > EDDBG_PI.out

```
o Parsing cpm2rad.fchk and CPM2RAD.49... DONE!

 __________________ELECTRON_DENSITY_OF_DELOCALIZED_BONDS___________________
|                                                                           |
|  1) for the entire molecular system (including hydrogens)......EDDB_G(r) |
|  2) for the entire molecular system (excluding hydrogens)......EDDB_H(r) |
|  3) for the molecular fragment (excluding non-local effects)...EDDB_F(r) |
|  4) for the molecular fragment (including non-local effects)...EDDB_E(r) |
|  5) for the particular delocalization pathway..................EDDB_P(r) |
|  0) skip the EDDB calculations and perform the standard NPA analysis.     |
|___________________________________________________________________________|

o Select type of the EDDB function and press <Enter>:
  > 3
o Define molecular fragment ( e.g. 1:6,9,10 ) and press <Enter>
  > 10,11,12,13,14,15,16,17,18,19,20
o Calculation of the EDDB_F(r) function in the NAO basis... DONE!
o List of non-zero eigenvalues of the EDDB_F(r) function:

 NOBD ___1__ ___2__ ___3__ ___4__ ___5__ ___6__ ___7__ ___8__ ___9__ __10__

 1     1.6533 1.6132 1.2726 0.1881 0.1512 0.1361 0.1262 0.1014 0.0994 0.0956
 11    0.0852 0.0766 0.0749 0.0739 0.0583 0.0520 0.0484 0.0457 0.0432 0.0394
 21    0.0364 0.0201 0.0138 0.0129 0.0121 0.0117 0.0089 0.0088 0.0068 0.0039
 31    0.0029 0.0011 0.0003
 ___________________________________________________________________________

o Enter the list of spinless NOBDs to dissect and/or press <Enter>
  > 1:3
o Calculation of the dissected EDDB_F(r) function... DONE!
o Saving dissected EDDB_F(r) function to EDDBG_p_2r_CF3_PI.fchk... DONE!
o Results of the NPA and EDDB_F population analyses in the NAO basis:

 ___________________________________________________________________________
|        ::Total::          |       ::Alpha Spin::      |       ::Beta Spin::       |
|  Atom     NPA    EDDB_F   |  Atom     NPA    EDDB_F   |  Atom     NPA    EDDB_F   |
| ''''''  ''''''  ''''''    | ''''''  ''''''  ''''''    | ''''''  ''''''  ''''''    |
| 10  N   0.0484  0.0321    | 10  N   0.0249  0.0174    | 10  N   0.0236  0.0147    |
| 11  C   0.9142  0.6020    | 11  C   0.4693  0.3281    | 11  C   0.4449  0.2738    |
| 12  C   0.9661  0.7314    | 12  C   0.4882  0.3776    | 12  C   0.4779  0.3538    |
| 13  C   0.9778  0.8076    | 13  C   0.4890  0.4002    | 13  C   0.4888  0.4074    |
| 14  C   1.0507  0.8210    | 14  C   0.5295  0.4107    | 14  C   0.5212  0.4103    |
| 15  C   0.0257  0.0193    | 15  C   0.0130  0.0098    | 15  C   0.0127  0.0095    |
| 16  F   0.0051  0.0039    | 16  F   0.0026  0.0019    | 16  F   0.0025  0.0019    |
| 17  F   0.0015  0.0012    | 17  F   0.0008  0.0006    | 17  F   0.0008  0.0006    |
| 18  F   0.0021  0.0016    | 18  F   0.0011  0.0008    | 18  F   0.0010  0.0008    |
| 19  C   0.9758  0.7891    | 19  C   0.4881  0.3972    | 19  C   0.4877  0.3920    |
| 20  C   0.9692  0.7300    | 20  C   0.4891  0.3802    | 20  C   0.4801  0.3498    |
|___________________________|___________________________|___________________________|
|                                                                           |
| Total population of electrons  (from NPA):    5.9366e ( 0.5397e / atom ) |
| +                                Alpha spin:   2.9955e ( 0.2723e / atom ) |
| +                                 Beta spin:   2.9412e ( 0.2674e / atom ) |
| Total population of delocalized electrons:    4.5391e ( 0.4126e / atom ) |
| +                                Alpha spin:   2.3245e ( 0.2113e / atom ) |
| +                                 Beta spin:   2.2146e ( 0.2013e / atom ) |
|___________________________________________________________________________|
```

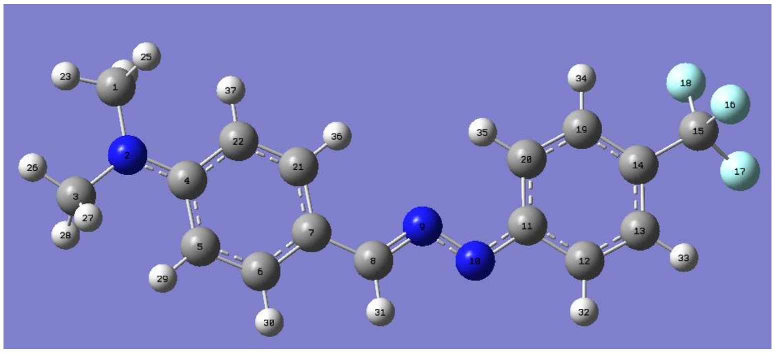

Figure S41. Example EDDBF output for compound 2 with corresponding labeled ball-and-stick representation used to identify the analyzed fragment.

Table S1. Summary of the EDDB$_F$ values for all derivatives (EDDBσ = EDDB - EDDBπ).

Comp.	**1**	**2**	**3**	**4**	**5**	**6**	**7**	**8**	**9**	**10**
EDDB	5.9225	5.9366	5.9309	5.9299	5.9331	5.9309	5.9214	5.9958	5.9282	5.9357
EDDBπ	4.463	4.5391	4.514	4.5803	4.633	4.6105	4.5673	4.7978	4.6104	4.639
EDDBσ	1.4595	1.3975	1.4169	1.3496	1.3001	1.3204	1.3541	1.198	1.3178	1.2967

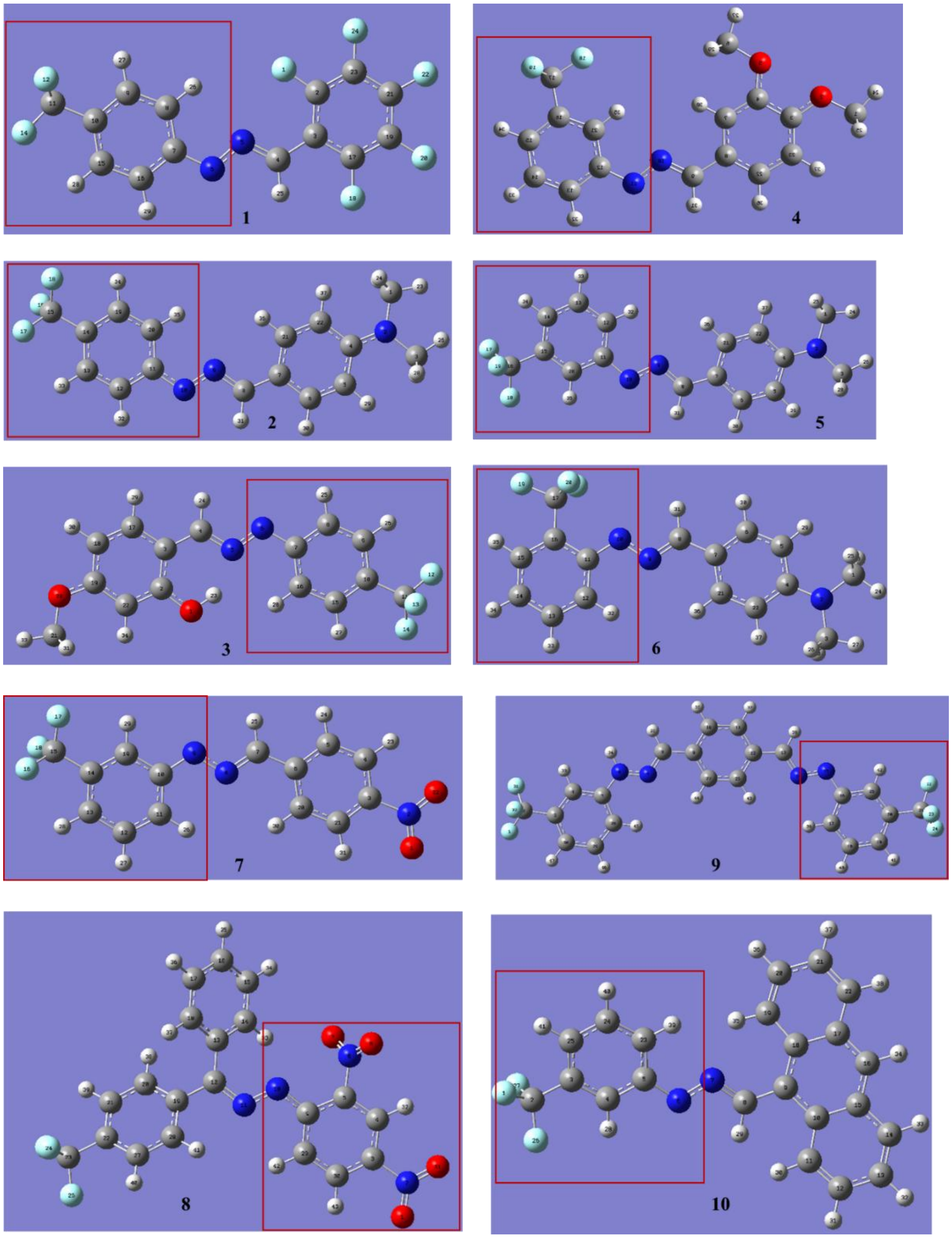

Figure S42. The fragments in the EDDB analysis.

Bibliography

1 a) Baier, A., Kokel, A., Horton, W., Gizińska, E., Pandey, G., Szyszka, R., Török, M. Organofluorine Hydrazone Derivatives as Multifunctional Anti-Alzheimer's Agents with CK2 Inhibitory and Antioxidant Features. *ChemMedChem*, **2021**, *16*, 1927-1932. b) Mastyugin, M., Vlocskó, R. B., Zsengellér, Z. K., Török, B., Török, M. Development of Diaryl Hydrazones for Alleviation of Mitochondrial Oxidative Stress in Preeclampsia. *Journal of Medicinal Chemistry*, **2025**, *68*, 10075-10091.

2 a) Szczepanik, D. W.; Andrzejak, M.; Dyduch, K.; Żak, E.; Makowski, M.; Mazur, G.; Mrozek, J. A Uniform Approach to the Description of Multicenter Bonding. *Physical Chemistry Chemical Physics* **2014**, *16*, 20514–20523. b) Szczepanik, D. W. A New Perspective on Quantifying Electron Localization and Delocalization in Molecular Systems. *Computational and Theoretical Chemistry* **2016**, *1080*, 33–37. c) Szczepanik, D. W.; Andrzejak, M.; Dominikowska, J.; Pawełek, B.; Krygowski, T. M.; Szatylowicz, H.; Solà, M. The Electron Density of Delocalized Bonds (EDDB) Applied for Quantifying Aromaticity. Physical Chemistry Chemical Physics **2017**, *19*, 28970–28981. d) https://aromaticity.uj.edu.pl/eddb.html (last accessed on December 1)
